# New Cleistanthane Diterpenoids from Vellozia pyrantha A.A.Conc and Their Cytotoxic Activity

**DOI:** 10.1021/acsomega.5c02961

**Published:** 2025-05-29

**Authors:** Iago B. F. dos Santos, Antonio G. Ferreira, Tiago Venâncio, Daniel Pereira Bezerra, Milena Botelho Pereira Soares, Valdenizia Rodrigues Silva, Luciano de Souza Santos, Caline G. Ferraz, Floricéa M. Araújo, Paulo R. Ribeiro

**Affiliations:** † Metabolomics Research Group, UFBA, 40170-110 Salvador, Brazil; ‡ Laboratório de Ressonância Magnética Nuclear, UFSCar, 13565-905 São Carlos, Brazil; § Instituto Gonçalo Moniz, Fundação Oswaldo Cruz, 40296-710 Salvador, Brazil

## Abstract

Four new cytotoxic cleistanthane diterpenoids, named pyranthanols A**–**B (**1–2**) and pyranthanones A (**3–4**), were isolated from Vellozia pyrantha A.A.Conc resin, along with four known compounds (**5–8**). These compounds show varying degrees of oxidation with hydroxyl and carbonyl groups at different positions of the cleistanthane core skeleton. Their structures were proposed after careful analysis of ^1^H and ^13^C NMR, HMBC, HMQC, and COSY and NOESY 1D data and comparison with the literature. Pyranthanol B (**2**) and pyranthanones A–B (**3–4**) were active against a lung carcinoma cell line (H-1299) with IC_50_ values varying from 27.57 ± 6.89 to 99.96 ± 22.64 μM, whereas pyranthanol B (**2**) and pyranthanone B (**4**) were active against a human liver carcinoma cell line (HepG2) with IC_50_ values of 36.21 ± 11.97 and 43.63 ± 25.53 μM, respectively. Pyranthanol B (**2**) and pyranthanone A (**3**) were also toxic against a normal lung cell line (MRC-5) with IC_50_ values of 49.99 ± 19.98 and 43.63 ± 43.63 ± 20.25 μM, respectively. The moderate activity observed may suggest these compounds as potential candidates for drug development against carcinoma cell lines.

## Graphical abstract

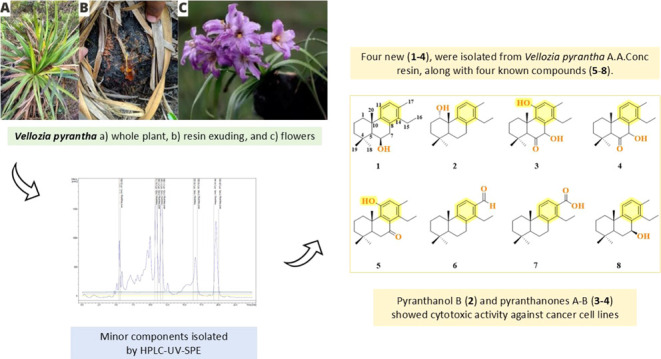

## Introduction

Cleistanthane diterpenoids are a class of naturally occurring compounds primarily found in plants of the genus *Cleistanthus* and are known for their diverse biological activities, including anti-inflammatory, cytotoxic, and antimicrobial properties.^1,2^ Cleistanthol (cleistanthan-8,11,13,15-tetraen-2,3,12-triol) was initially isolated from Cleistanthus schlechteri (Phyllanthaceae), consisting of the first aromatic diterpene with a cleistanthane skeleton.^3^ Other aromatic cleistanthane diterpenoids have been isolated from Excoecaria acerifolia,^4^ Sauropus spatulifolius,^2^ Strophioblachia fimbricalyx,^5^ and Phyllanthus acidus,^6,7^ but they are mainly found in *Vellozia* species.^1,8–13^

The genus *Vellozia* encompasses a group of perennial, flowering plants belonging to the family Velloziaceae, primarily found in the tropical and subtropical regions of South America, especially in mountainous areas of Brazil. These plants are well adapted to harsh environments, such as rocky and arid landscapes, and are known for their remarkable ability to thrive in nutrient-poor soils.^14^ The genus has attracted attention for its ecological significance in biodiverse ecosystems like the Brazilian Cerrado, as well as for potential applications in ornamental horticulture.^15,16^ Herein, we report the isolation of four new cytotoxic cleistanthane diterpenoids named pyranthanols A (**1**–**2**) and pyranthanones A–B (**3–4**), along with four known compounds (**5–8**) from Vellozia pyrantha A.A.Conc resin.

Pyranthanol A (**1**, cleistanthan-8,11,13-trien-6β-ol) (Figure 1) was obtained as a yellow gum, and its molecular formula (C_20_H_30_O) was established by HRESIMS (positive mode) based on the ion peak at *m*/*z* 287.2374 [M + H]^+^ (expected 287.2374) (Supporting Information Figure S1). The ^1^H NMR spectrum of pyranthanol A (**1**) indicated the presence of two aromatic hydrogens at δ_H_ 7.11 (d, 8.1 Hz, 1H) and δ_H_ 7.02 (d, 8.1 Hz, 1H), along with five methyl groups at δ_H_ 2.29 (s, 3H), δ_H_ 1.59 (s, 3H), δ_H_ 1.29 (s, 3H), δ_H_ 1.12 (t, 7.5 Hz, 3H), and δ_H_ 1.06 (s, 3H) (Table 1 and Supporting Information Figures S2–S24), which suggested the presence of a classic cleistanthane core skeleton.^1,8^

**Figure 1:**
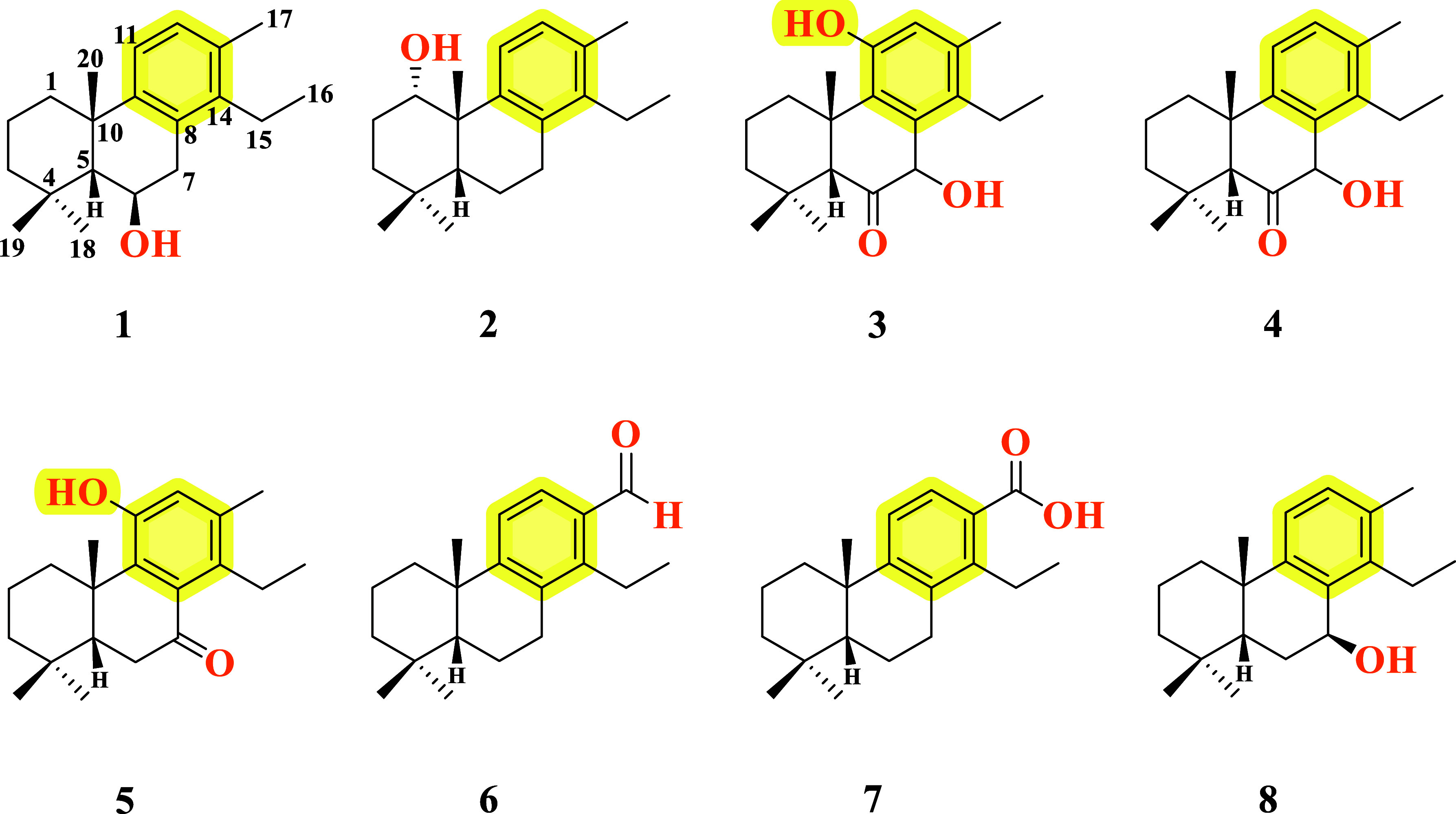
Chemical structures of the cleistanthane diterpenoids isolated from the resin of Vellozia pyrantha.

**Table 1:** NMR Data for Pyranthanol A (**1**) in CDCl_3_

position	δ_H_, mult (J in Hz)	δ_C_, type	HMBC
**1a**	1.37 (ddt, 1.5, 3.3, and 12.8 Hz, 1H)	42.7	19.4, 34.1, 43.0
**1b**	2.19 (ddt, 1.5, 3.3, and 12.8 Hz, 1H)		19.4, 43.0, 52.5
**2a**	1.60 (m, 1H)	19.4	34.1, 37.5, 42.7
**2b**	1.86 (qt, 3.3 and 13.6 Hz, 1H)		34.1, 37.5, 42.7, 43.0
**3a**	1.22 (td, 3.3 and 13.6 Hz, 1H)	43.0	19.4, 23.8, 34.1, 42.7
**3b**	1.47 (m, 1H)		19.4, 42.7, 52.5
**4**		34.1	
**5**	1.40 (s, 1H)	52.5	23.8, 27.2, 33.7, 34.1, 37.5, 42.7, 66.0, 146.9
**6**	4.75 (d, 5.2 Hz, 1H)	66.0	34.1, 37.5, 129.1
**7a**	3.00 (d, 17.4 Hz, 1H)	38.5	52.5, 66.0, 129.1, 140.6, 146.9
**7b**	3.07 (dd, 5.2 × 10^17^ 4 Hz, 1H)		52.5, 66.0, 129.1, 140.6, 146.9
**8**		129.1	
**9**		146.9	
**10**		37.5	
**11**	7.11 (d, 8.1 Hz, 1H)	122.8	37.5, 129.1, 133.0, 140.6
**12**	7.02 (d, 8.1 Hz, 1H)	128.4	19.7, 129.1, 133.0, 140.6, 146.9
**13**		133.0	
**14**		140.6	
**15a**	2.64 (dq, 7.5 and 13.9 Hz, 1H)	22.2	13.0, 129.1, 133.0, 140.6
**15b**	2.61 (dq, 7.5 and 13.9 Hz, 1H)		13.0, 129.1, 133.0, 140.6
**16**	1.12 (t, 7.5 Hz, 3H)	13.0	22.2, 140.6
**17**	2.29 (s. 3H)	19.7	128.4, 133.0, 140.6, 146.9
**18**	1.06 (s. 3H)	33.7	19.4, 23.8, 34.1, 43.0, 52.5
**19**	1.29 (s. 3H)	23.8	33.7, 34.1, 43.0, 52.5
**20**	1.59 (s. 3H)	27.2	37.5, 42.7, 52.5, 146.9

It also showed signals at δ_H_ 2.64 (dq, 7.5 × 10^13^.9 Hz, 1H) and δ_H_ 2.61 (dq, 7.5 × 10^13^.9 Hz, 1H) that have been assigned to the diastereotopic hydrogens of the methylene group attached to the aromatic ring (Table 1 and Supporting Information Figures S2–S24). The signal at δ_H_ = 4.75 (d, 3.0 Hz, 1H) suggested the presence of a hydrogen atom attached to a possible carbinolic carbon. Analysis of the ^13^C NMR spectrum of pyranthanol A (**1**) confirmed the presence of five methyl groups, five methylene groups, two sp^3^ methine groups, two sp^3^ non-hydrogenated carbons, and six carbons of an aromatic ring (Table 1 and Supporting Information Figures S2–S24). A peak at δH 66.0 further supported the presence of a hydroxyl group in the structure of pyranthanol A (**1**). HMBC correlations between H-6 and δ_C_ 34.1 (C-4), δ_C_ 37.5 (C-10), and δ_C_ 129.1 (C-8) and HMBC correlations between H-7 and δ_C_ 129.1 (C-8), δ_C_ 140.6 (C-14), and δ_C_ 146.9 (C-9), along with COSY correlations between H-6/H-5 and H-6/H-7, undoubtedly position the hydroxyl group at C-6 (Figure 2a, Table 1, and Supporting Information Figures S2–S24).

**Figure 2:**
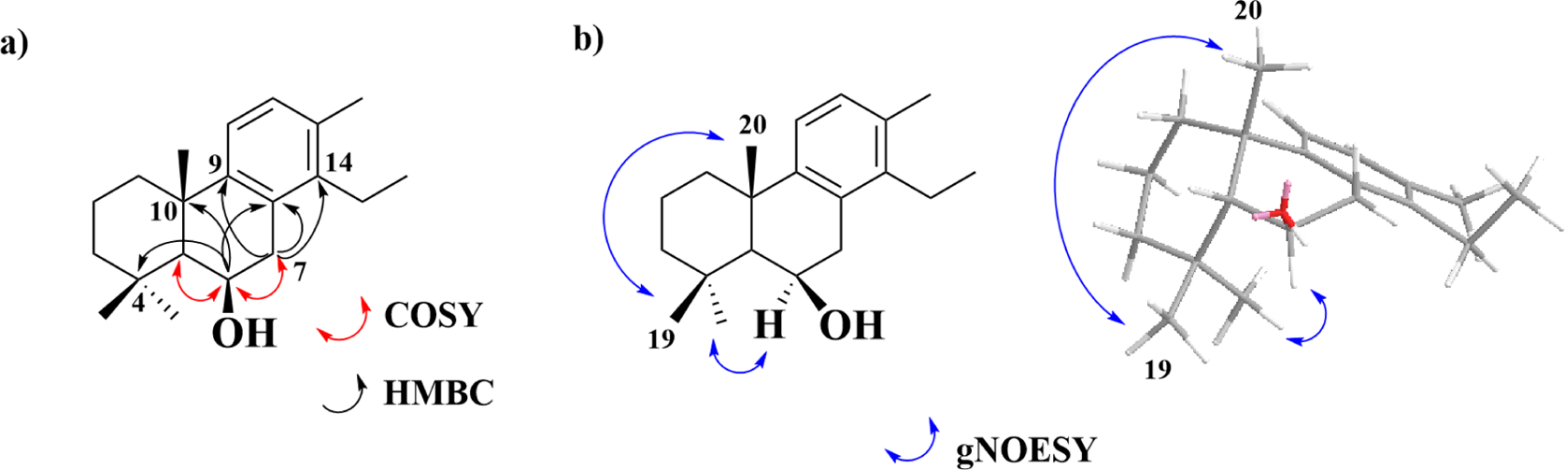
Key 2D NMR correlations of pyranthanol A (**1**). (a) COSY and HMBC correlations and (b) gNOESY correlations.

The relative stereochemistry of the hydroxyl group was established based on the gNOESY correlations between H-19/H-20 and H-6/H-18, confirming that the hydroxyl group at C-6 was on the same side as methyl groups at C-19 and C-20 (Figure 2b and Supporting Information Figures S2–S24).

Furthermore, the relative configuration of C-5 (β) was established by the coupling pattern of H-5 and H-6. H-5 does not couple with H-6 and it appears in the ^1^H NMR spectra as a singlet, which would only be possible if the dihedral torsion angle between H-5 and H-6 is close to 90 deg; therefore, H-5 has a β configuration (Figure 3).

**Figure 3:**
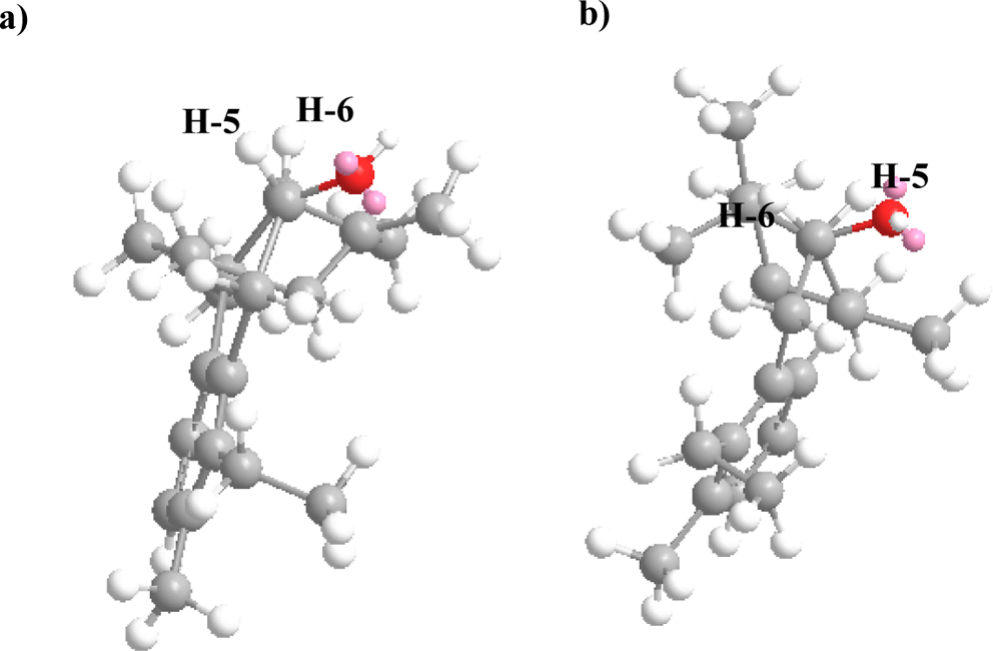
Dihedral torsion angle between H-5 and H-6 of pyranthanol A (**1**) influenced by the relative configuration of H-5 as α (a) or β (b).

Pyranthanol B (**2**, cleistanthan-8,11,13-trien-1α-ol) (Figure 1) was obtained as a yellow gum, and its molecular formula (C_20_H_30_O) was established by HRESIMS (positive mode) based on the ion peak at *m*/*z* 287.2375 [M + H]^+^ (expected 287.2374) (Supporting Information Figure S26). Pyranthanol B (**2**) is an isomer of pyranthanol A (**1**) with very similar signal patterns in the ^1^H and ^13^C NMR spectra (Table 2 and Supporting Information Figures S27–S51). The ^1^H NMR spectrum of pyranthanol B (**2**) indicated the presence of two aromatic hydrogens at δ_H_ 7.08 (d, 8.0 Hz, 1H) and δ_H_ 7.02 (d, 8.0 Hz, 1H), along with five methyl groups at δ_H_ 2.29 (s, 3H), δ_H_ 1.23 (s, 3H), δ_H_ 1.10 (t, 7.6 Hz, 3H), δ_H_ 1.00 (s, 3H), and δ_H_ 0.94 (s, 3H) (Table 2 and Supporting Information Figures S27–S51), typical of the cleistanthane core skeleton.^1,8^ It also showed signals at δ_H_ 2.61 (q, 7.6 Hz, 1H) that have been assigned to the methylene group attached to the aromatic ring (Table 2 and Supporting Information Figures S27–S51). Curiously, this methylene group appears as diastereotopic protons (dq) if there is a substituent at C-6 or C-7. The signal at δ_H_ 4.34 (t, 2.7 Hz, 1H) suggested the presence of a hydrogen atom attached to a possible carbinolic carbon. A peak at δH 72.1 further supported the presence of a hydroxyl group in the structure of pyranthanol B (**2**). Analysis of the ^13^C NMR spectrum of pyranthanol B (**2**) confirmed the presence of five methyl groups, five methylene groups, two sp^3^ methine groups, two sp^3^ non-hydrogenated carbons, and six carbons of an aromatic ring. HMBC correlations between H-1 and δ_C_ 25.5 (C-20), δ_C_ 34.2 (C-2), δ_C_ 43.1 (C-5), and δ_C_ 143.0 (C-9) undoubtedly position the hydroxyl group at C-1 (Figure 4a, Table 2, and Supporting Information Figures S27–S51).

**Table 2:** NMR Data for Pyranthanol B (**2**) in CDCl_3_

position	δ_H_, mult (J in Hz)	δ_C_, type	HMBC
**1**	4.34 (t, 2.7 Hz, 1H)	72.1	25.5, 34.2, 43.1, 143.0
**2a**	1.24 (m, 1H)	34.2	21.6, 24.4, 33.1,43.9, 72.1
**2b**	1.75 (m, 1H)		21.6, 25.5, 33.1, 43.9, 72.1
**3a**	1.80 (m, 1H)	24.4	21.6, 33.1, 43.1, 72.1
**3b**	2.04 (m, 1H)		18.7, 34.2
**4**		33.1	
**5**	1.77 (m, 1H)	43.1	18.7, 21.6, 25.5, 28.1, 33.1, 43.9, 72.1, 143.0
**6a**	1.68 (m, 1H)	18.7	28.1, 43.1
**6b**	1.94 (m, 1H)		28.1, 33.1, 43.1, 43.9, 135.3
**7a**	2.72 (m, 1H)	28.1	18.7, 22.2, 43.1, 135.3, 143.0
**7b**	2.95 (dd, 4.9 × 10^17^ 2 Hz, 1H)		18.7, 43.1, 135.3, 143.0
**8**		135.3	
**9**		143.0	
**10**		43.9	
**11**	7.08 (d, 8.0 Hz, 1H)	121.3	43.9, 133.4, 135.3
**12**	7.02 (d, 8.0 Hz, 1H)	128.6	19.4, 133.4, 135.3, 141.5, 143.0
**13**		133.4	
**14**		141.5	
**15**	2.61 (q, 7.6 Hz, 2H)	22.2	12.9, 133.4, 135.3, 141.5
**16**	1.10 (t, 7.6 Hz, 3H)	12.9	22.2, 141.5
**17**	2.29 (s, 3H)	19.4	128.6, 133.4, 141.5
**18**	1.00 (s, 3H)	33.1	21.6, 33.1, 34.2, 43.1
**19**	0.94 (s, 3H)	21.6	33.1, 34.2, 43.1
**20**	1.23 (s, 3H)	25.5	43.9, 72.1, 143.0

**Figure 4:**
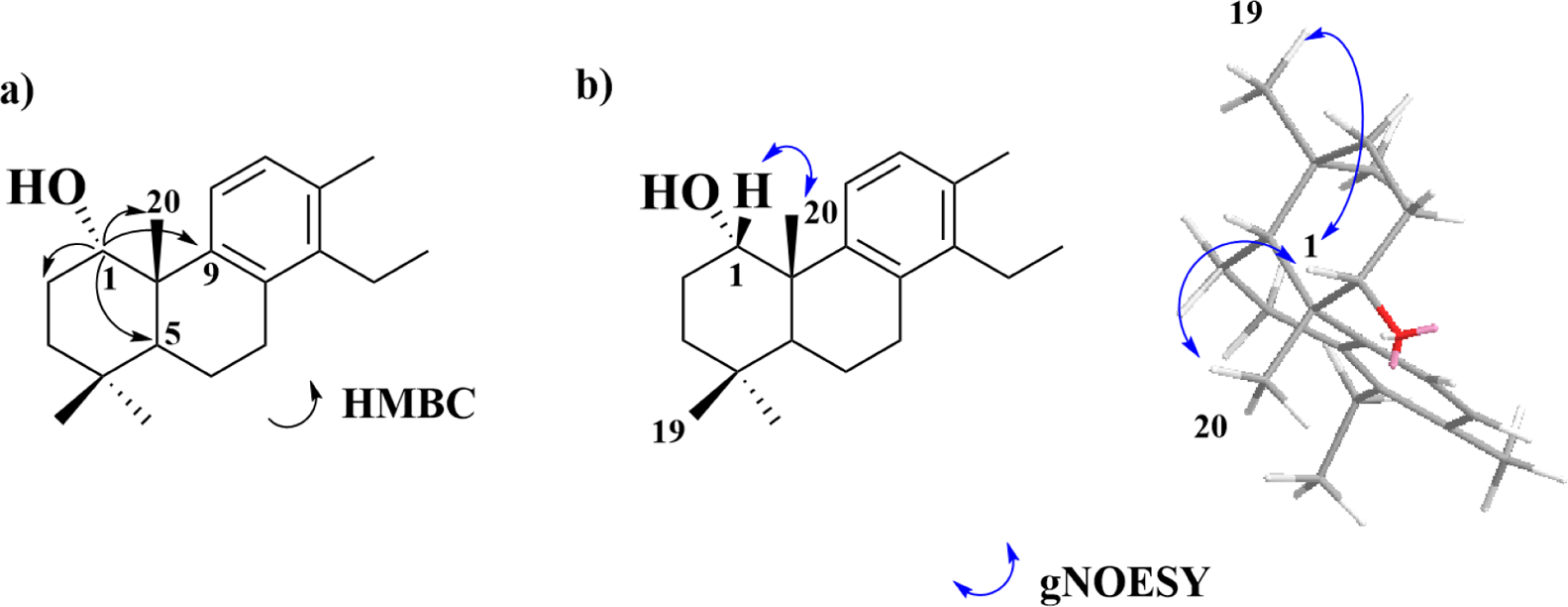
Key 2D NMR correlations of pyranthanol B (**2**): (a) HMBC and (b) gNOESY.

Finally, the relative stereochemistry of the hydroxyl group was established based on the gNOESY correlation between H-1 and H-20, confirming that the hydroxyl group at C-1 was on the opposite side of the methyl group at C-20 (Figure 4b and Supporting Information Figures S27–S51). Furthermore, the correlation between H-1 and H-19 indicates that the methyl group C-19 is on the same side as the methyl group at C-20.

Pyranthanone A (**3**, 7,11-dihydroxy-cleistanthan-8,11,13-trien-6-one) (Figure 1) was obtained as a yellow gum and its molecular formula (C_20_H_28_O_3_) was established by HRESIMS (positive mode) based on the ion peak at *m*/*z* 317.2118 [M + H]^+^ (expected 317.2117) (Supporting Information Figure S52). The ^1^H NMR spectrum of pyranthanone A (**3**) indicated the presence of only one single aromatic hydrogen at δ_H_ 6.52 (s, 1H), along with five methyl groups at δ_H_ 2.25 (s, 3H), δ_H_ 1.40 (s, 3H), δ_H_ 1.28 (s, 3H), δ_H_ 1.15 (t, 7.5 Hz, 3H), and δ_H_ 1.03 (s, 3H) (Table 3 and Supporting Information Figures S53–S67), typical of the cleistanthane core skeleton.^1,8^ It also showed signals at δ_H_ 2.87 (dq, 7.6 × 10^14^ 8 Hz, 1H) and δ_H_ 2.59 (dq, 7.6 × 10^14^ 8 Hz, 1H) that have been assigned to the diastereotopic hydrogens of the methylene group attached to the aromatic ring (Table 3 and Supporting Information Figures S53–S67). The signal at δ_H_ 4.60 s (s, 1H) suggested the presence of a hydrogen atom attached to a possible carbinolic carbon. A peak at δH 72.9 further supported the presence of a hydroxyl group in the structure of pyranthone A (**3**). Analysis of the ^13^C NMR spectrum of pyranthanone A (**3**) confirmed the presence of five methyl groups, four methylene groups, two sp^3^ methine groups, two sp^3^ non-hydrogenated carbons, and six carbons of an aromatic ring. Additionally, the ^13^C NMR spectrum revealed the presence of a carbonyl group at δ_C_ = 209.4.

**Table 3:** NMR Data for Pyranthanone A (**3**) in CDCl_3_

position	δ_H_, mult (J in Hz)	δ_C_, type	HMBC
**1a**	1.48 (td, 3.8 and 13.3 Hz, 1H)	37.0	20.3, 41.8, 45.7, 57.8
**1b**	3.27 (dt, 3.0 and 13.3 Hz, 1H)		19.0, 41.8, 45.7, 57.8
**2a**	1.58 (m, 1H)	19.0	22.3, 32.3, 45.7
**2b**	1.77 (qt, 3.8 and 13.9 Hz, 1H)		32.3, 37.0, 41.8, 45.7
**3a**	1.21 (m, 1H)	41.8	22.3, 32.3, 32.5, 37.0
**3b**	1.40 (m, 1H)		32.3, 32.5, 41.8, 57.8
**4**		32.3	
**5**	3.34 (s, 1H)	57.8	20.3, 22.3, 32.3, 37.0, 41.8, 45.7, 132.7, 209.4
**6**		209.4	
**7**	4.60 (s, 1H)	72.9	132.7, 134.1, 136.4, 209.4
**8**		134.1	
**9**		132.7	
**10**		45.7	
**11**		151.0	
**12**	6.52 (s, 1H)	120.0	19.4, 132.7, 136.3, 136.4, 151.0
**13**		136.3	
**14**		136.4	
**15a**	2.59 (dq, 7.6 and 14.8 Hz, 1H)	21.4	14.9, 134.1, 136.3, 136.4
**15b**	2.87 (dq, 7.6 and 14.8 Hz, 1H)		14.9, 134.1, 136.3, 136.4
**16**	1.15 (t, 7.6 Hz, 3H)	14.9	21.4, 136.4
**17**	2.25 (s, 3H)	19.4	120.0, 134.1, 136.3, 136.4
**18**	1.03 (s, 3H)	32.5	22.3, 32.3, 41.8, 57.8
**19**	1.40 (s, 3H)	22.3	32.3, 32.5, 41.8, 57.8
**20**	1.28 (s, 3H)	20.3	37.0, 45.7, 57.8, 132.7

HMBC correlations between H-5 and δ_C_ 209.4 (C-6), along with correlations between H-7 and δ_C_ 57.8 (C-5), δ_C_ 134.1 (C-8), δ_C_ 136.4 (C-14), δ_C_ 132.7 (C-9), and δ_C_ 209.4 (C-6), undoubtedly position the hydroxyl group at C-7 and the carbonyl group at C-6 (Figure 5a, Table 3, and Supporting Information Figures S53–S67). Furthermore, HMBC correlations between H-17 and δ_C_ 120.0 (C-12) and between H-12 and δ_C_ 136.4 (C-14) and δ_C_ 19.4 (C-17) undoubtedly position the hydroxyl group at C-11 (Figure 5a, Table 3, and Supporting Information Figures S53–S67).

**Figure 5:**
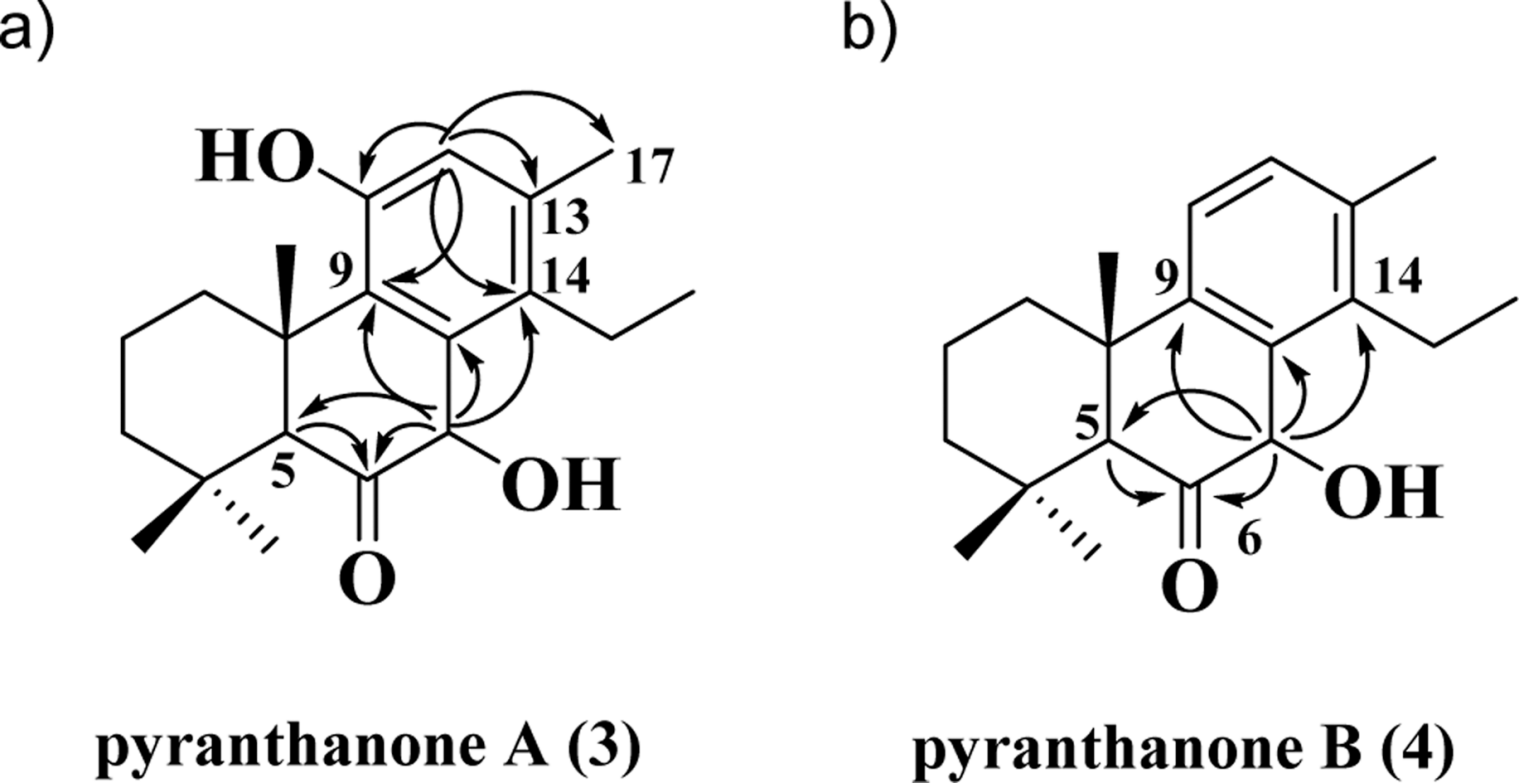
Key HMBC correlations of pyranthanones A–B (**3–4**).

Pyranthanone B (**4**, 7-hydroxy-cleistanthan-8,11,13-trien-6-one) (Figure 1) was obtained as a yellow gum, and its molecular formula (C_20_H_28_O_2_) was established by HRESIMS (positive mode) based on the ion peak at *m*/*z* 301.2169 [M + H]^+^ (expected 301.2167) (Supporting Information Figure S68). The ^1^H NMR spectrum of pyranthanone B (**4**) indicated the presence of two aromatic hydrogens at δ_H_ 7.16 (d, 8.2 Hz, 1H) and δ_H_ 7.11 (d, 8.2 Hz, 1H), along with five methyl groups at δ_H_ 2.33 (s, 3H), δ_H_ 1.39 (s, 3H), δ_H_ 1.20 (t, 7.5 Hz, 3H), δ_H_ 1.12 (s, 3H), and δ_H_ 1.05 (s, 3H) (Table 4 and Supporting Information Figures S69–S83), typical of the cleistanthane core skeleton.^1,8^ It also showed signals at δ_H_ 2.92 (dq, 7.6 × 10^14^.9 Hz, 1H) and δ_H_ 2.69 (dq, 7.6 × 10^14^.9 Hz, 1H) that have been assigned to the diastereotopic hydrogens of the methylene group attached to the aromatic ring (Table 4 and Supporting Information Figures S69–S83). The signal at δ_H_ 4.74 (d, 4.4 Hz, 1H) suggested the presence of a hydrogen atom attached to a possible carbinolic carbon. A peak at δH 72.5 further supported the presence of a hydroxyl group in the structure of pyranthanone B (**4**). Analysis of the ^13^C NMR spectrum of pyranthanone B (**4**) confirmed the presence of five methyl groups, four methylene groups, two sp^3^ methine groups, two sp^3^ non-hydrogenated carbons, and six carbons of an aromatic ring. Additionally, the ^13^C NMR spectrum revealed the presence of a carbonyl group at δ_C_ = 208.7.

**Table 4:** NMR Data for Pyranthanone B (**4**) in CDCl_3_

position	δ_H_, mult (J in Hz)	δ_C_, type	HMBC
**1a**	1.67 (m, 1H)	39.0	19.0, 26.7, 41.9, 43.3, 147.7
**1b**	2.36 (m, 1H)		19.0, 26.7, 41.9, 43.3, 57.3
**2a**	1.66 (m, 1H)	19.0	32.0, 39.0, 41.9
**2b**	1.77 (tt, 3.3 and 13.5 Hz, 1H)		32.0, 39.0, 41.9
**3a**	1.19 (m, 1H)	41.9	19.0, 21.9, 32.0, 39.0, 57.3
**3b**	1.43 (m, 1H)		19.0, 21.9, 32.0, 39.0, 57.3
**4**		32.0	
**5**	3.15 (s, 1H)	57.3	21.9, 26.7, 32.0, 39.0, 41.9, 43.3, 147.7, 208.7
**6**		208.7	
**7**	4.74 (d, 4.4 Hz, 1H)	72.5	57.3, 131.6, 143.5, 147.7, 208.7
**8**		131.6	
**9**		147.7	
**10**		43.3	
**11**	7.11 (d, 8.2 Hz, 1H)	122.0	43.3, 131.7, 133.0, 135.6
**12**	7.16 (d, 8.2 Hz, 1H)	131.7	19.1, 143.5, 147.7
**13**		135.3	
**14**		143.5	
**15a**	2.69 (dq, 7.6 and 14.9 Hz, 1H)	22.0	14.7, 131.6, 135.3, 143.5
**15b**	2.92 (dq, 7.6 and 14.9 Hz, 1H)		14.7, 131.6, 135.3, 143.5
**16**	1.20 (t, 7.6 Hz, 3H)	14.7	22.0, 143.5
**17**	2.33 (s, 3H)	19.1	131.7, 135.3, 143.5
**18**	1.05 (s, 3H)	32.3	21.9, 32.0, 41.9, 57.3
**19**	1.39 (s, 3H)	21.9	32.0, 41.9, 57.3
**20**	1.12 (s, 3H)	26.7	39.0, 41.9, 57.3, 147.7

HMBC correlations between H-5 and δ_C_ 208.7 (C-6), along with correlations between H-7 and δ_C_ 57.3 (C-5), δ_C_ 131.6 (C-8), δ_C_ 143.5­(C-14), δ_C_ 147.7 (C-9), and δ_C_ 208.7 (C-6), undoubtedly position the hydroxyl group at C-7 and the carbonyl group at C-6 (Figure 5b, Table 4, and Supporting Information Figures S69–S83).

Compound 11-hydroxy-cleistanthan-8,11,13-trien-7-one (**5**) has been isolated from Vellozia nivea,^17^ compounds cleistanthan-8,11,13-trien-17-al (**6**, veadeiral) and cleistanthan-8,11,13-trien-17-oic acid (**7**, veadeiroic Acid) have been isolated from Vellozia flavicans,^9,11,18^ whereas compound cleistanthan-8,11,13-trien-7β-ol (**8**) has been isolated from Vellozia declinans^10^ (Supporting Information Figures S99–S150). This is, however, the first report of the isolation of these four compounds from V. pyrantha. Taxonomists have encountered controversy regarding the division of genera and subfamilies within the Velloziaceae family, as the classification heavily relies on floral morphology and leaf anatomy. Consequently, chemotaxonomic markers could offer significant assistance to taxonomists in differentiating genera and subfamilies within this family.^8^ Given the highly limited distribution and presence of these cleistanthane diterpenoids in *Vellozia* species, we propose that they could serve as chemotaxonomic markers for this genus.

Cancer develops due to the uncontrolled growth of abnormal cells in a given organism once normal cell regulation mechanisms fail, causing cells to divide and form tumors rapidly. Cancer is a problem of public health worldwide, accounting for nearly 10 million deaths annually, with higher mortality in developing countries due to limited access to early detection and effective treatments.^19^ New cancer therapies are revolutionizing treatment by offering more targeted and personalized approaches. In this context, plants are important allies, providing natural compounds that can be used alone or combined with chemotherapy and immunotherapy.^20^ Cleistanthane diterpenoids possess cytotoxic activity against several cancer cell lines. For example, three cleistanthane diterpenoids isolated from P. acidus (L.) Skeels showed moderate cytotoxic activity against five cancer cell lines (HL-60, A549, SMMC-7721, MCF-7, and SW480).^21^ Herein, we assessed the cytotoxic activity of compounds **1–4** against a lung carcinoma cell line (H-1299), a human liver carcinoma cell line (HepG2), and a normal lung cell line (MRC-5) (Table 5).

**Table 5:** Cytotoxic Activity of Cleistanthane Diterpenoids from V. pyrantha ^a^

compounds	H-1299	selective index	HepG2	selective index	MRC-5
pyranthanolB (**2**)	27.57 ± 6.89	1.81	36.21 ± 11.97	1.38	49.99 ± 19.98
pyranthanone A (**3**)	47.89 ± 10.65	0.91	43.63 ± 25.53	1.00	43.63 ± 20.25
pyranthanoneB (**4**)	99.96 ± 22.64	highly selective	>50	>50	>50
doxorubicin	0.76 ± 0.25	2.45	0.17 ± 0.07	10.94	1.86 ± 0.69

a Results are expressed in μM. Lung carcinoma cell line (H-1299), a human liver carcinoma cell line (HepG2), and a normal lung cell line (MRC-5).

Pyranthanol B (**2**), pyranthanone A (**3**), and pyranthanone B (**4**) were active against a lung carcinoma cell line (H-1299) with IC_50_ values varying from 27.57 ± 6.89 to 99.96 ± 22.64 μM, whereas pyranthanol B (**2**) and pyranthanone A (**3**) were active against a human liver carcinoma cell line (HepG2) with IC_50_ values of 36.21 ± 11.97 and 43.63 ± 25.53 μM, respectively. Pyranthanol B (**2**) and pyranthanone A (**3**) were also toxic against a normal lung cell line (MRC-5) with IC_50_ values of 49.99 ± 19.98 and 43.63 ± 20.25 μM, respectively. Pyranthanol A (**2**) was not active, whereas compounds **5–8** were not tested. Although the activities are modest compared to the control drug doxorubicin, it is worth noting that the cytotoxicity of pyranthanone B (**4**) was selective to the lung carcinoma cell line (H-1299), while not being toxic to the normal lung cell line (MRC-5). However, pyranthanol B (**2**) and pyranthanone A (**3**) showed a broader spectrum of cytotoxicity, even being toxic to the normal lung cell line (MRC-5). Pyranthanol B (**2**) and pyranthanone A (**3**) are moderately selective to the cancer lines, whereas pyranthanone B (**4**) is highly selective toward the lung carcinoma cell line (H-1299), since ideally the drug should kill the cancer cells, but it should not affect the normal cells (Table 5).

## Experimental Section

### General Experimental Procedures

High-performance liquid chromatography (HPLC) was performed on a chromatograph (1200 series; Agilent GmbH) equipped with a quaternary pump (G1311A) and a degasser (G1322A), a variable wavelength diode array detector (G1315D), and an autosampler (Bruker Biospin GmbH). The LC system was controlled by HyStar 2.3 software (Bruker). A Knauer (K120 Knauer Smartline Pump Control 100, Bruker Daltonik GmbH, V01.11) makeup pump diluted the postcolumn flow with water before the peaks were trapped using a Prospekt 2 SPE unit.^22^ Mass spectrometry was analyzed on an ultraperformance liquid chromatograph (Agilent 1290 Infinity II) coupled to an Agilent G6545B Q-TOF mass spectrometer equipped with an electrospray ionization (ESI) source and on a liquid chromatography system (Prominence, Shimadzu Co., Japan) coupled to a quadrupole time-of-flight mass spectrometer microTOF II, Bruker Daltonics, Germany. 1D-NMR (^1^H, ^13^C) and 2D-NMR (COSY, HSQC, HMBC, and NOESY) analyses were obtained on a 14.1T Bruker AVANCE III equipment with a 5 mm TCI cryoprobe.^23^

### Plant Material

V. pyrantha A.A.Conc naturally exudes a resin from its stem that was manually collected at the Chapada Diamantina National Park in Palmeiras (Bahia, Brazil) and stored at −20 °C until further analysis. This is a relatively new species that has been identified and characterized by Conceição et al.^24^ This species and its use have been registered at the “Sistema Nacional de Gestão do Patrimônio Genético e do Conhecimento Tradicional Associado - SISGEN” under the number AA9C491.

### Extraction and Isolation

V. pyrantha resin (18 g) was initially subjected to column chromatography (60 × 10 cm, Merck silica gel 230–400 mesh) eluting with a gradient of hexane and ethyl acetate mixtures to produce 7 fractions (Vpyr01–Vpyr07). Fraction Vpyr01 (5.32 g) was further subjected to column chromatography (60 × 10 cm, Merck silica gel 230–400 mesh) eluting with a gradient of hexane and dichloromethane in increasing polarity to produce 15 fractions called Vpyr01–01 to Vpyr01–15.

Fraction Vpyr01–03 (45 mg) was subjected to preparative thin layer chromatography, using hexane and dichloromethane (7:3) as an eluent and silica as a stationary phase to produce three subfractions. Subfraction 1 contained a mixture of compounds **6** and **7** (12 mg). Subfraction 2 was injected in a reverse-phase HPLC-UV-SPE system equipped with a Kromasil 100-5C18 (250 × 4.6 mm) column and eluted with water and acetonitrile (flow of 0.5 mL min^–1^) to afford compounds **1** (<1 mg) and **8** (<1 mg).

Fraction Vpyr01–04 (61 mg) was subjected to preparative thin layer chromatography, using hexane and dichloromethane (7:3) as an eluent and silica as the stationary phase to produce three subfractions. Subfraction 1 was injected in a reverse-phase HPLC-UV-SPE system equipped with a Kromasil 100-5C18 (250 × 4.6 mm) column and eluted with water and acetonitrile (flow of 0.5 mL min^–1^) to afford compounds **1** (<1 mg) and **2** (<1 mg).

Fraction Vpyr01–05 (700 mg) was subjected to column chromatography (60 × 10 cm, Merck silica gel 230–400 mesh) eluting with a gradient of hexane and dichloromethane mixtures to produce ten subfractions. Subfraction 2 contained a solid that was recrystallized in methanol to afford compound **5** (4 mg). Subfraction 4 was injected in a reverse-phase HPLC-UV-SPE system equipped with a Kromasil 100-5C18 (250 × 4.6 mm) column and eluted with water and acetonitrile (flow of 0.5 mL min^–1^) to afford compound **4** (<1 mg), whereas HPLC-UV-SPE analysis of subfraction 5 produced compound **3** (<1 mg).

### Cytotoxicity Assay

The cytotoxicity assay was conducted following the methodology outlined previously.^1^ We utilized two tumor cell lines: lung carcinoma cell line (H-1299), a human liver carcinoma cell line (HepG2), along with one nontumor cell line, MRC-5 (human lung fibroblast). To evaluate the cell viability, we employed the alamarBlue assay. The extracts were tested at a final concentration of 50 μg mL^–1^, with doxorubicin serving as the positive control.

## Supplementary Material



## Notes

NMR and high-resolution mass spectra of compounds **1–8** (PDF)
