# Supplementary material for: New Cleistanthane Diterpenoids from Vellozia pyrantha A.A.Conc and Their Cytotoxic Activity
Source: ACS Omega. 2025 May 29;10(22):23791–7. doi: 10.1021/acsomega.5c02961 (PMC12163629; doi:10.1021/acsomega.5c02961)
Supplement: Supplementary file 1 [file ao5c02961_si_001.pdf]

New cleistanthane diterpenoids from *Vellozia pyrantha* A.A.Conc and their cytotoxic activity

*Iago B. F. dos Santos[a], Antonio G. Ferreira[b], Tiago Venâncio[b], Daniel Pereira Bezerra[c], Milena Botelho Pereira Soares[c], Valdenizia Rodrigues Silva[c], Luciano de Souza Santos[c], Caline G. Ferraz[a], Floricéa M. Araújo[a], and Paulo R. Ribeiro[a, *]*

[a]Metabolomics Research Group, UFBA, Salvador, Brazil

[b]Laboratório de Ressonância Magnética Nuclear, UFSCar, São Carlos, Brazil

[c]Instituto Gonçalo Moniz, Fundação Oswaldo Cruz, Salvador, Brazil

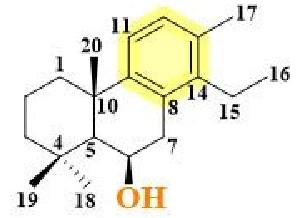

1

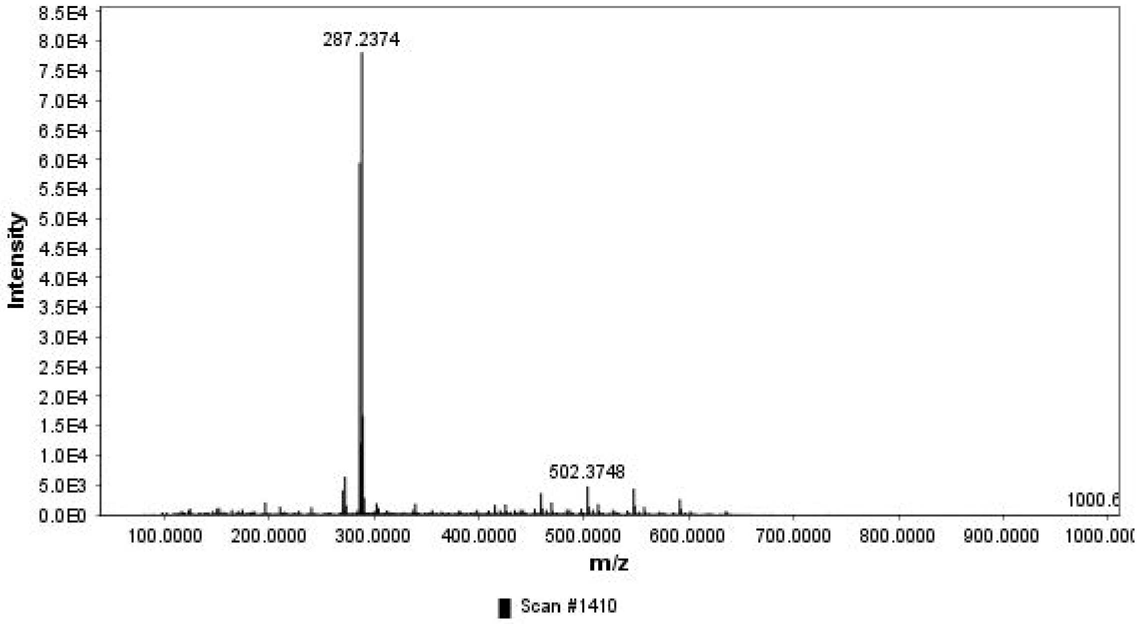

Supplementary Figure 1. High resolution mass spectrum of Pyranthanol A **(1)**.

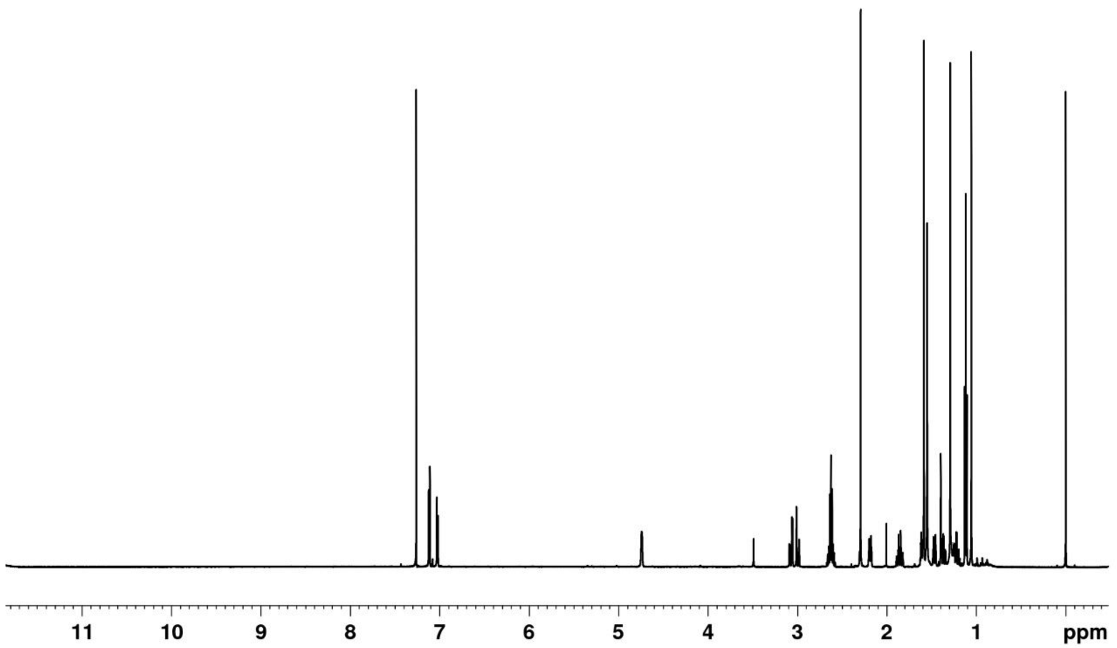

Supplementary Figure 2. ^{1}H NMR spectrum of Pyranthanol A **(1)** [600 MHz, $CDCl_3$, ppm].

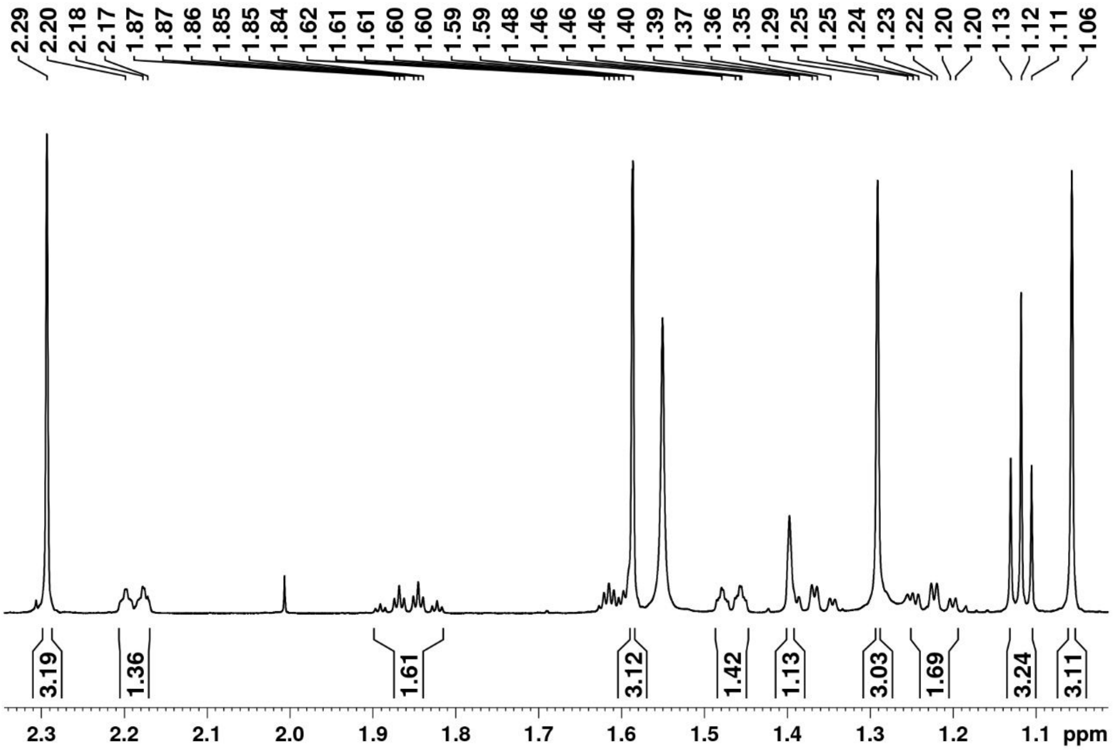

Supplementary Figure 3. ^{1}H NMR spectrum of Pyranthanol A **(1)** [600 MHz, $CDCl_3$, ppm].

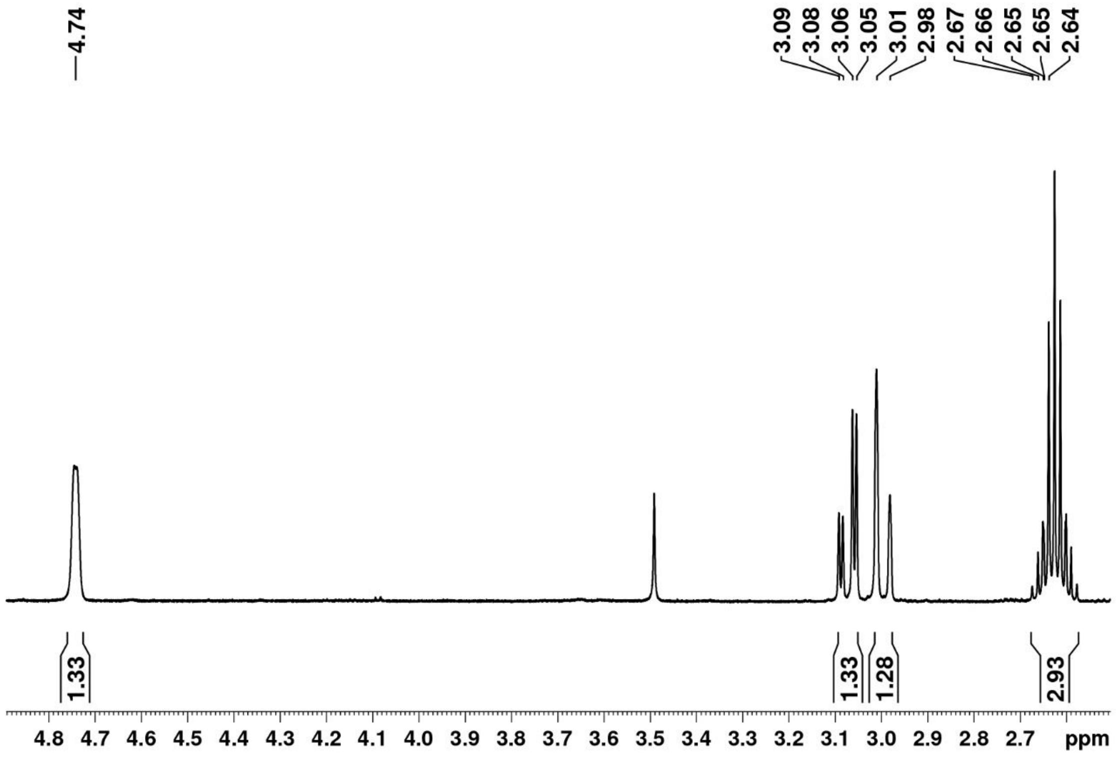

Supplementary Figure 4. ^{1}H NMR spectrum of Pyranthanol A **(1)** [600 MHz, $CDCl_3$, ppm].

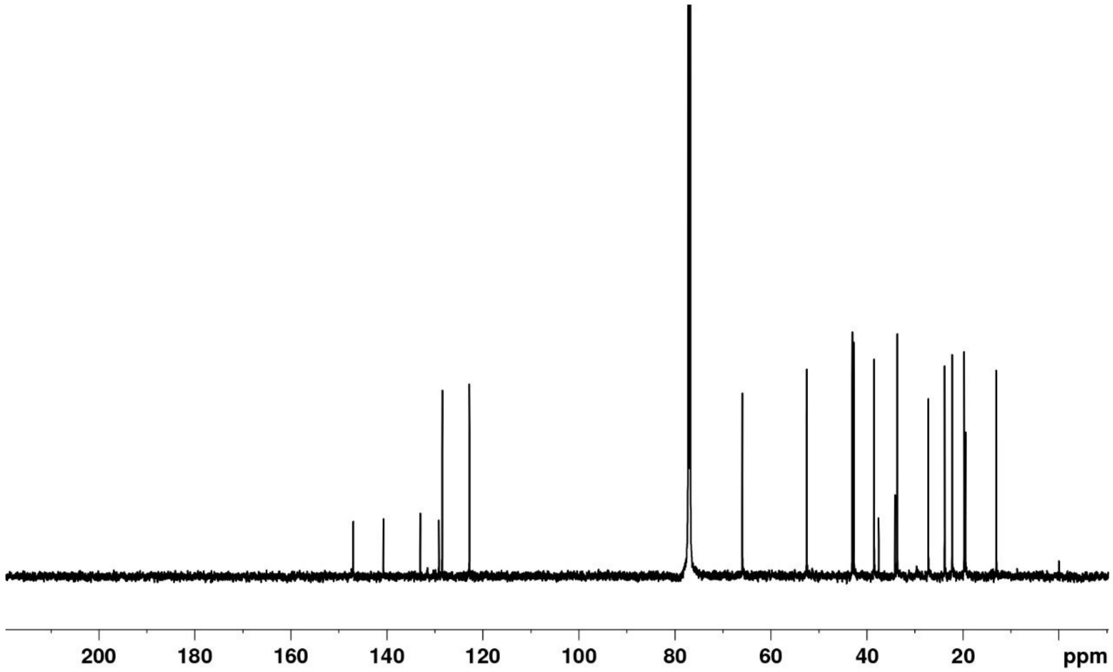

Supplementary Figure 5. ^{13}C NMR spectrum of Pyranthanol A **(1)** [150 MHz, $CDCl_3$, ppm].

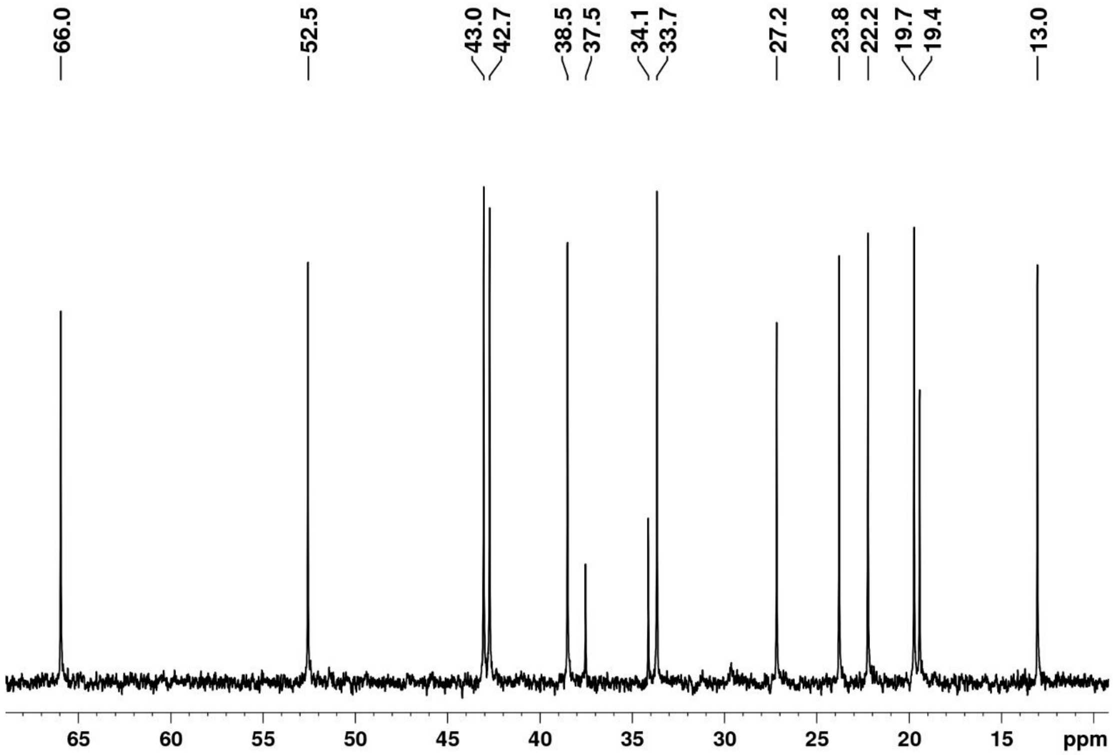

Supplementary Figure 6. ^{13}C NMR spectrum of Pyranthanol A **(1)** [150 MHz, $CDCl_3$, ppm].

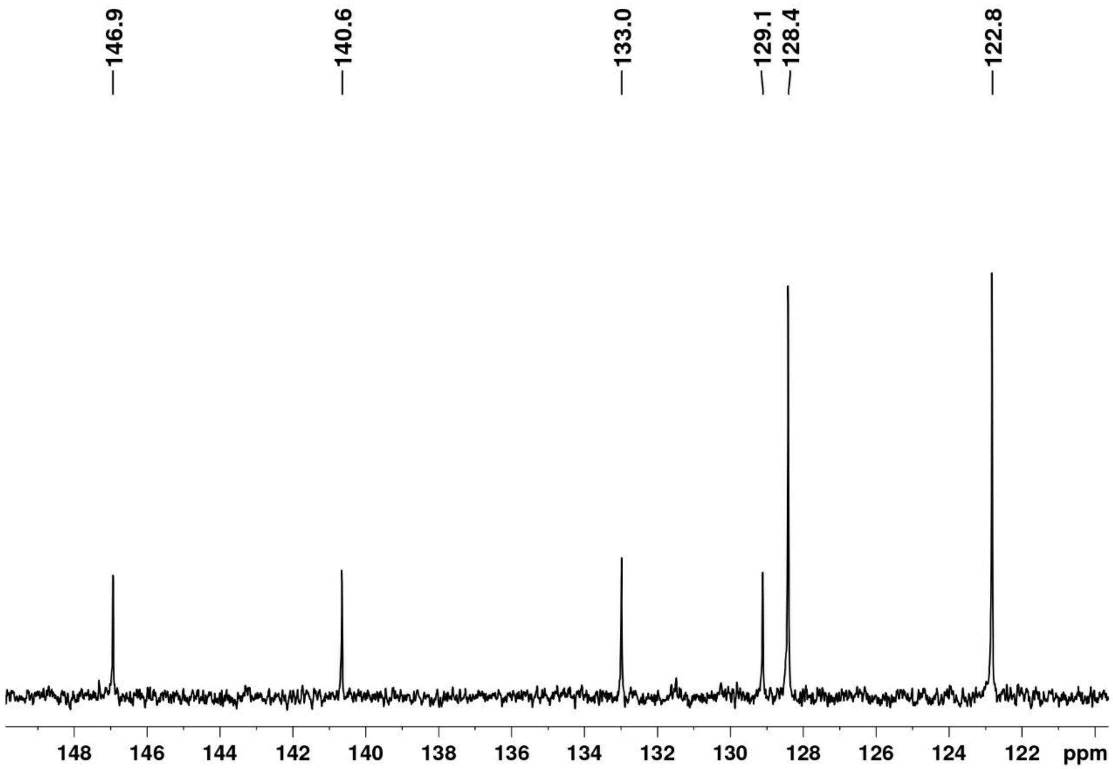

Supplementary Figure 7. ^{13}C NMR spectrum of Pyranthanol A **(1)** [150 MHz, $CDCl_3$, ppm].

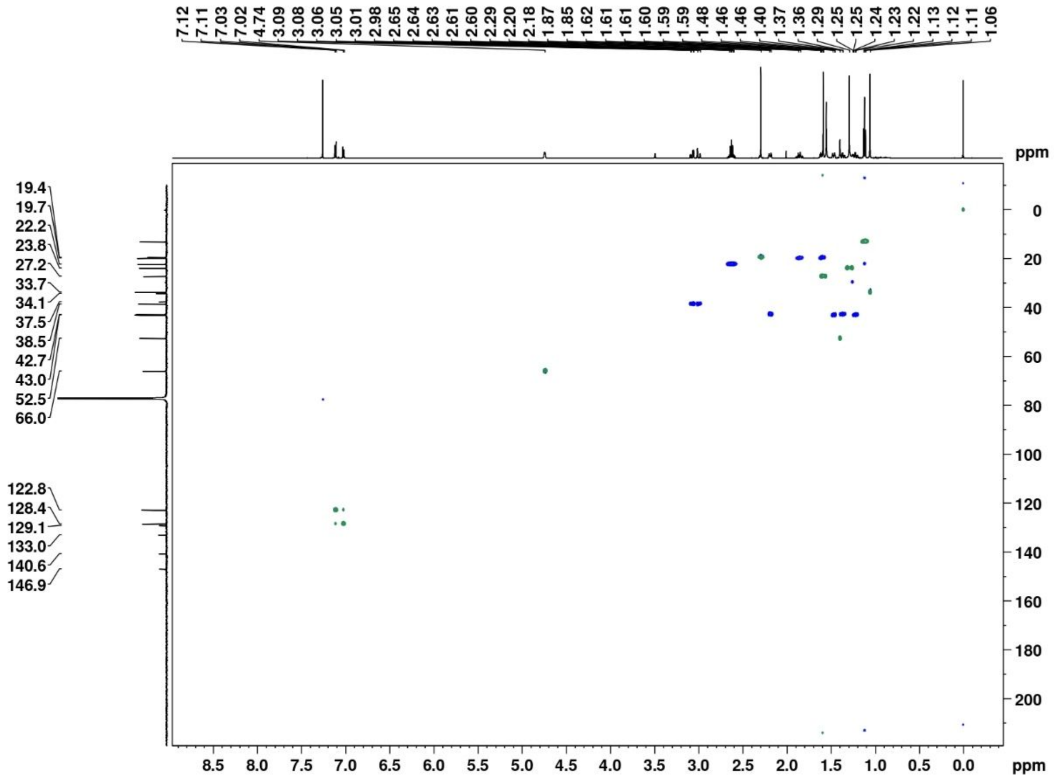

Supplementary Figure 8. HSQC NMR spectrum of Pyranthanol A **(1)** [600 MHz, $CDCl_3$, ppm].

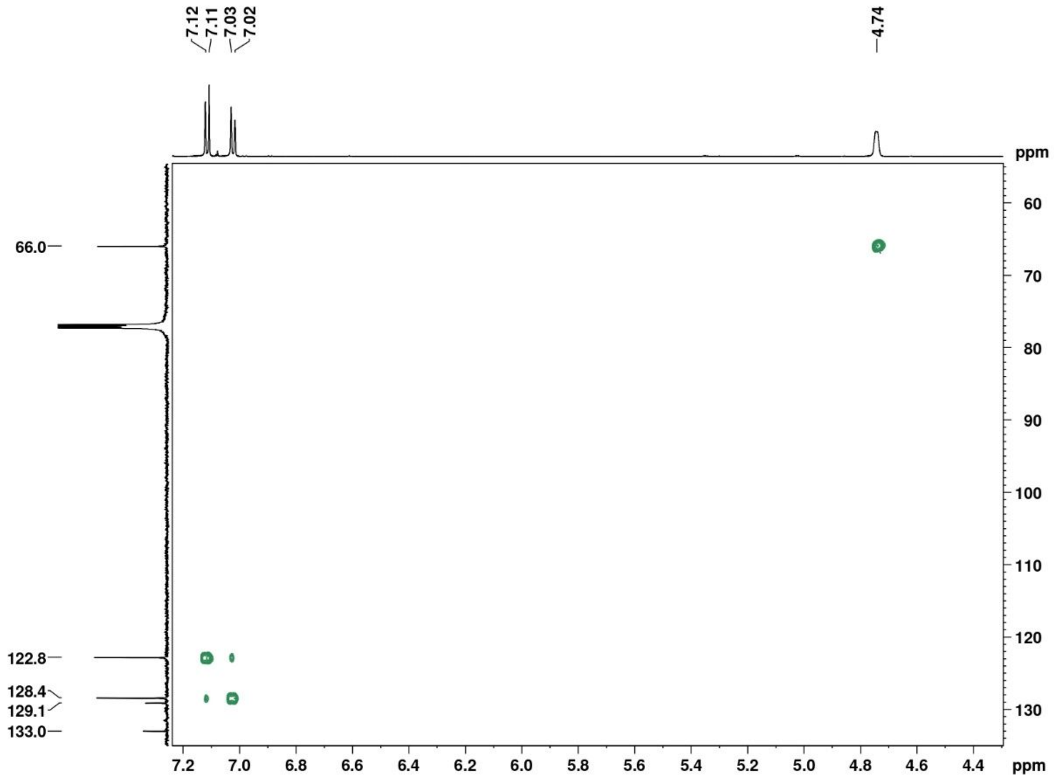

Supplementary Figure 9. HSQC NMR spectrum of Pyranthanol A **(1)** [600 MHz, $CDCl_3$, ppm].

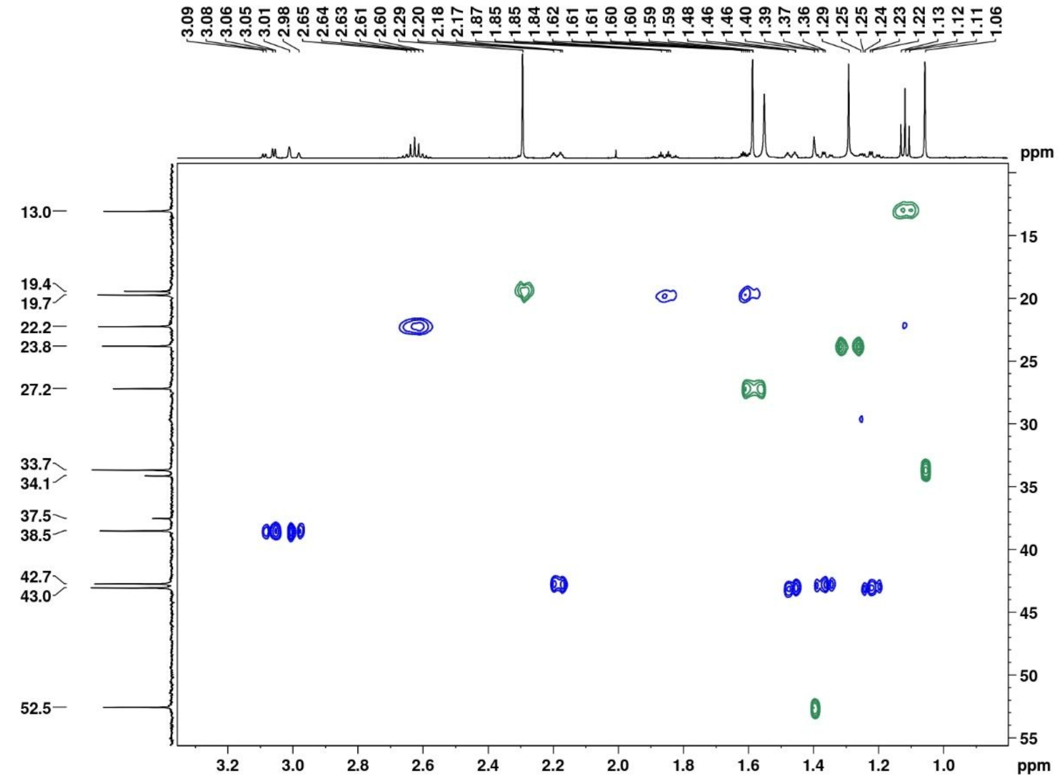

Supplementary Figure 10. HSQC NMR spectrum of Pyranthanol A **(1)** [600 MHz, $CDCl_3$, ppm].

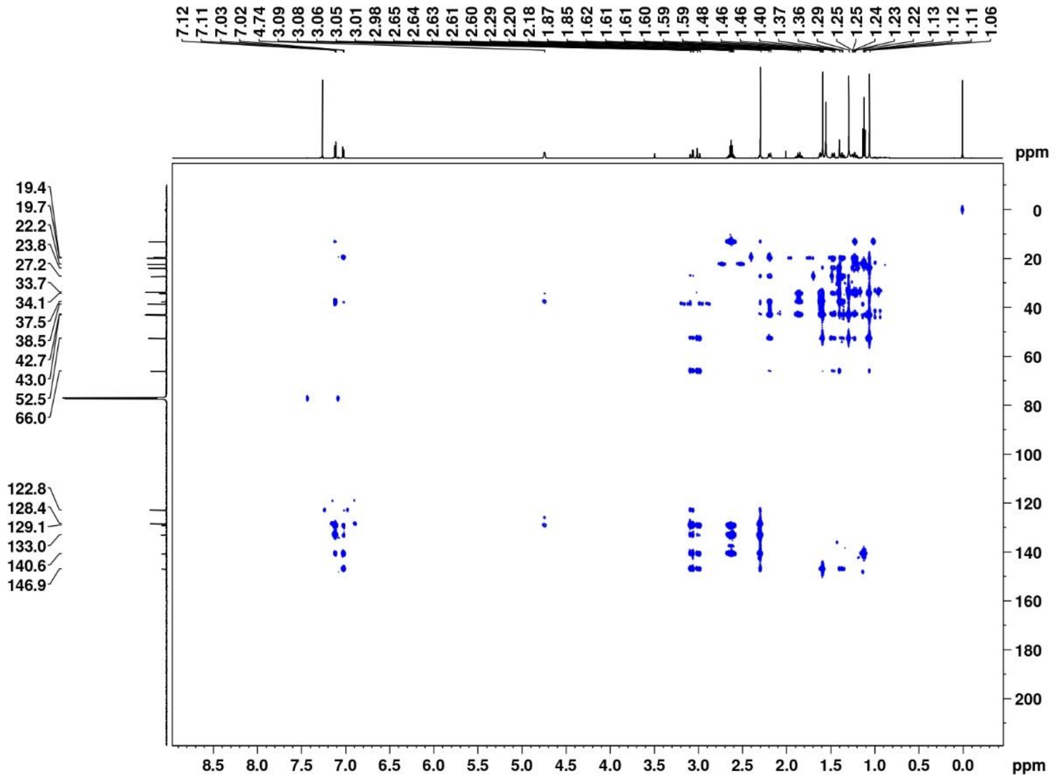

Supplementary Figure 11. HMBC NMR spectrum of Pyranthanol A **(1)** [600 MHz, $CDCl_3$, ppm].

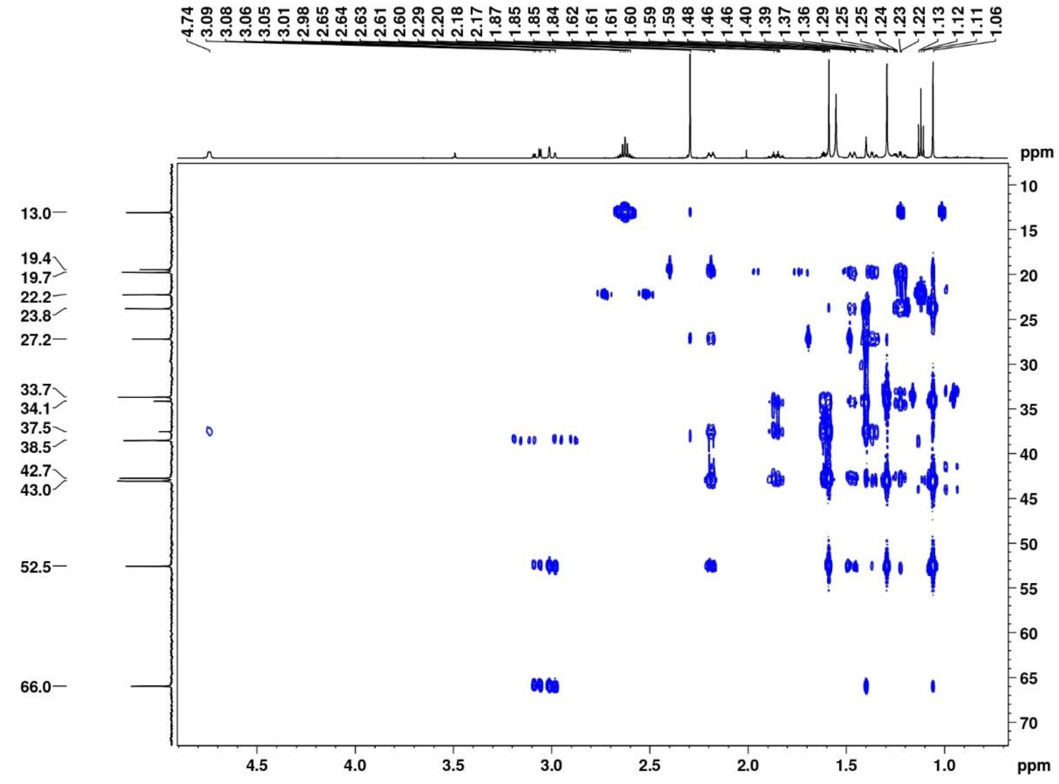

Supplementary Figure 12. HMBC NMR spectrum of Pyranthanol A **(1)** [600 MHz, $CDCl_3$, ppm].

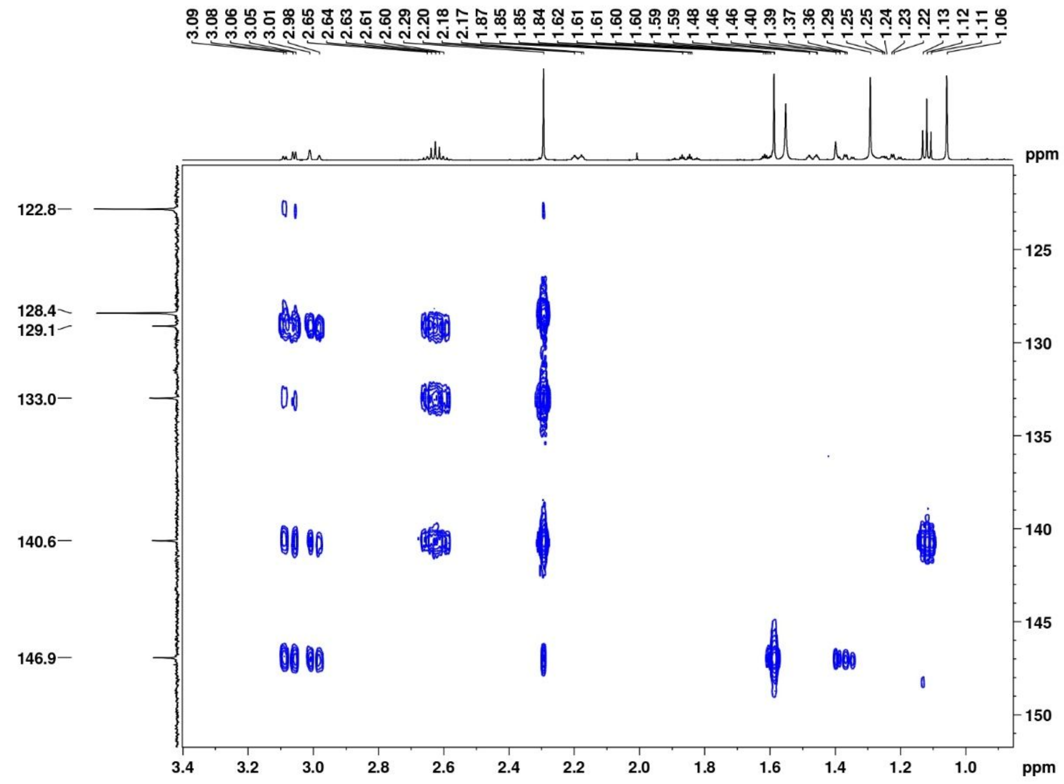

Supplementary Figure 13. HMBC NMR spectrum of Pyranthanol A **(1)** [600 MHz, $CDCl_3$, ppm].

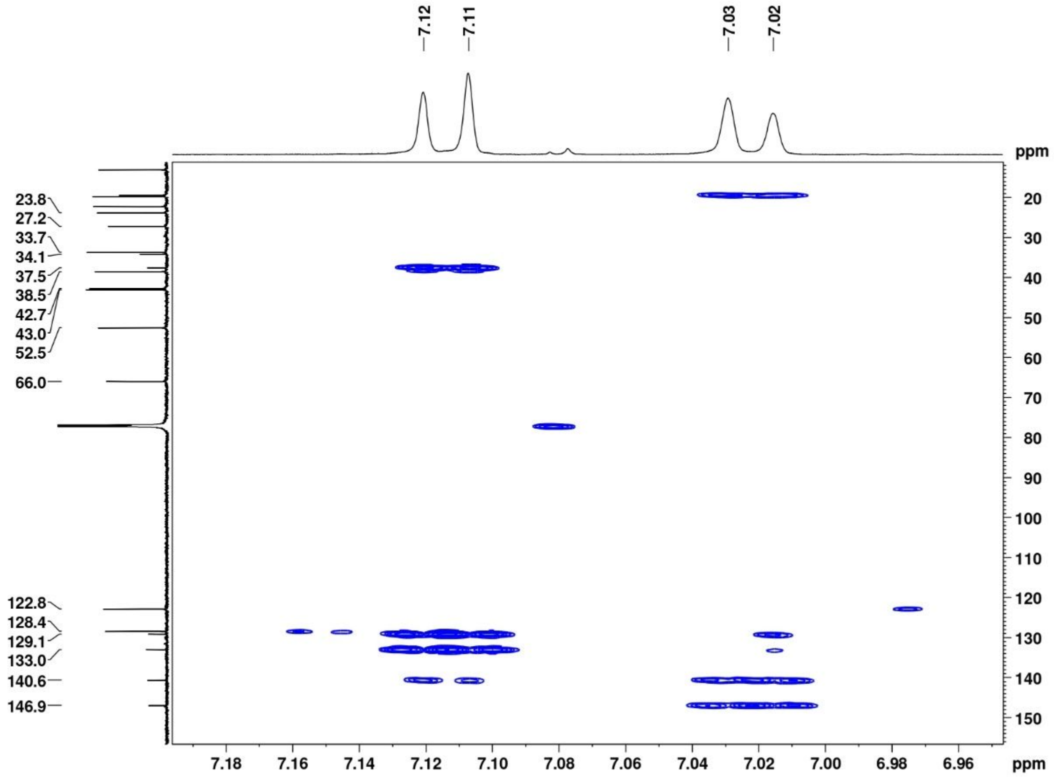

Supplementary Figure 14. HMBC NMR spectrum of Pyranthanol A **(1)** [600 MHz, $CDCl_3$, ppm].

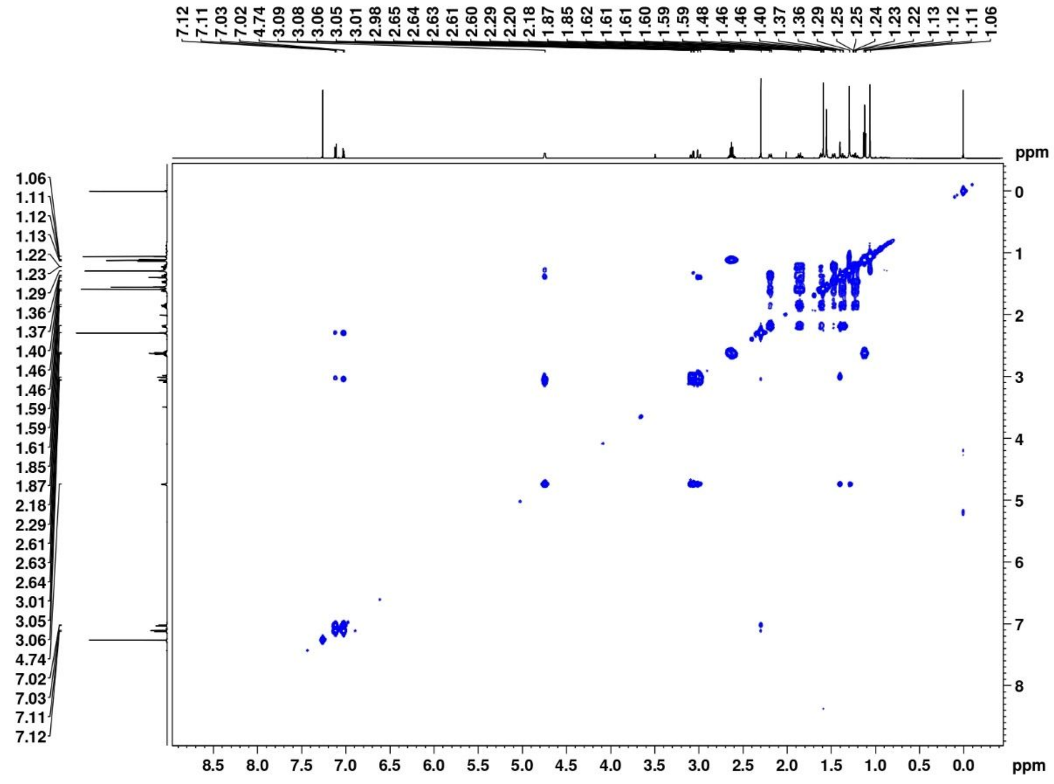

Supplementary Figure 15. COSY NMR spectrum of Pyranthanol A **(1)** [600 MHz, $CDCl_3$, ppm].

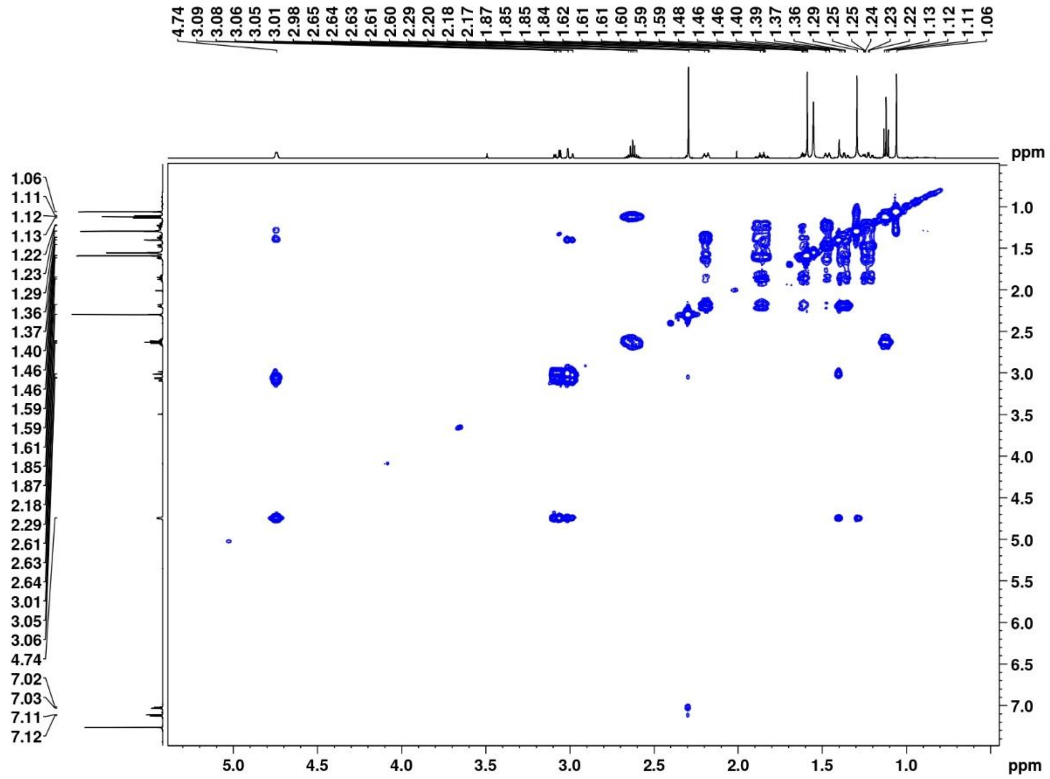

Supplementary Figure 16. COSY NMR spectrum of Pyranthanol A **(1)** [600 MHz, $CDCl_3$, ppm].

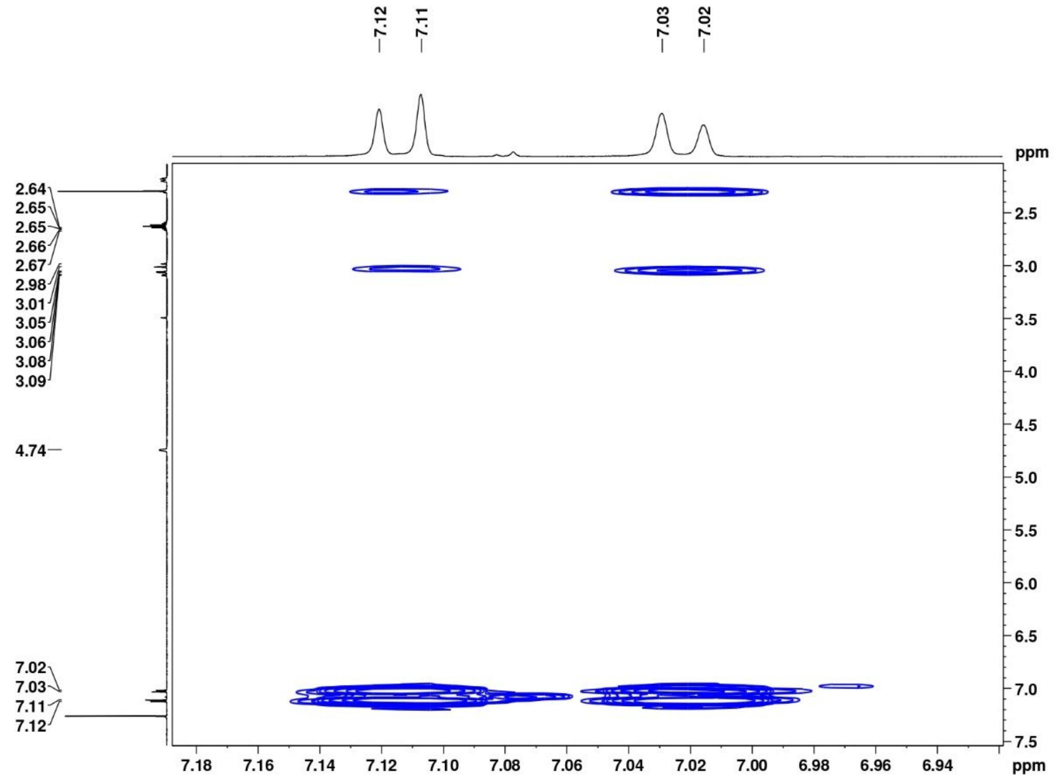

Supplementary Figure 17. COSY NMR spectrum of Pyranthanol A **(1)** [600 MHz, $CDCl_3$, ppm].

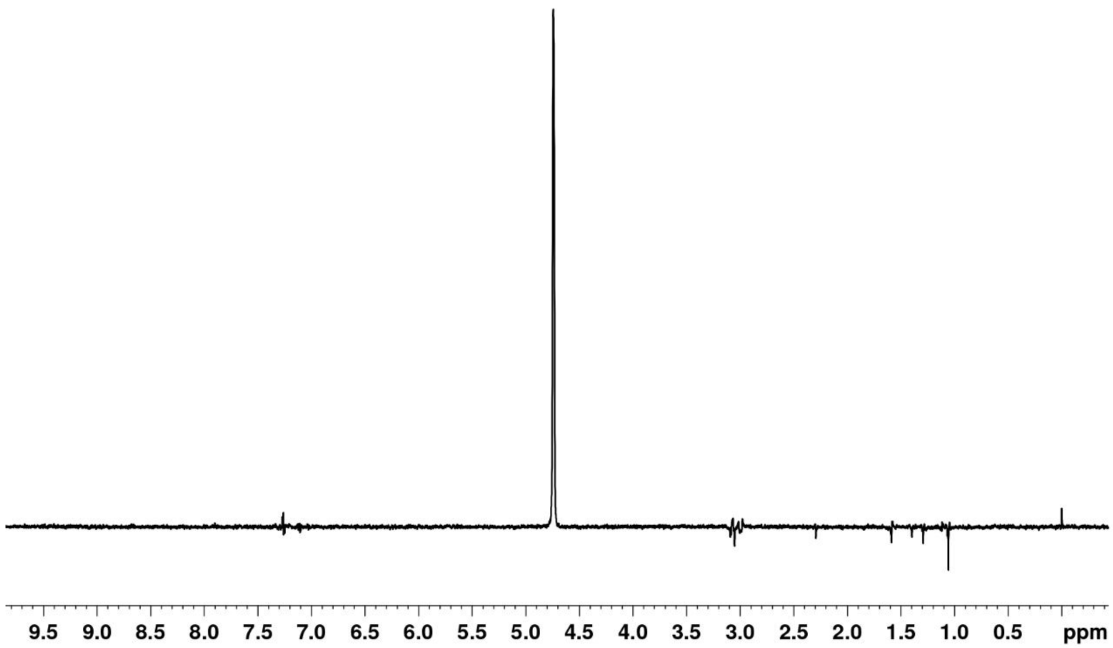

Supplementary Figure 18. gNoesy NMR spectrum of Pyranthanol A **(1)** [600 MHz, $CDCl_3$, ppm].

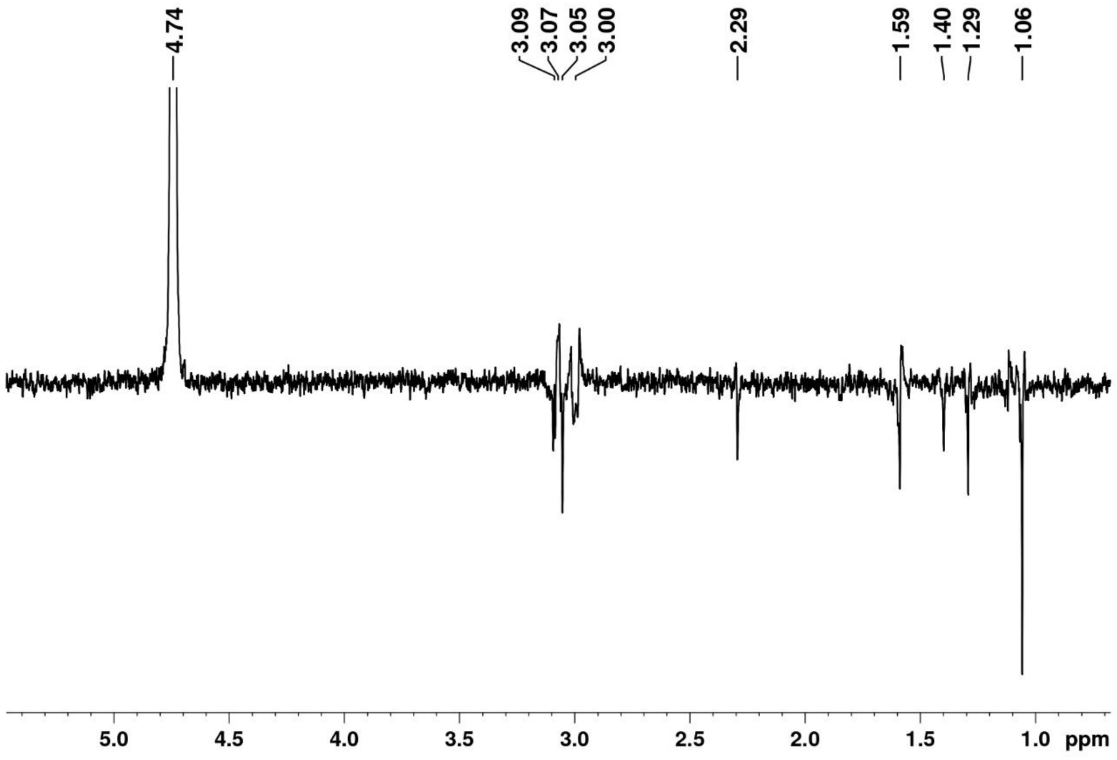

Supplementary Figure 19. gNoesy NMR spectrum of Pyranthanol A **(1)** [600 MHz, $CDCl_3$, ppm].

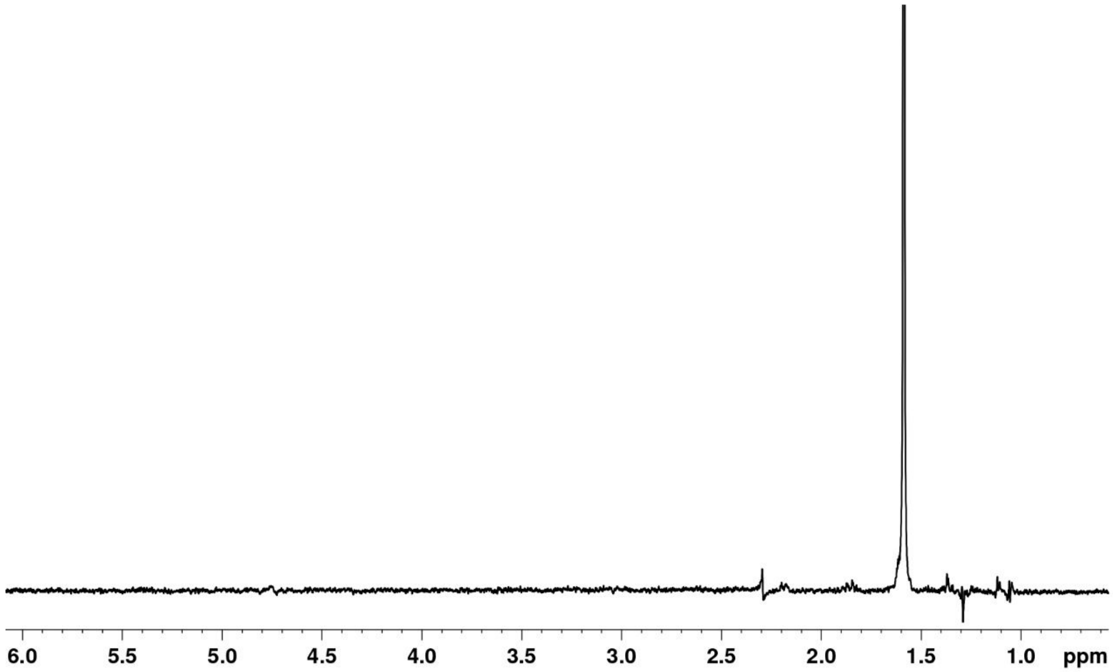

Supplementary Figure 20. gNoesy NMR spectrum of Pyranthanol A **(1)** [600 MHz, $CDCl_3$, ppm].

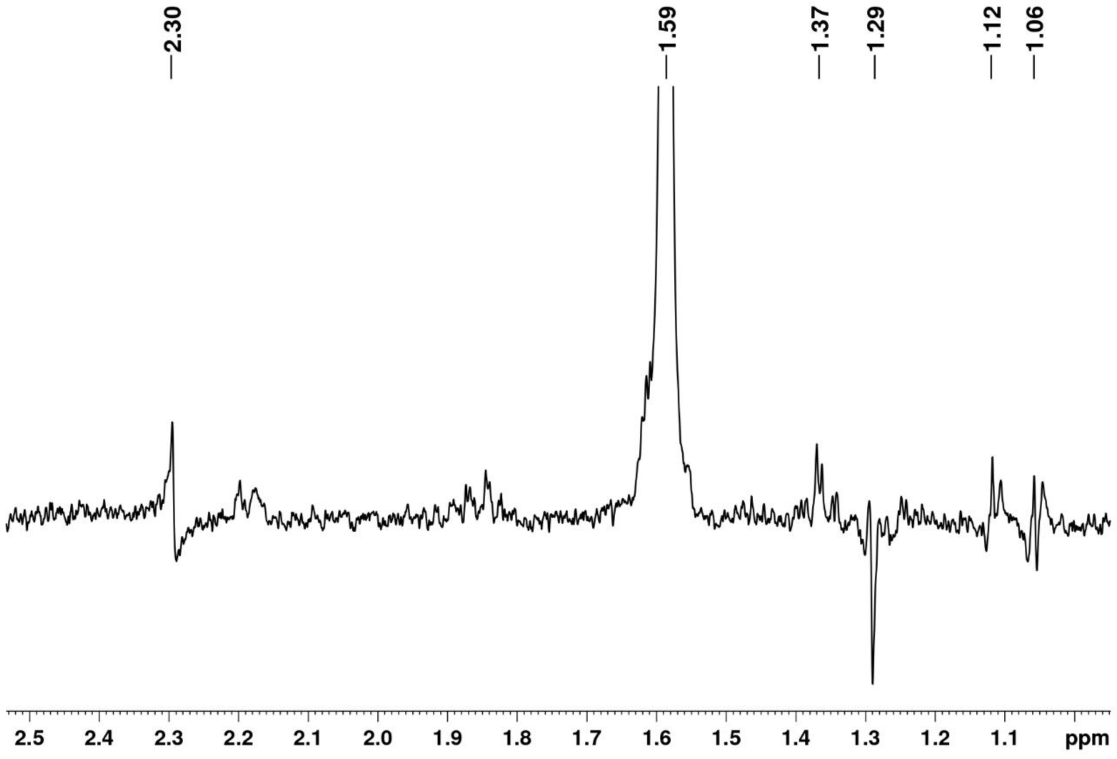

Supplementary Figure 21. gNoesy NMR spectrum of Pyranthanol A **(1)** [600 MHz, $CDCl_3$, ppm].

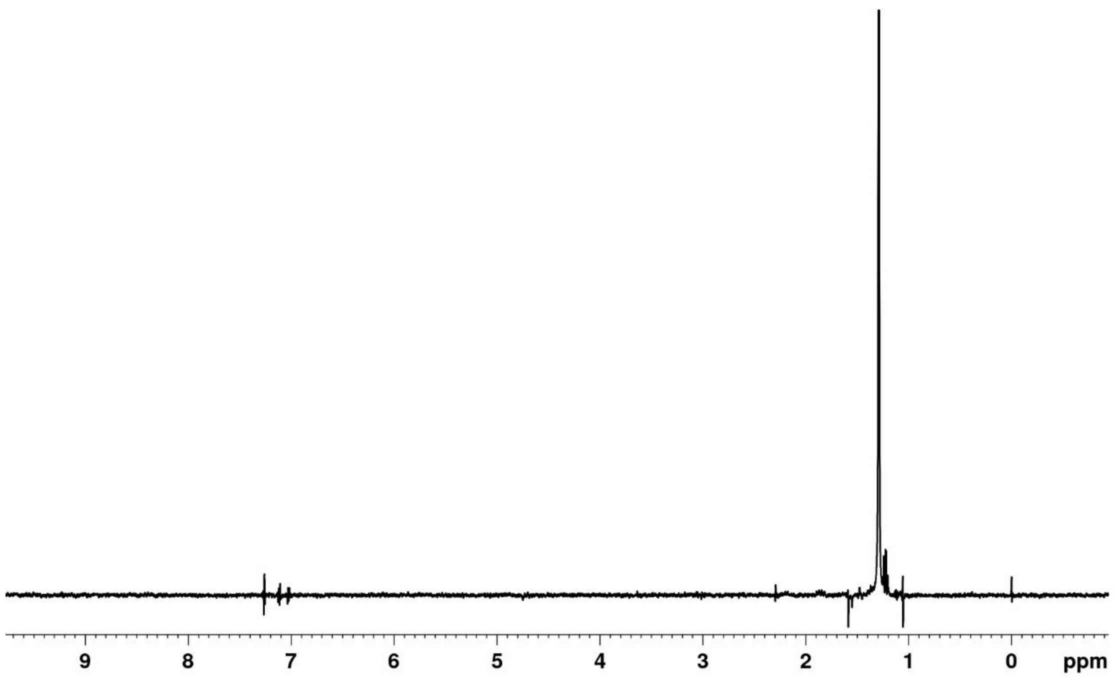

Supplementary Figure 22. gNoesy NMR spectrum of Pyranthanol A **(1)** [600 MHz, $CDCl_3$, ppm].

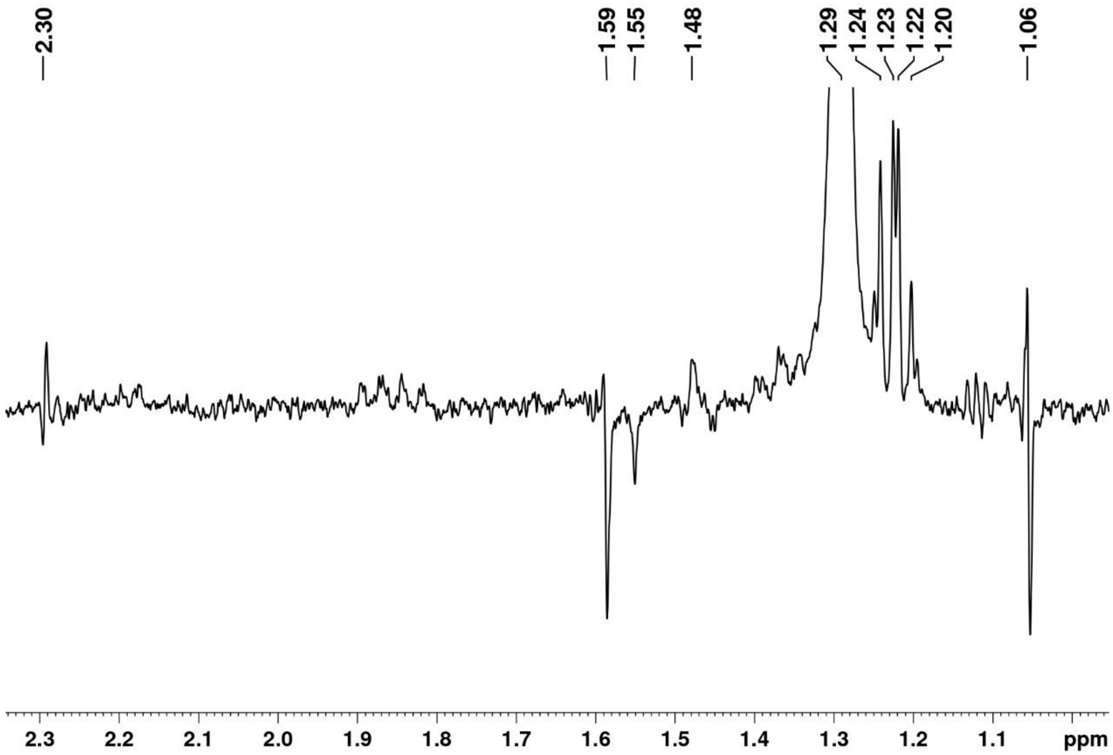

Supplementary Figure 23. gNoesy NMR spectrum of Pyranthanol A **(1)** [600 MHz, $CDCl_3$, ppm].

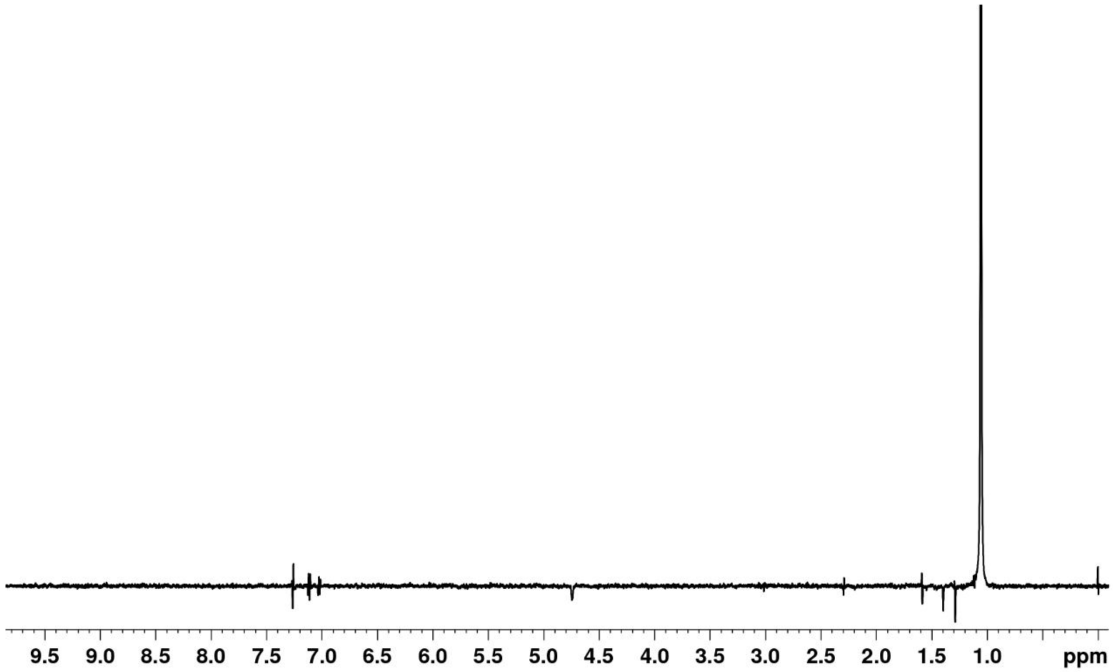

Supplementary Figure 24. gNoesy NMR spectrum of Pyranthanol A **(1)** [600 MHz, $CDCl_3$, ppm].

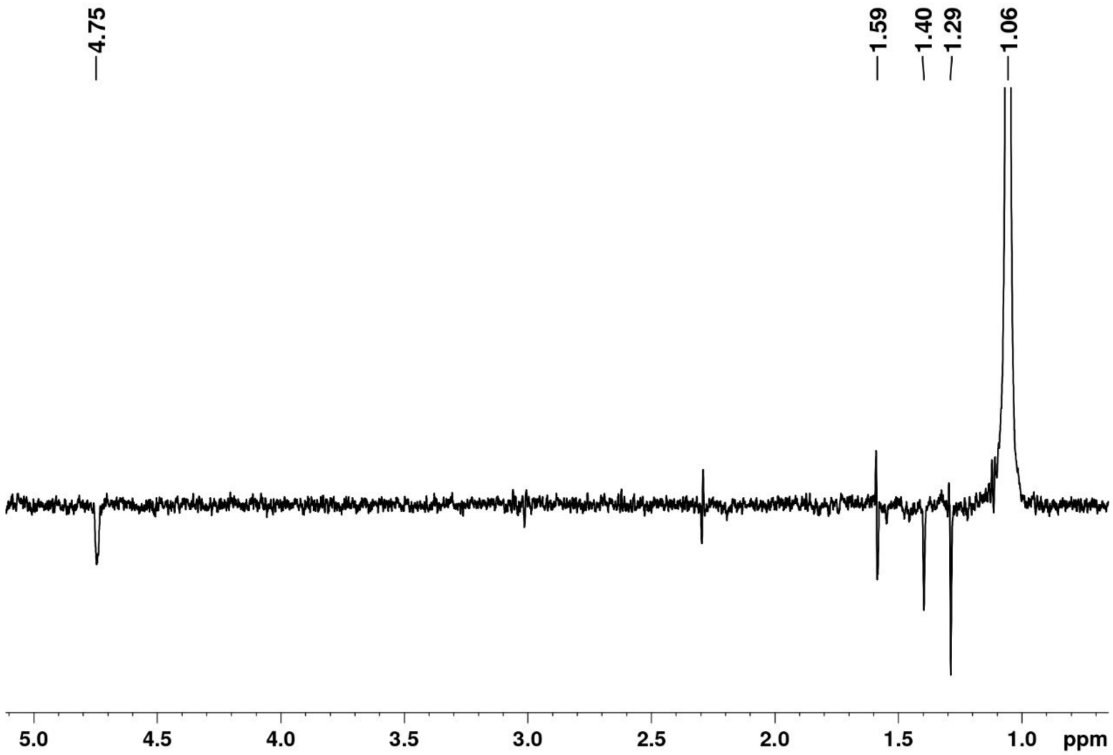

Supplementary Figure 25. gNoesy NMR spectrum of Pyranthanol A **(1)** [600 MHz, $CDCl_3$, ppm].

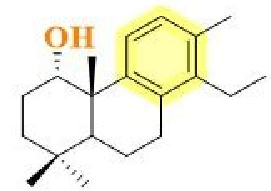

2

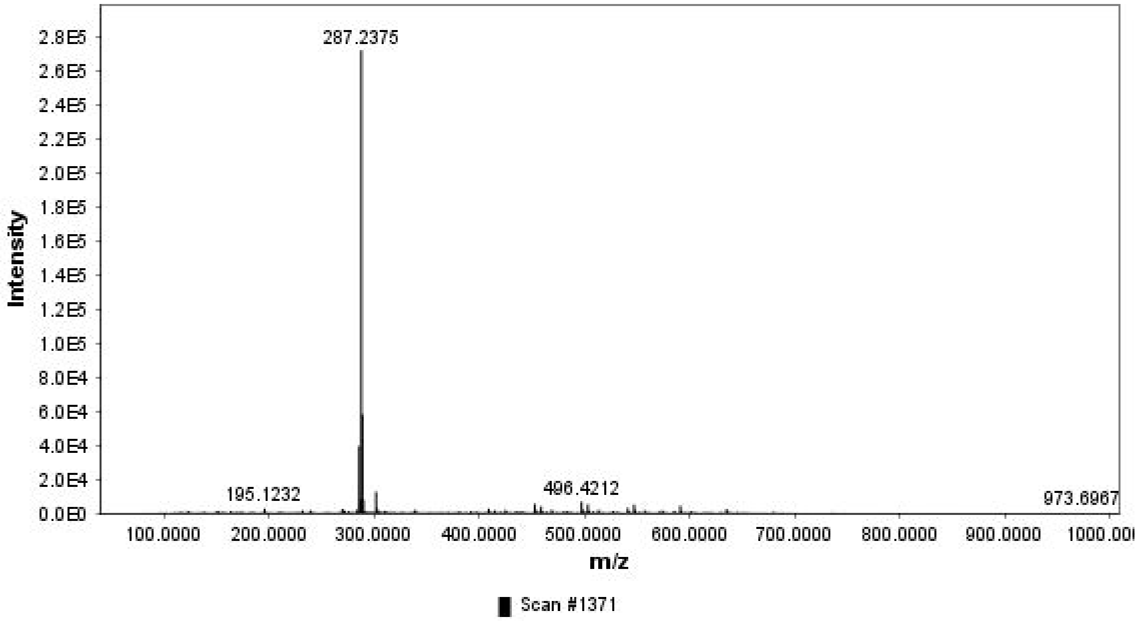

Supplementary Figure 26. High resolution mass spectrum of Pyranthanol B **(2)**.

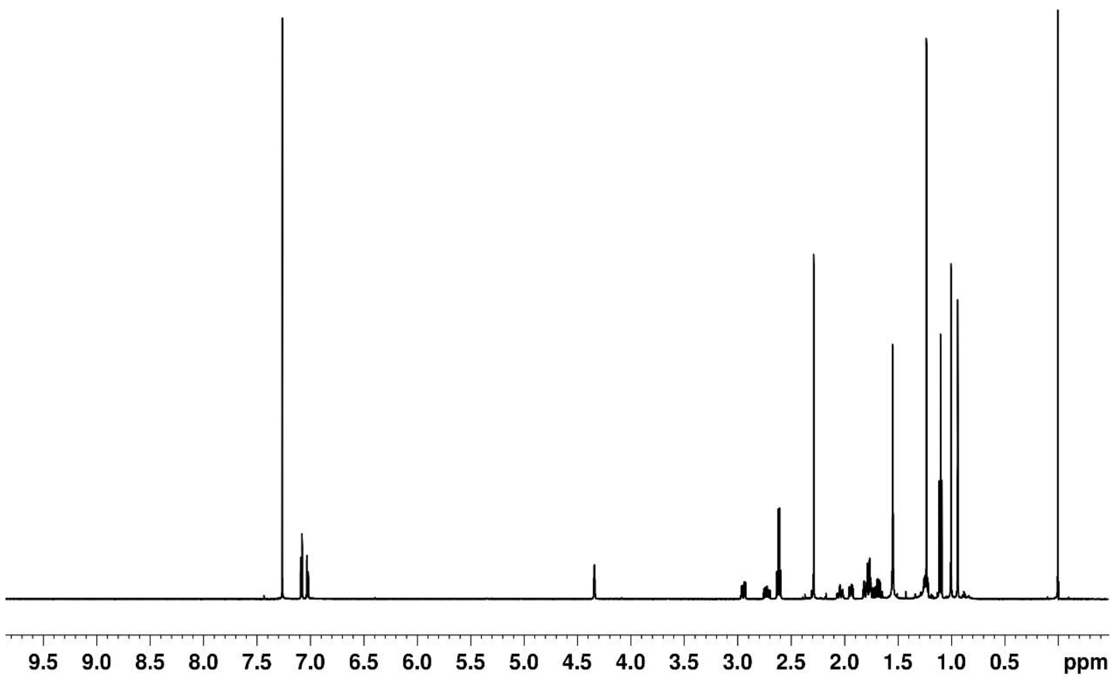

Supplementary Figure 27. ^{1}H NMR spectrum of Pyranthanol B **(2)** [600 MHz, $CDCl_3$, ppm].

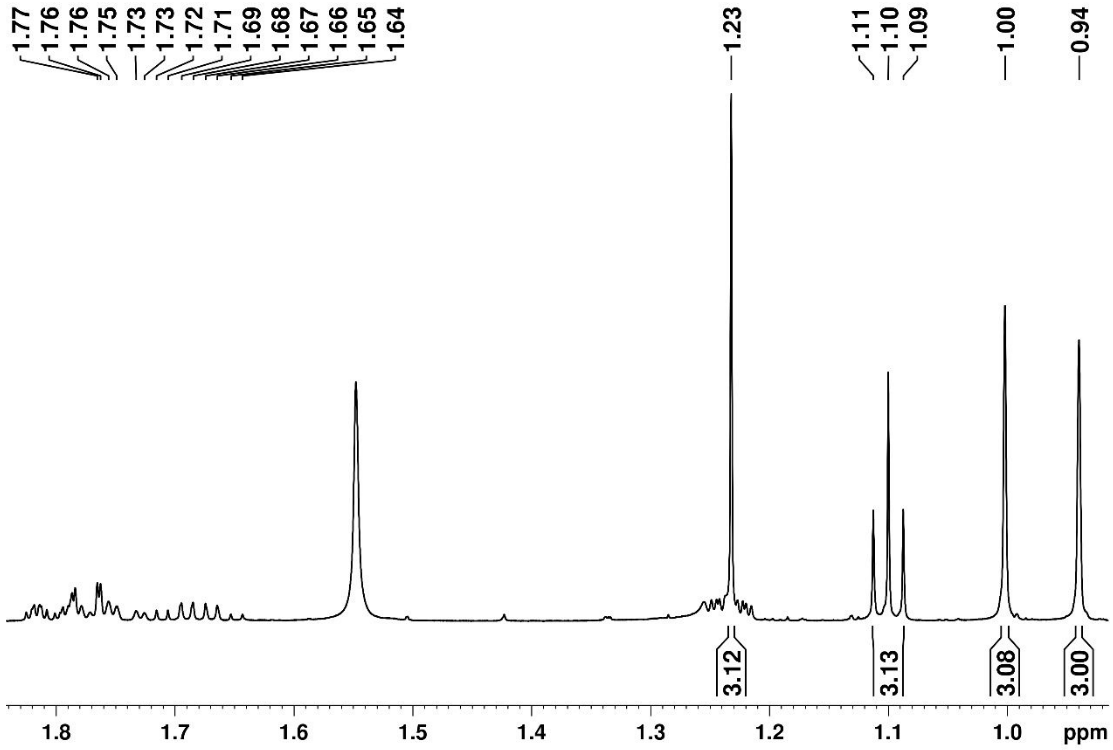

Supplementary Figure 28. ^{1}H NMR spectrum of Pyranthanol B **(2)** [600 MHz, $CDCl_3$, ppm].

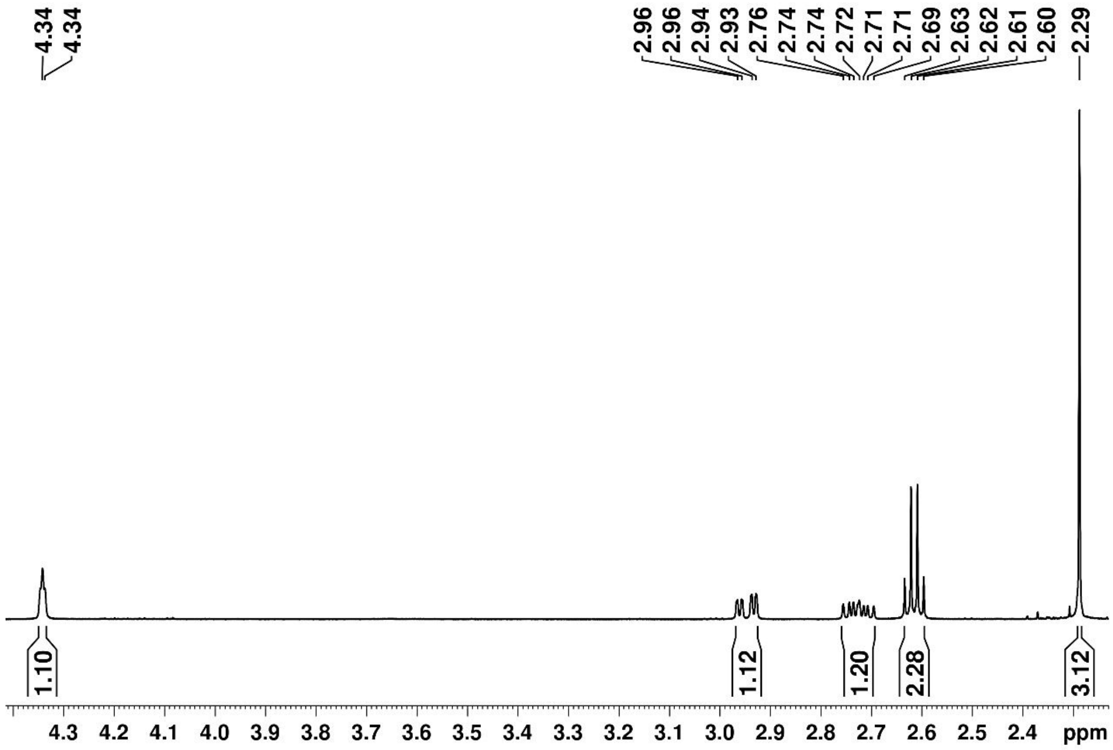

Supplementary Figure 29. ^{1}H NMR spectrum of Pyranthanol B **(2)** [600 MHz, $CDCl_3$, ppm].

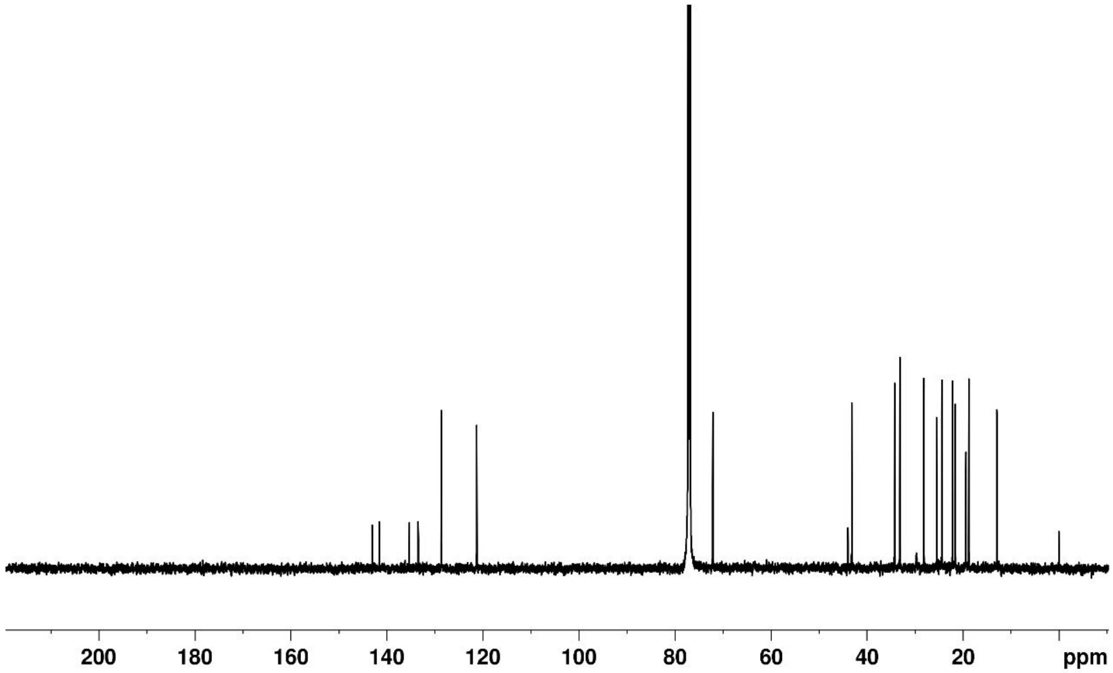

Supplementary Figure 30. ^{13}C NMR spectrum of Pyranthanol B **(2)** [150 MHz, $CDCl_3$, ppm].

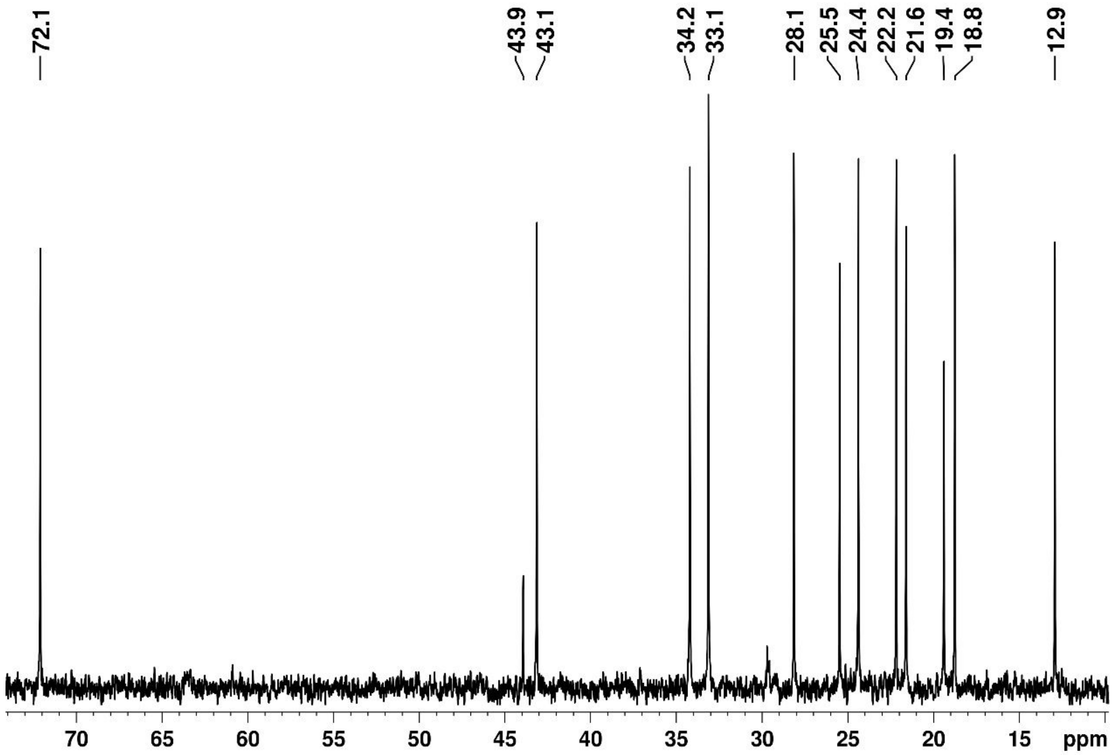

Supplementary Figure 31. ^{13}C NMR spectrum of Pyranthanol B **(2)** [150 MHz, $CDCl_3$, ppm].

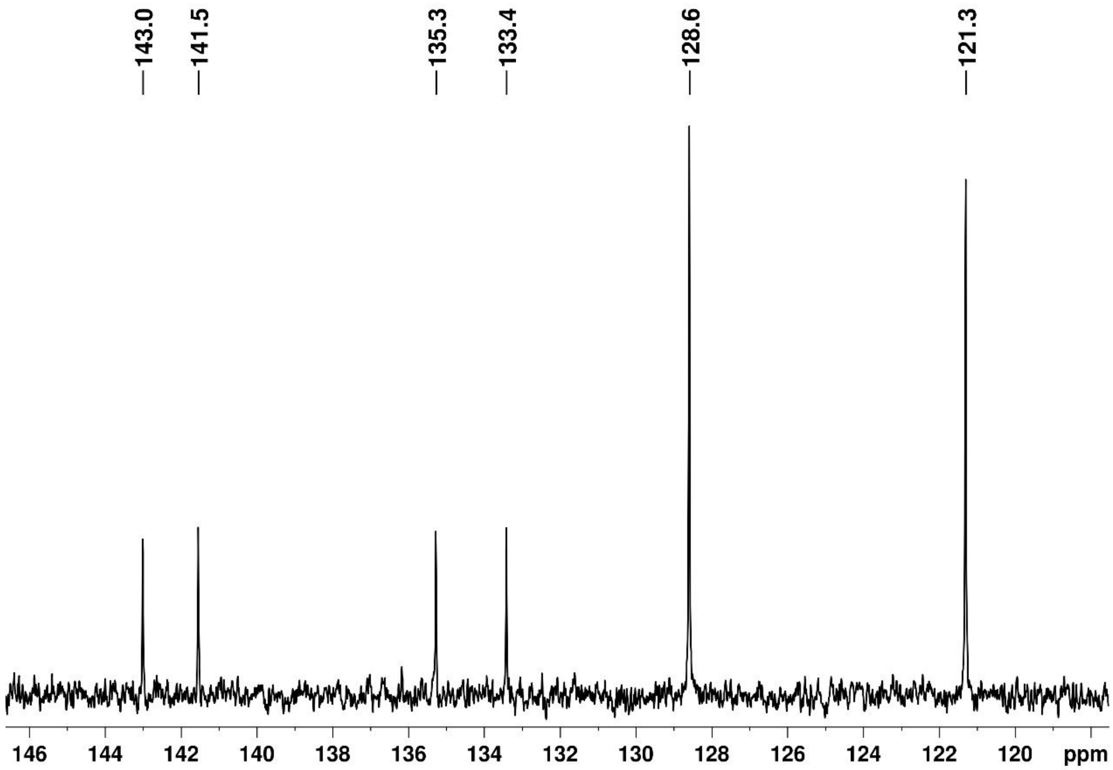

Supplementary Figure 32. ^{13}C NMR spectrum of Pyranthanol B **(2)** [150 MHz, $CDCl_3$, ppm].

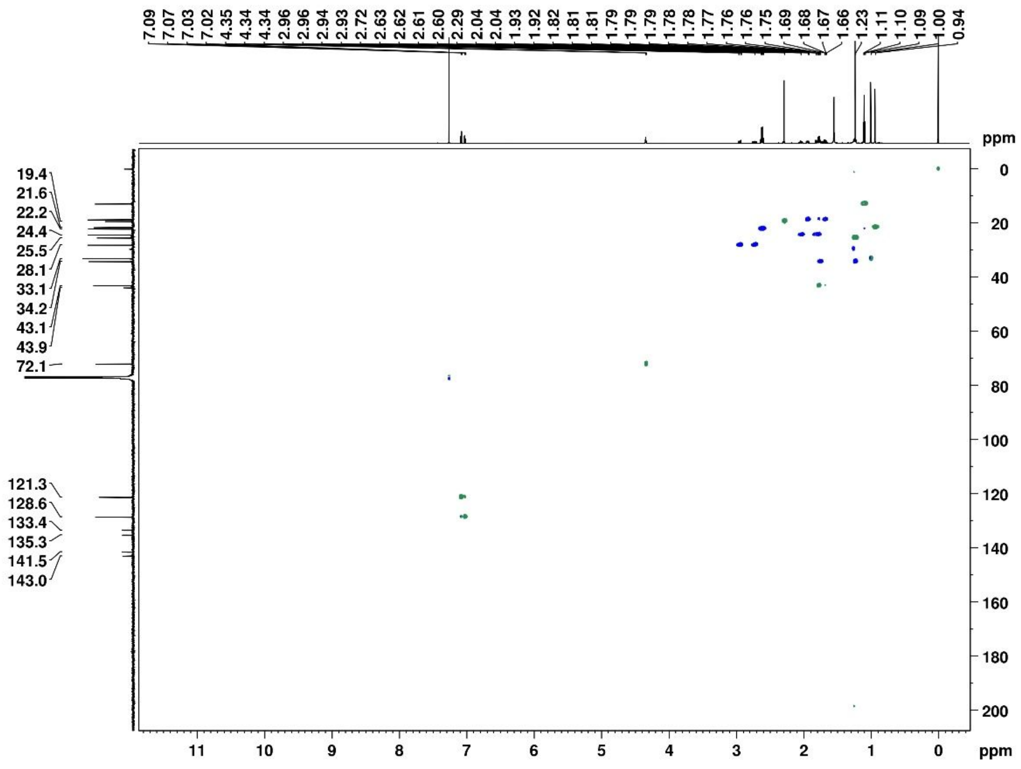

Supplementary Figure 33. HSQC NMR spectrum of Pyranthanol B **(2)** [600 MHz, $CDCl_3$, ppm].

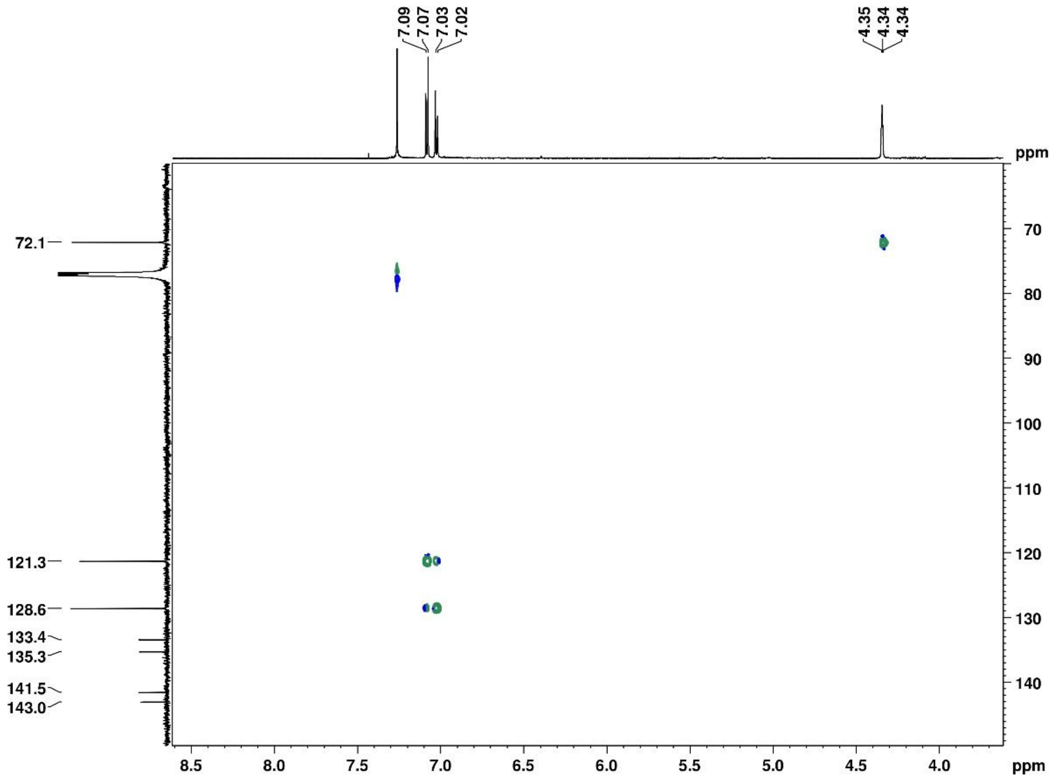

Supplementary Figure 34. HSQC NMR spectrum of Pyranthanol B **(2)** [600 MHz, $CDCl_3$, ppm].

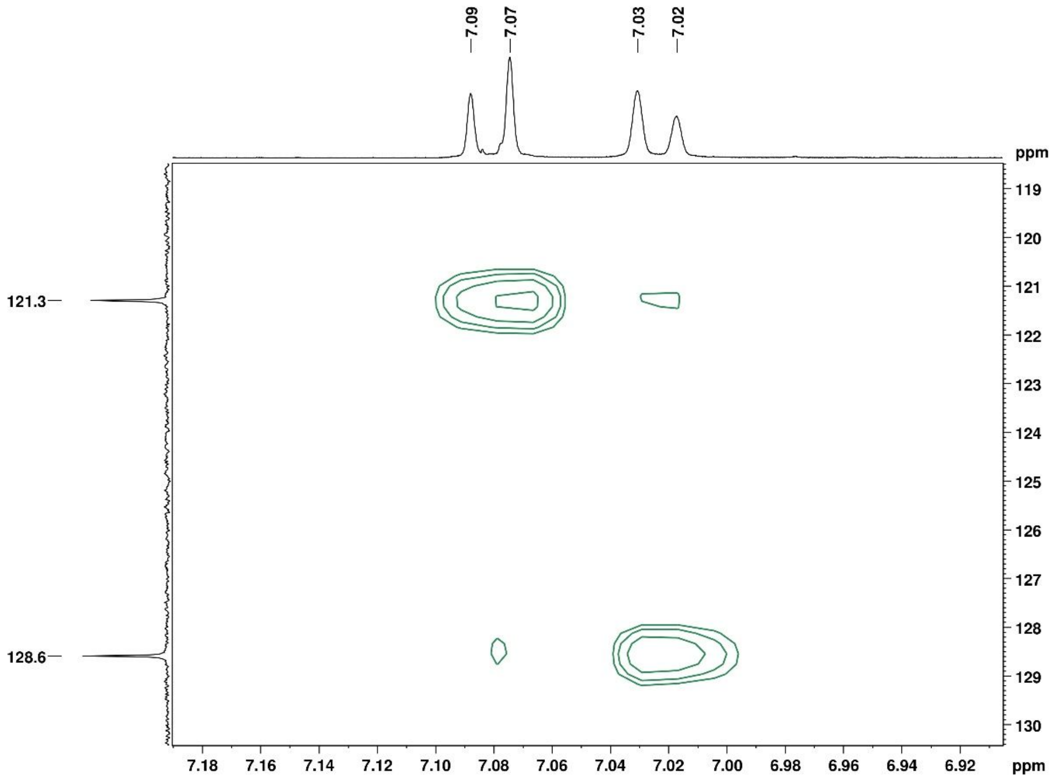

Supplementary Figure 35. HSQC NMR spectrum of Pyranthanol B **(2)** [600 MHz, $CDCl_3$, ppm].

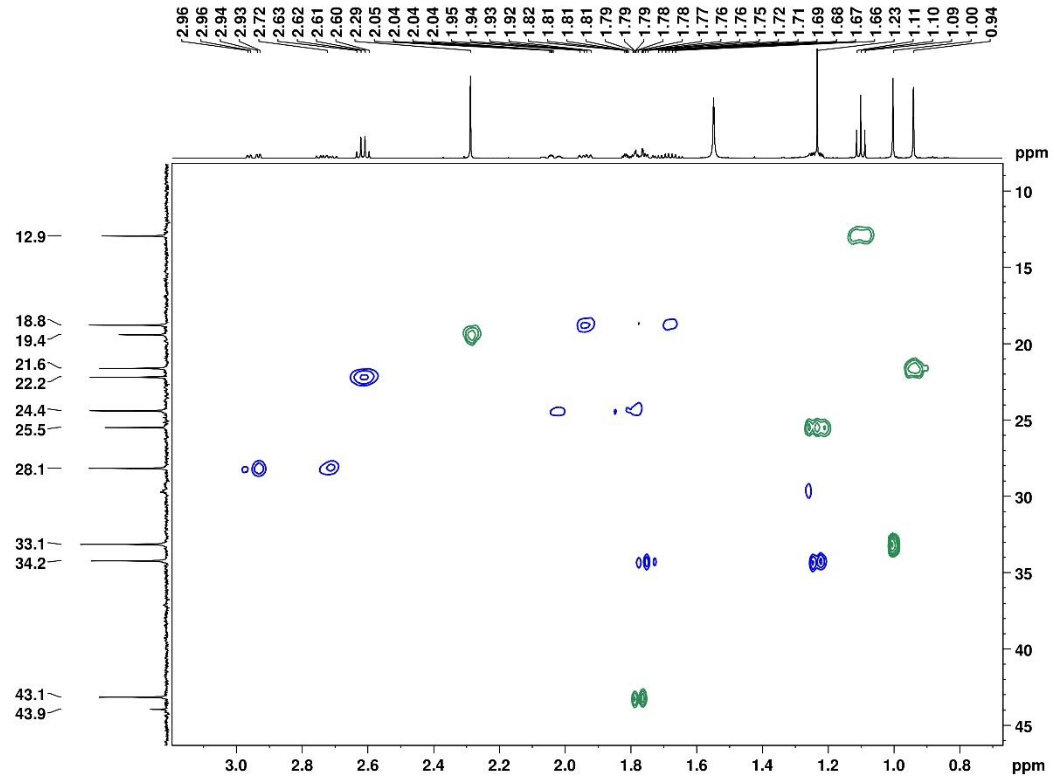

Supplementary Figure 36. HSQC NMR spectrum of Pyranthanol B **(2)** [600 MHz, $CDCl_3$, ppm].

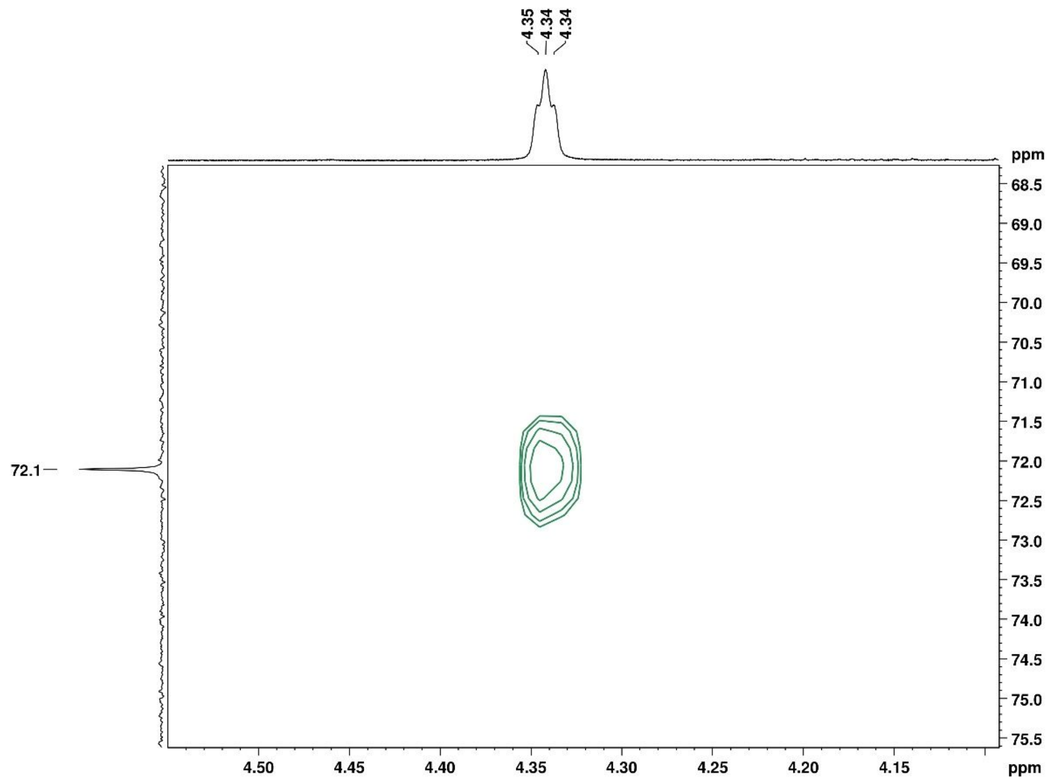

Supplementary Figure 37. HSQC NMR spectrum of Pyranthanol B **(2)** [600 MHz, $CDCl_3$, ppm].

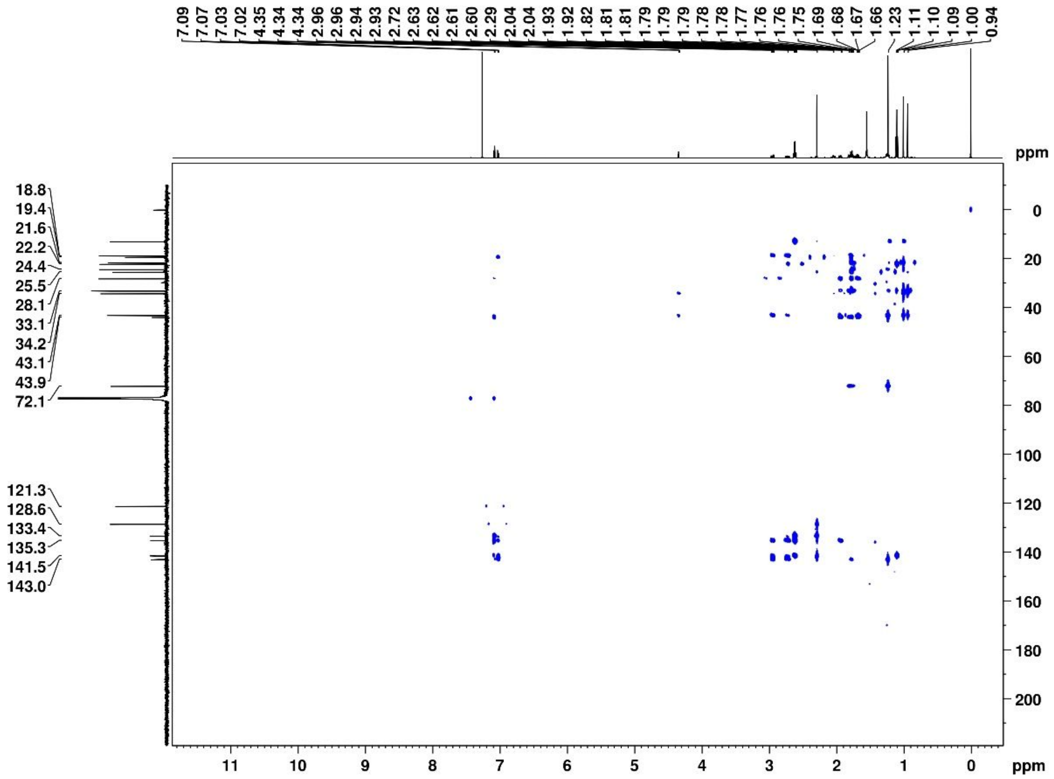

Supplementary Figure 38. HMBC NMR spectrum of Pyranthanol B **(2)** [600 MHz, $CDCl_3$, ppm].

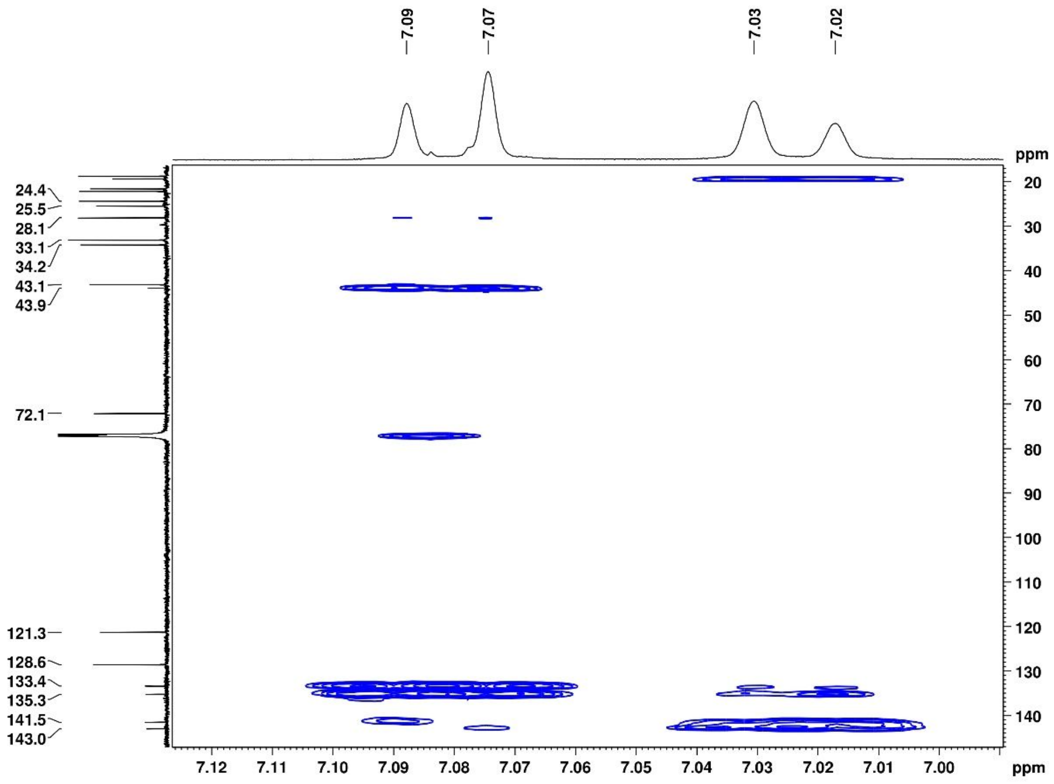

Supplementary Figure 39. HMBC NMR spectrum of Pyranthanol B **(2)** [600 MHz, $CDCl_3$, ppm].

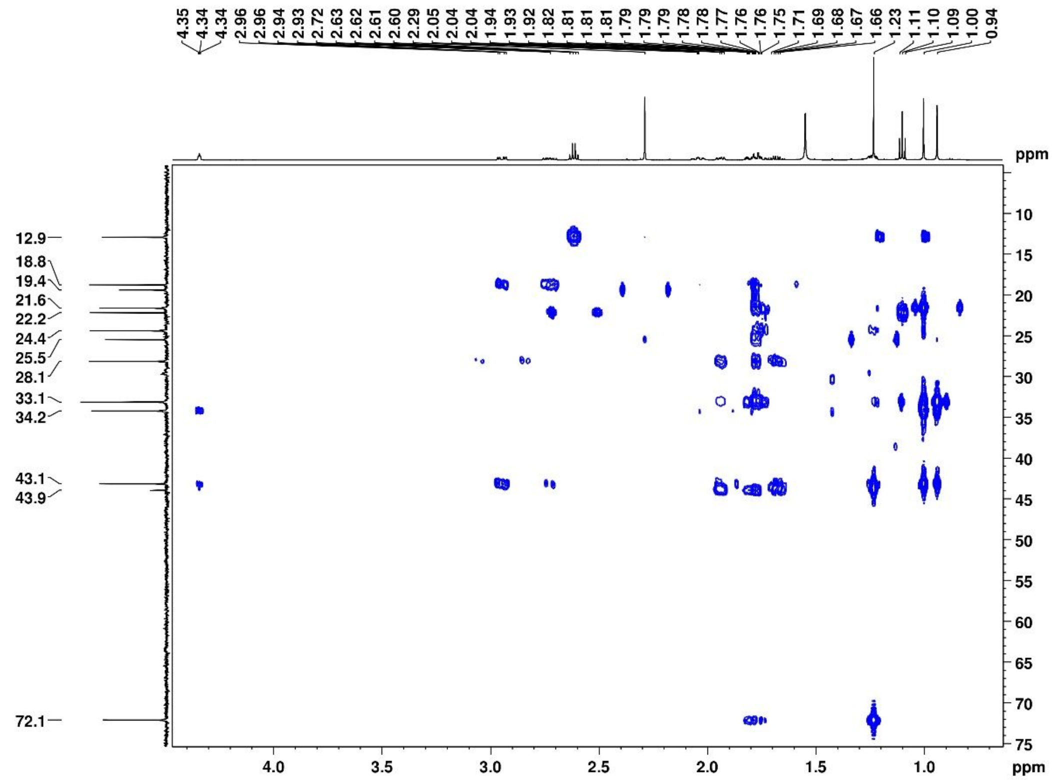

Supplementary Figure 40. HMBC NMR spectrum of Pyranthanol B **(2)** [600 MHz, $CDCl_3$, ppm]

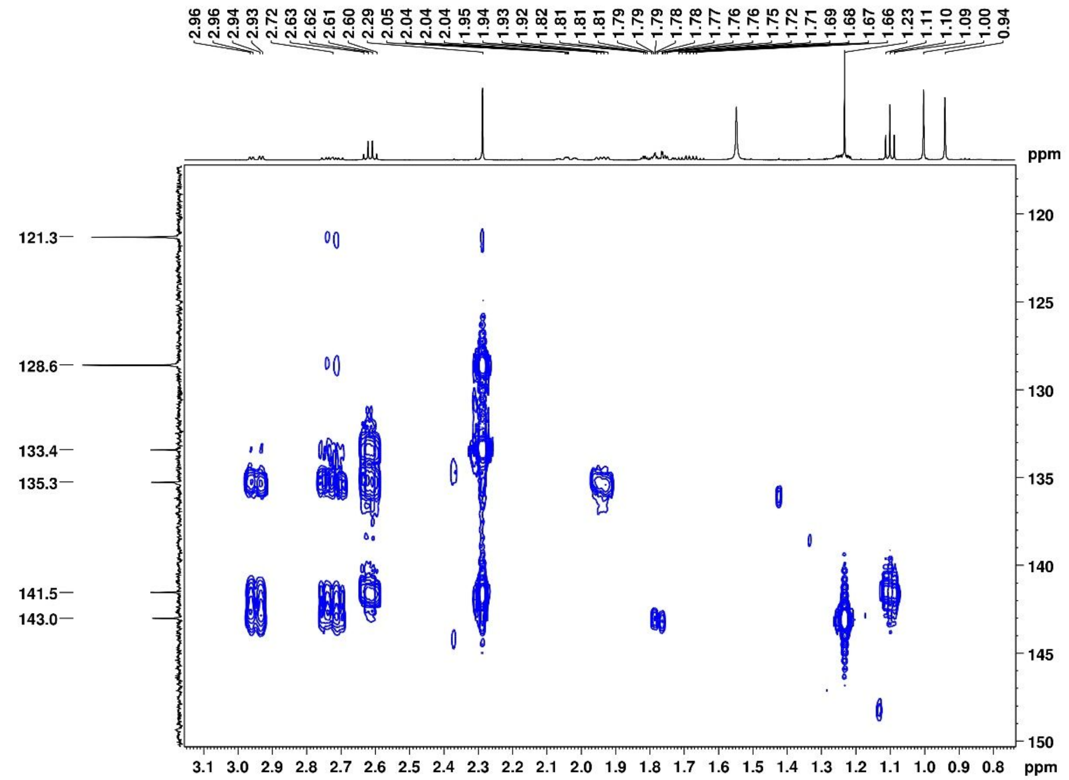

Supplementary Figure 41. HMBC NMR spectrum of Pyranthanol B **(2)** [600 MHz, $CDCl_3$, ppm].

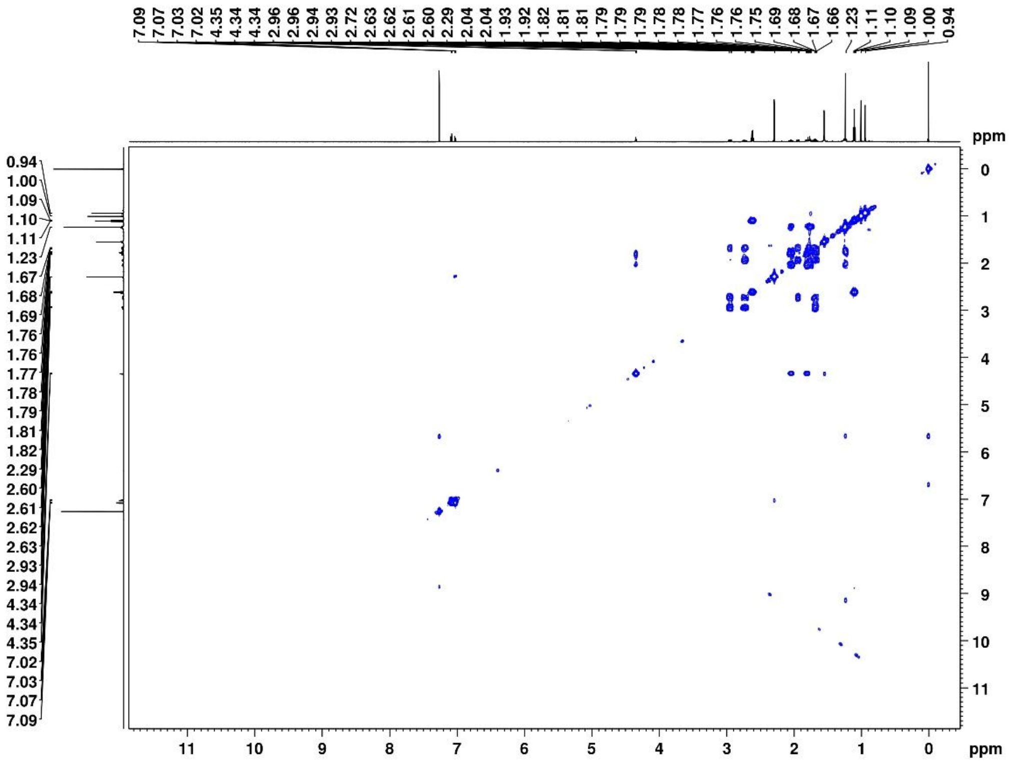

Supplementary Figure 42. COSY NMR spectrum of Pyranthanol B **(2)** [600 MHz, $CDCl_3$, ppm].

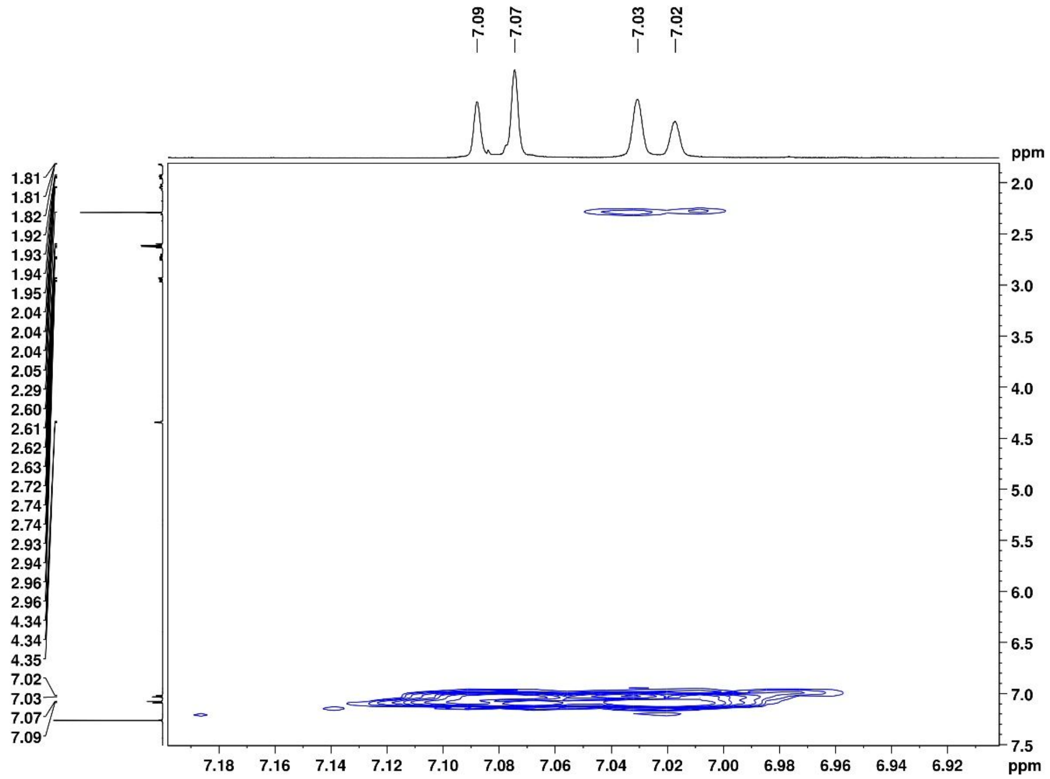

Supplementary Figure 43. COSY NMR spectrum of Pyranthanol B **(2)** [600 MHz, $CDCl_3$, ppm].

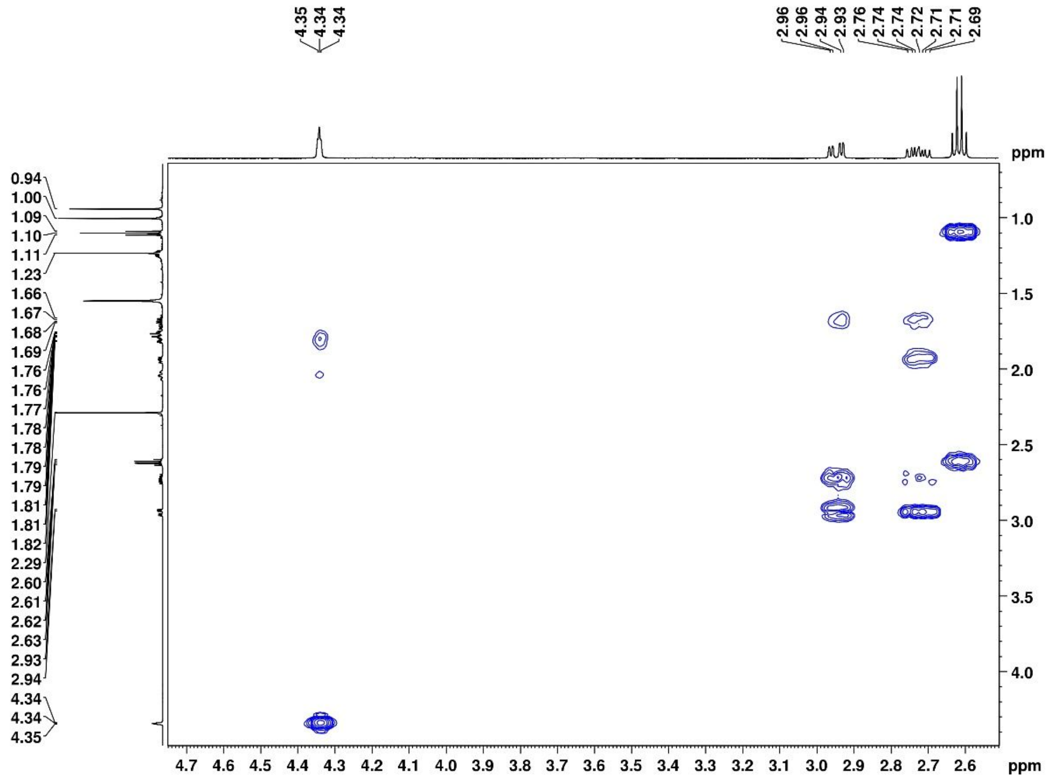

Supplementary Figure 44. COSY NMR spectrum of Pyranthanol B **(2)** [600 MHz, $CDCl_3$, ppm].

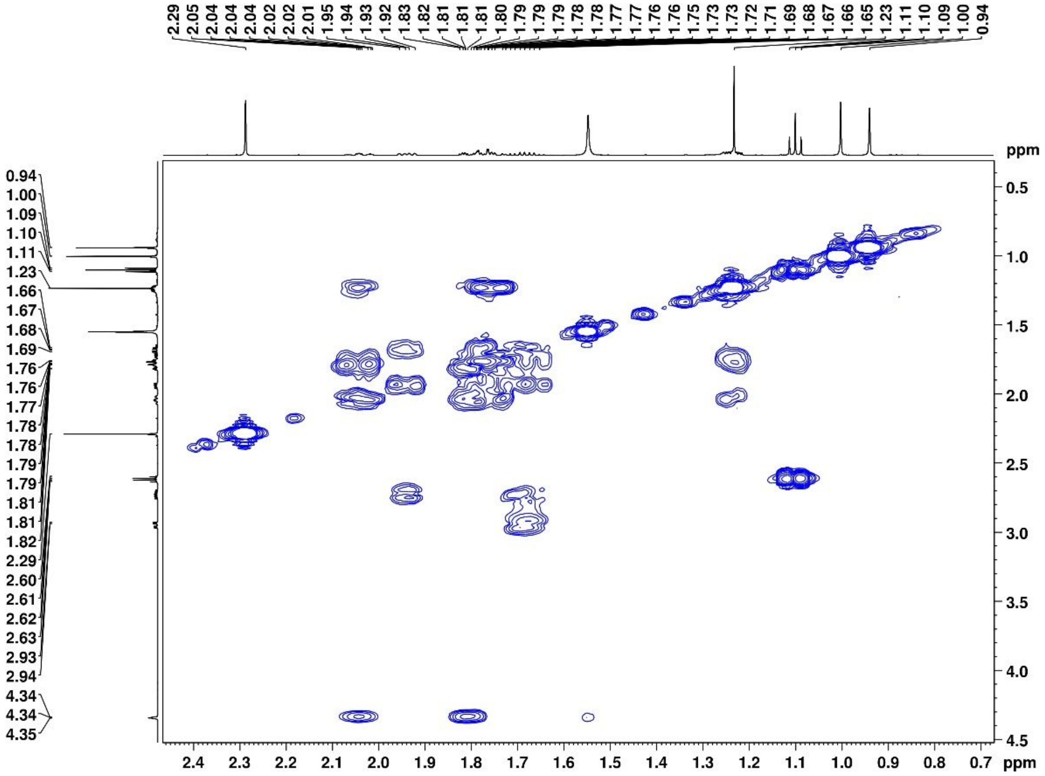

Supplementary Figure 45. COSY NMR spectrum of Pyranthanol B **(2)** [600 MHz, $CDCl_3$, ppm].

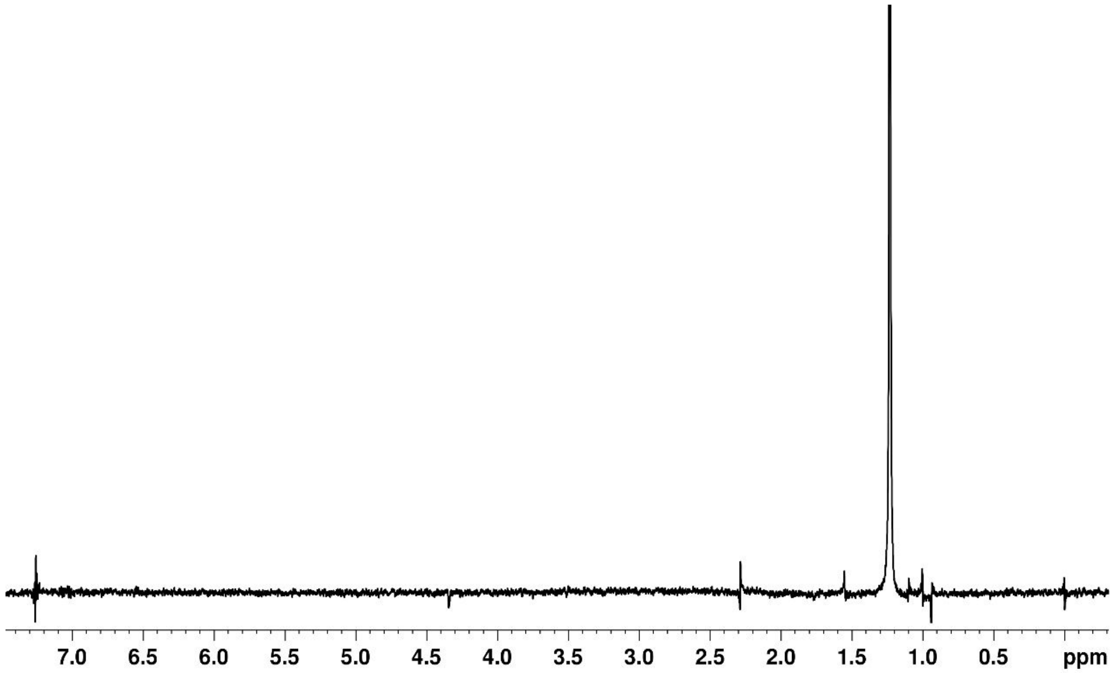

Supplementary Figure 46. gNoesy NMR spectrum of Pyranthanol B **(2)** [600 MHz, $CDCl_3$, ppm].

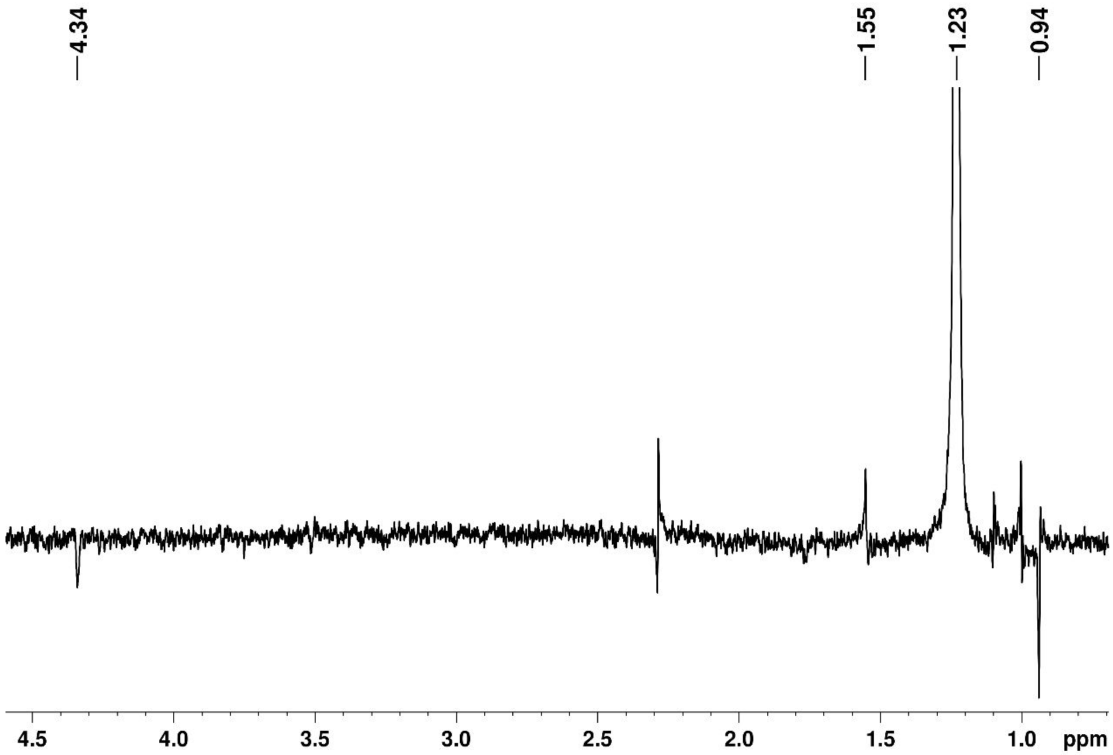

Supplementary Figure 47. gNoesy NMR spectrum of Pyranthanol B **(2)** [600 MHz, $CDCl_3$, ppm].

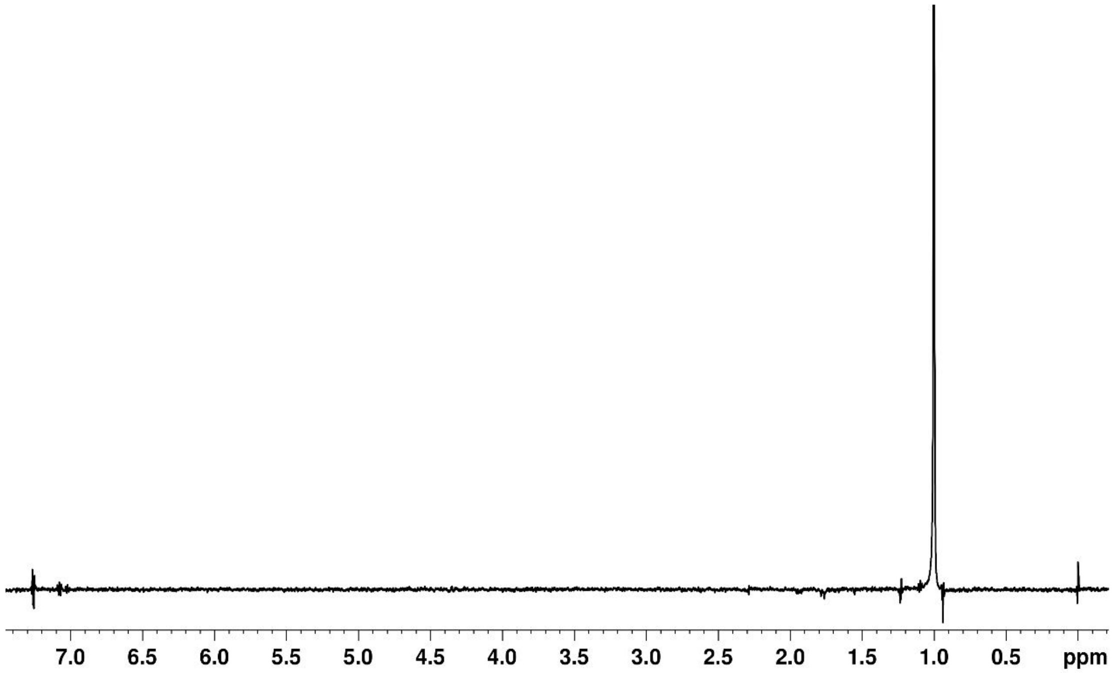

Supplementary Figure 48. gNoesy NMR spectrum of Pyranthanol B **(2)** [600 MHz, $CDCl_3$, ppm].

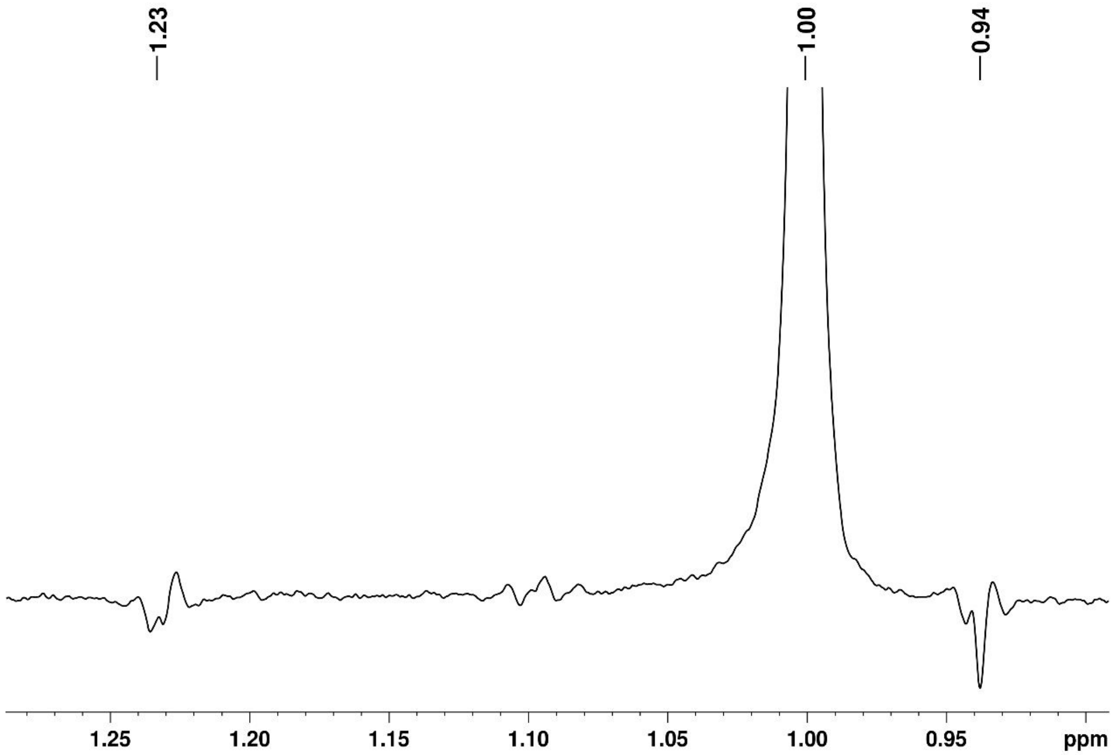

Supplementary Figure 49. gNoesy NMR spectrum of Pyranthanol B **(2)** [600 MHz, $CDCl_3$, ppm].

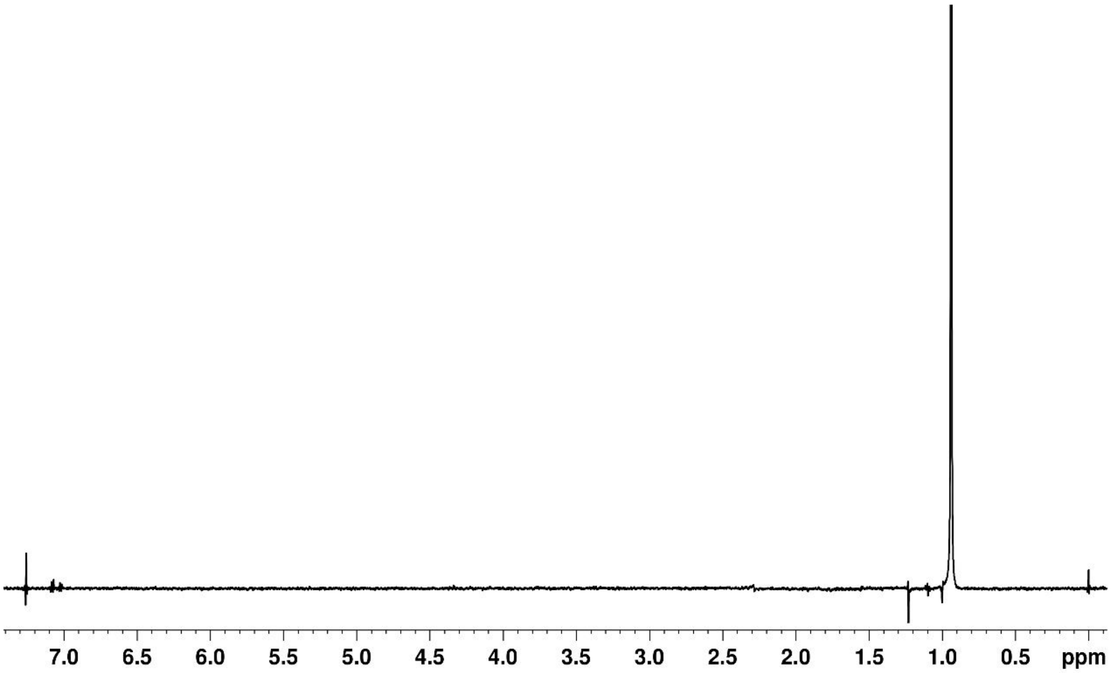

Supplementary Figure 50. gNoesy NMR spectrum of Pyranthanol B **(2)** [600 MHz, $CDCl_3$, ppm].

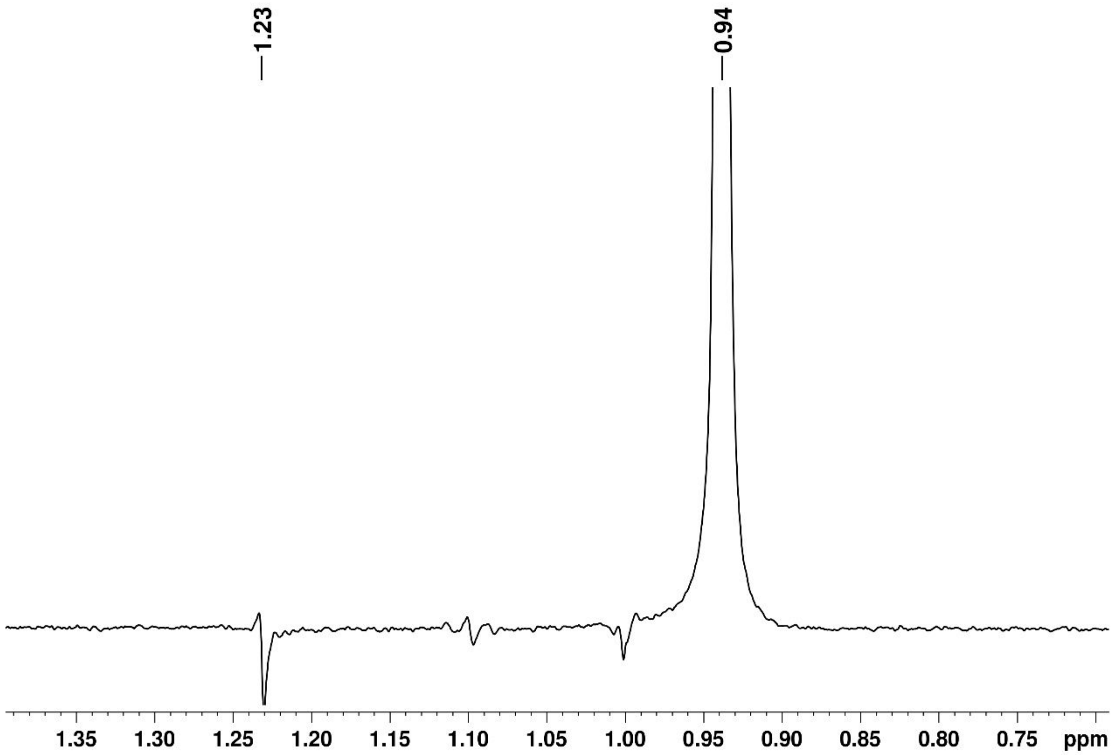

Supplementary Figure 51. gNoesy NMR spectrum of Pyranthanol B **(2)** [600 MHz, $CDCl_3$, ppm].

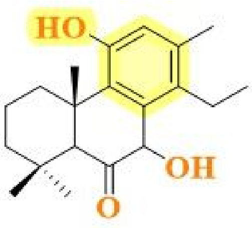

3

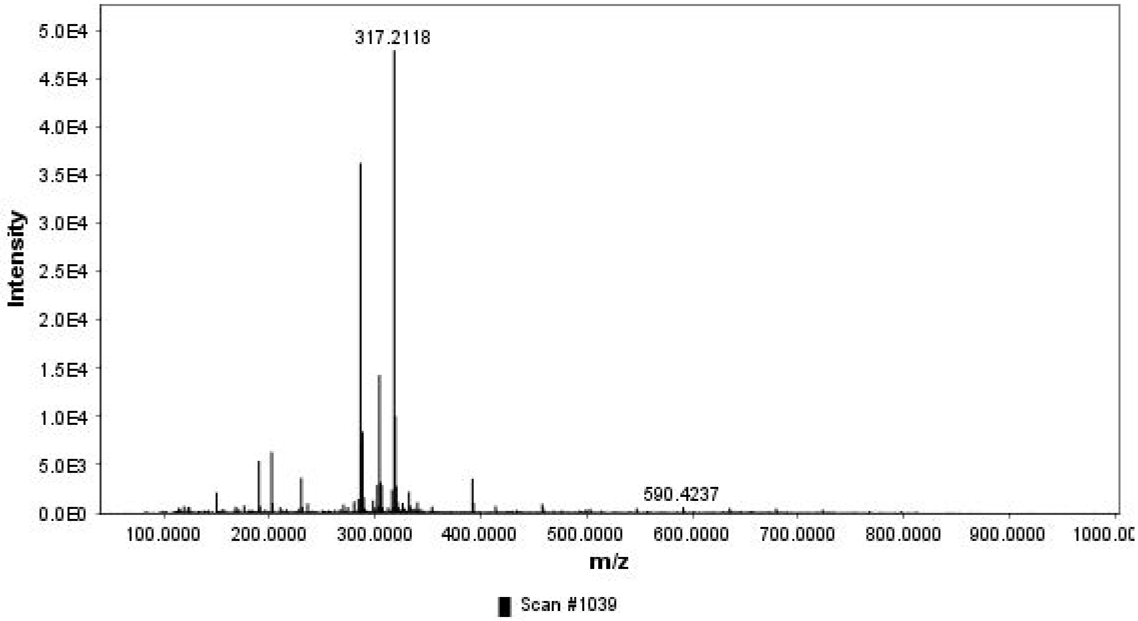

Supplementary Figure 52. High resolution mass spectrum of Pyranthanone A **(3)**.

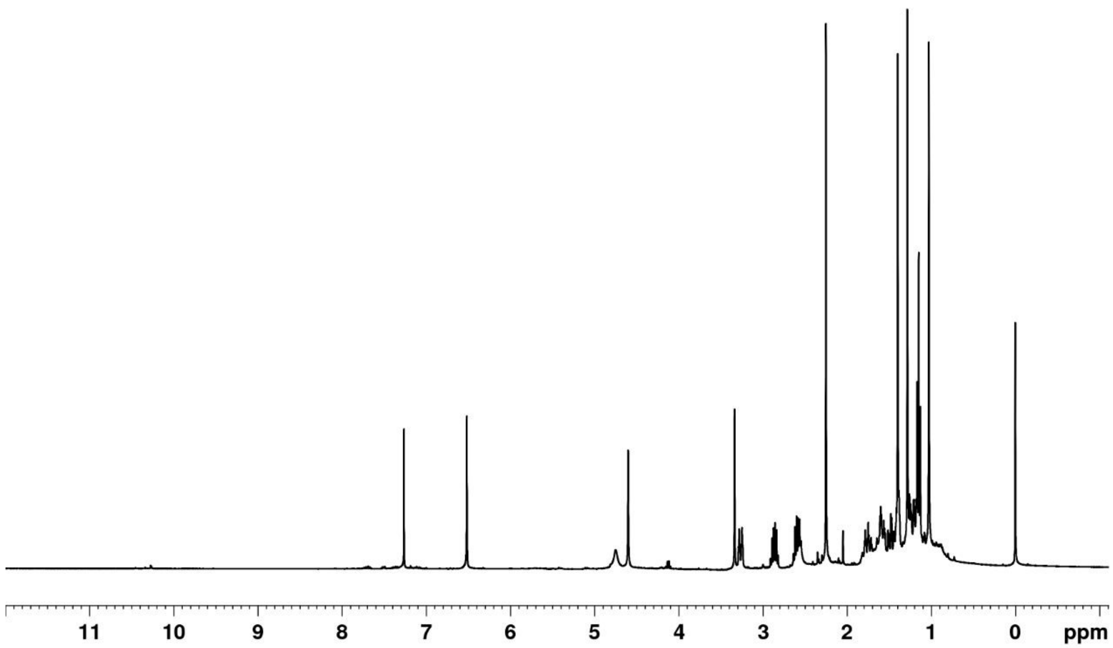

Supplementary Figure 53. ^{1}H NMR spectrum of Pyranthanone A **(3)** [600 MHz, $CDCl_3$, ppm].

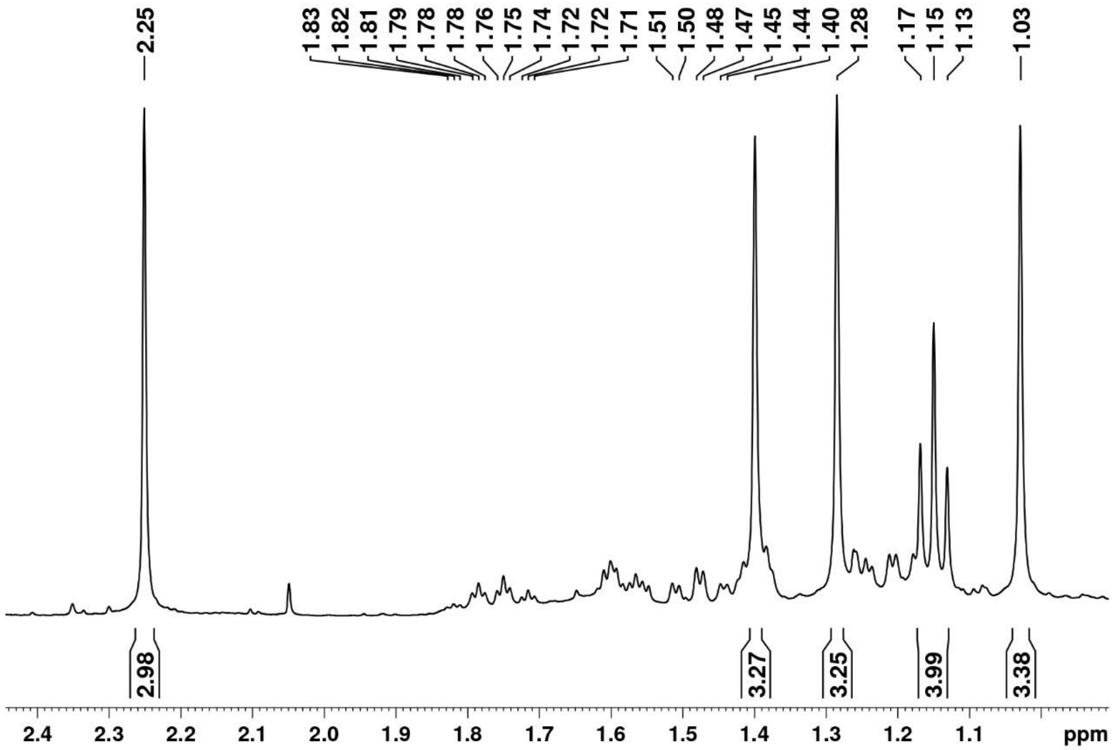

Supplementary Figure 54. ^{1}H NMR spectrum of Pyranthanone A **(3)** [600 MHz, $CDCl_3$, ppm].

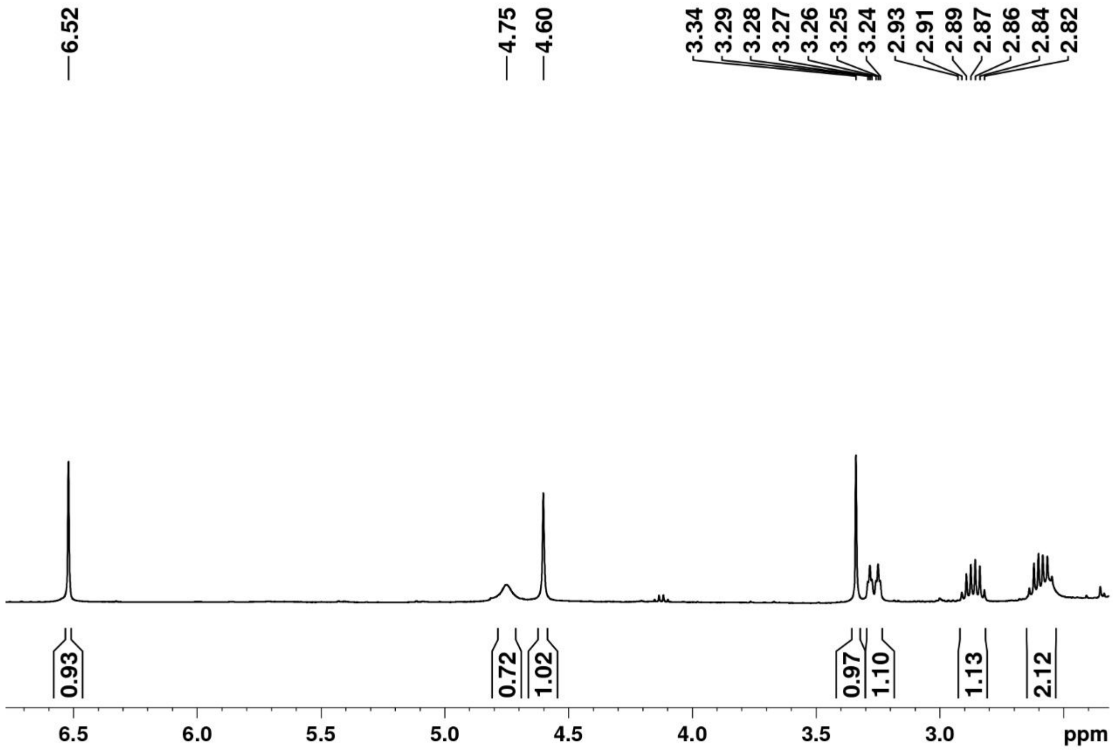

Supplementary Figure 55. ^{1}H NMR spectrum of Pyranthanone A **(3)** [600 MHz, $CDCl_3$, ppm].

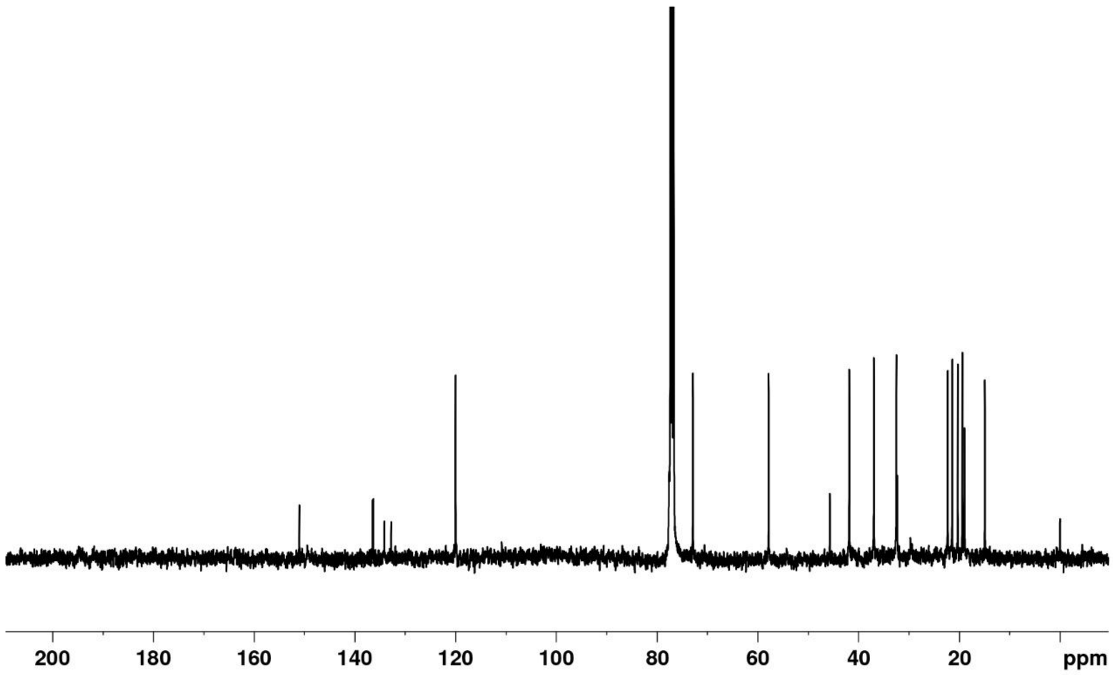

Supplementary Figure 56. ^{13}C NMR spectrum of Pyranthanone A **(3)** [150 MHz, $CDCl_3$, ppm].

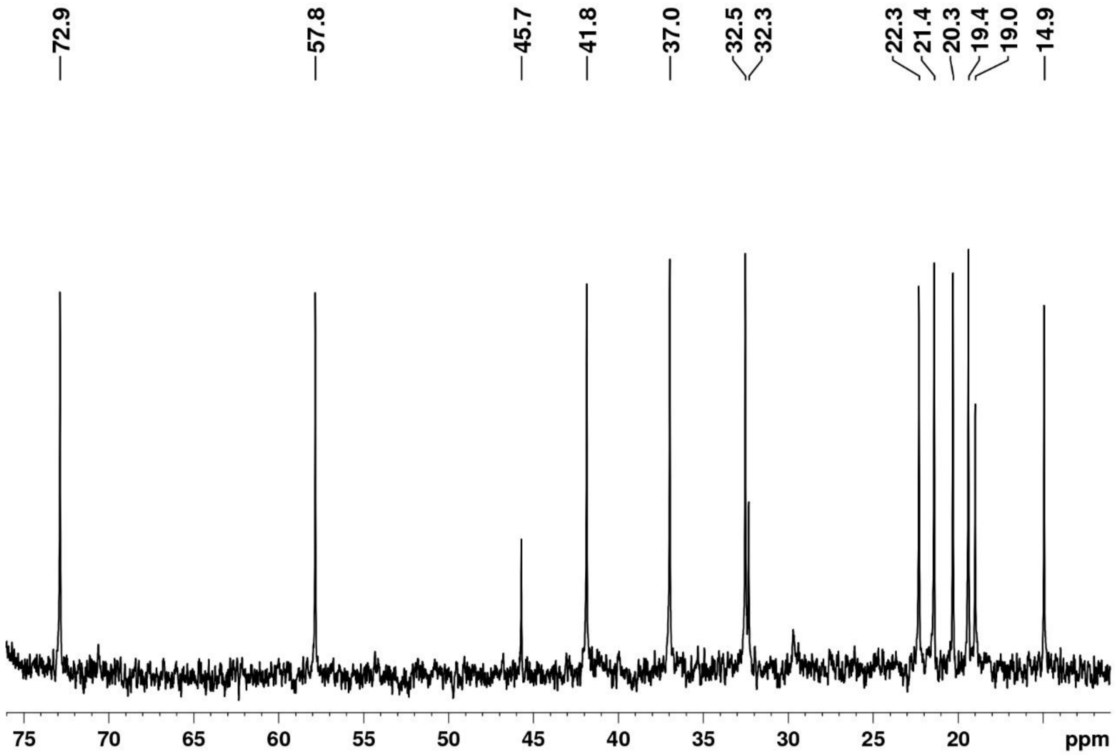

Supplementary Figure 57. ^{13}C NMR spectrum of Pyranthanone A **(3)** [150 MHz, $CDCl_3$, ppm].

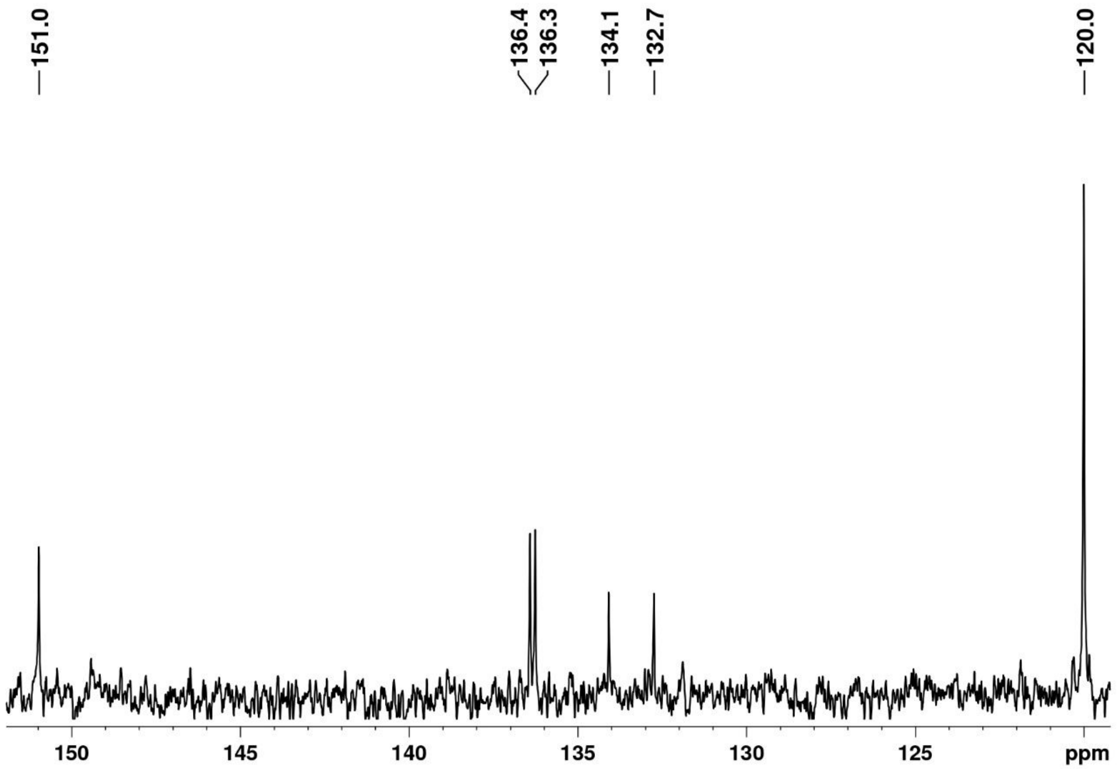

Supplementary Figure 58. ^{13}C NMR spectrum of Pyranthanone A **(3)** [150 MHz, $CDCl_3$, ppm].

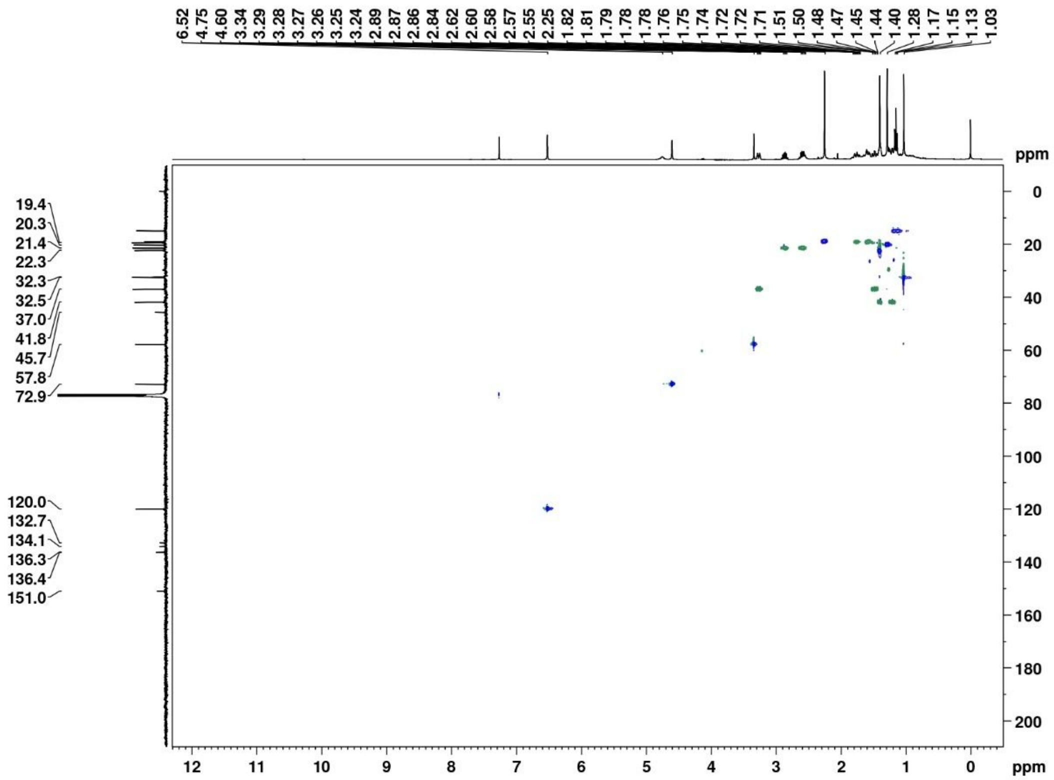

Supplementary Figure 59. HSQC NMR spectrum of Pyranthanone A **(3)** [600 MHz, $CDCl_3$, ppm].

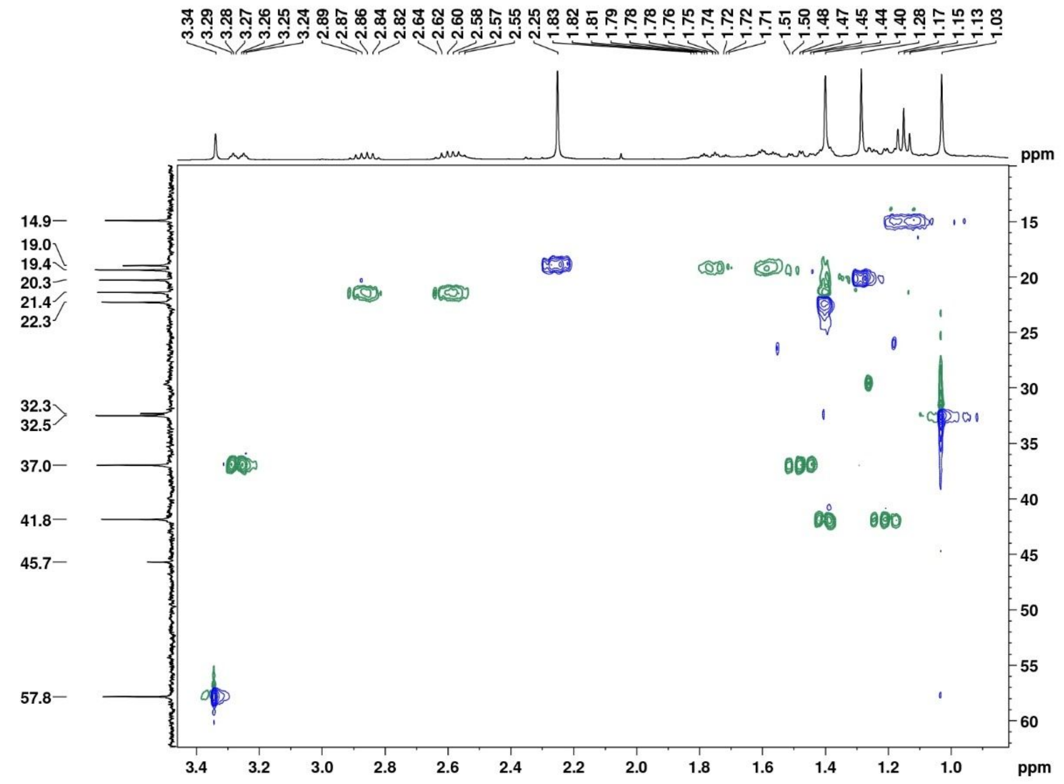

Supplementary Figure 60. HSQC NMR spectrum of Pyranthanone A **(3)** [600 MHz, $CDCl_3$, ppm].

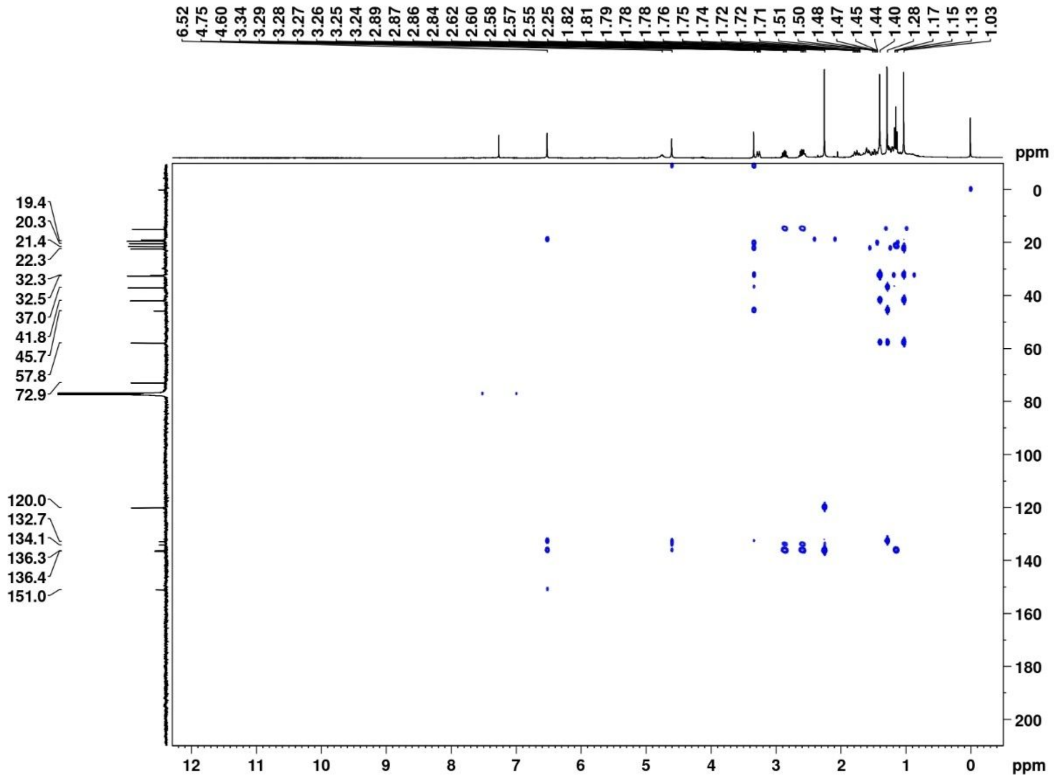

Supplementary Figure 61. HMBC NMR spectrum of Pyranthanone A **(3)** [600 MHz, $CDCl_3$, ppm].

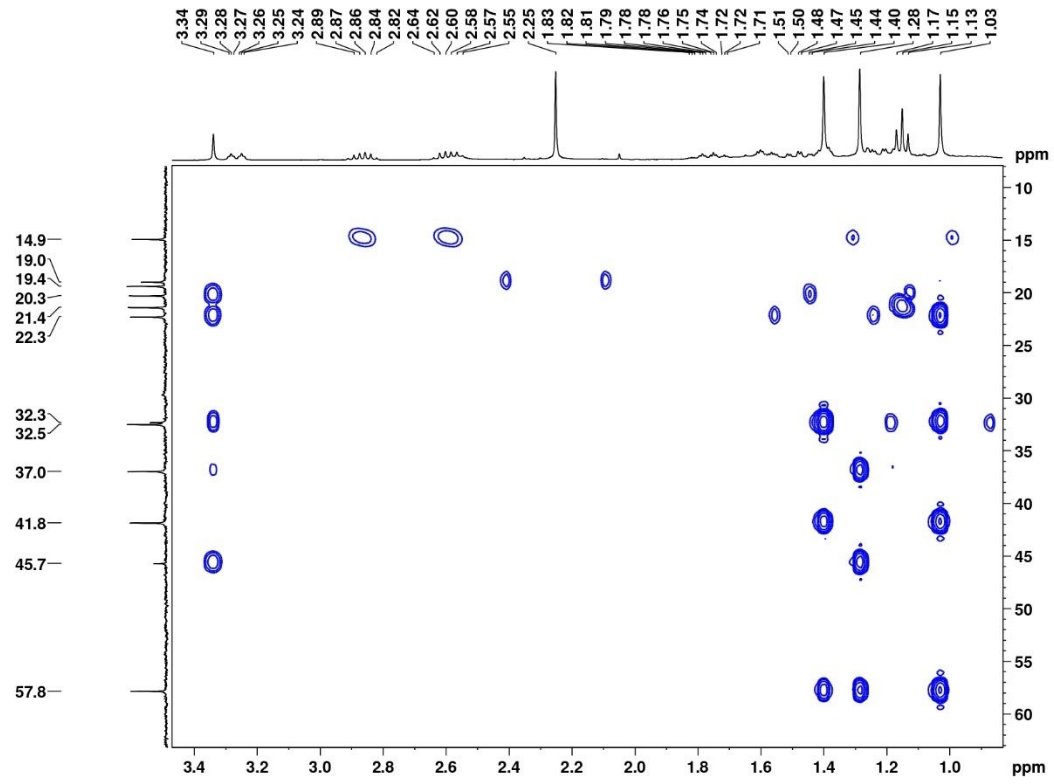

Supplementary Figure 62. HMBC NMR spectrum of Pyranthanone A **(3)** [600 MHz, $CDCl_3$, ppm].

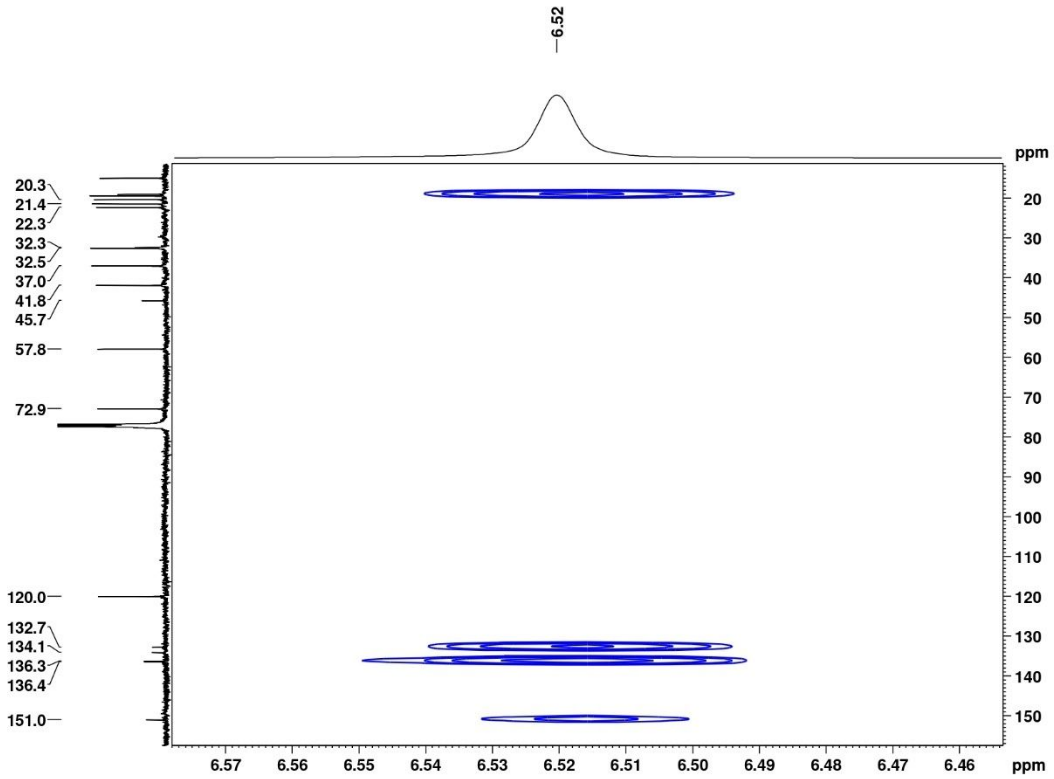

Supplementary Figure 63. HMBC NMR spectrum of Pyranthanone A **(3)** [600 MHz, $CDCl_3$, ppm].

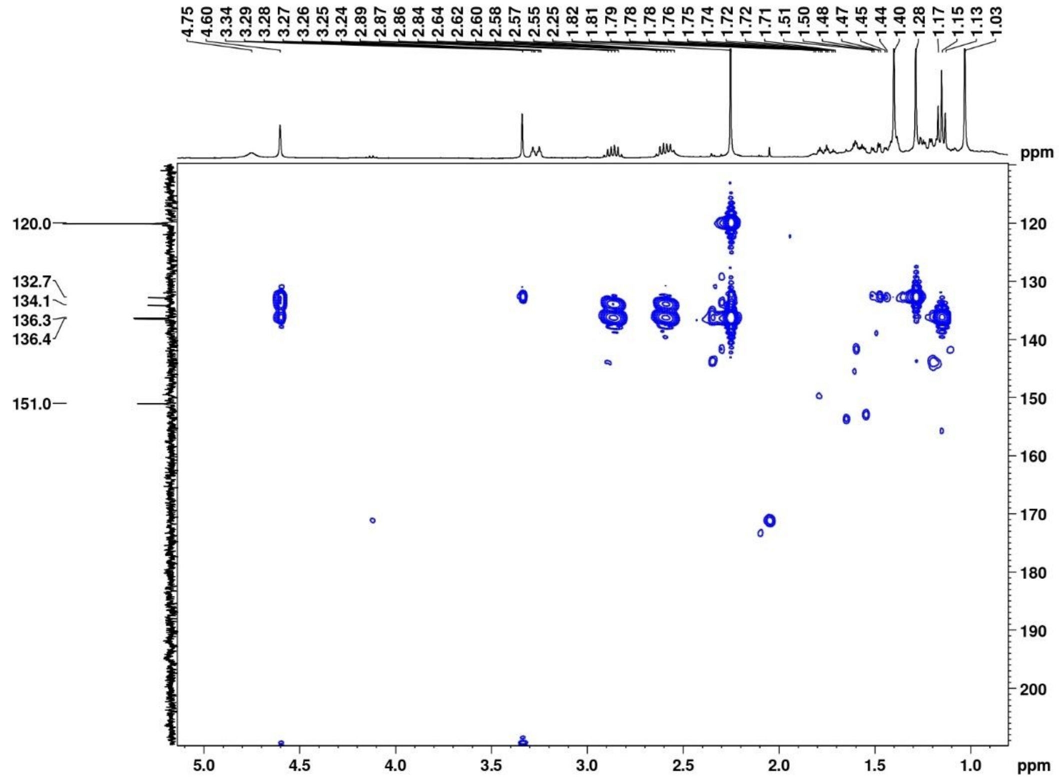

Supplementary Figure 64. HMBC NMR spectrum of Pyranthanone A **(3)** [600 MHz, $CDCl_3$, ppm].

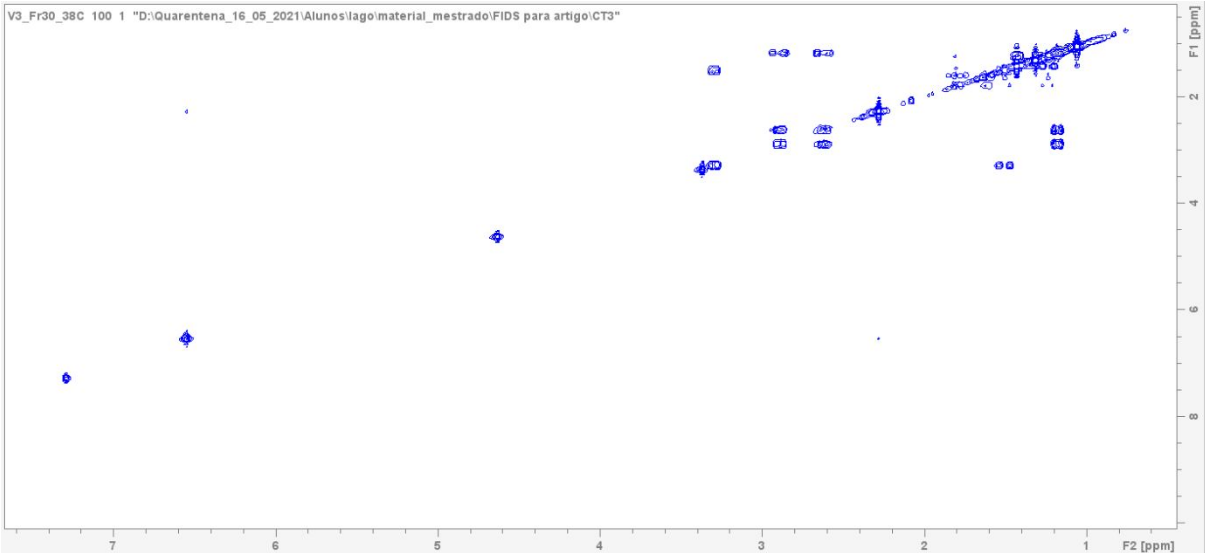

Supplementary Figure 65. COSY NMR spectrum of Pyranthanone A **(3)** [600 MHz, $CDCl_3$, ppm].

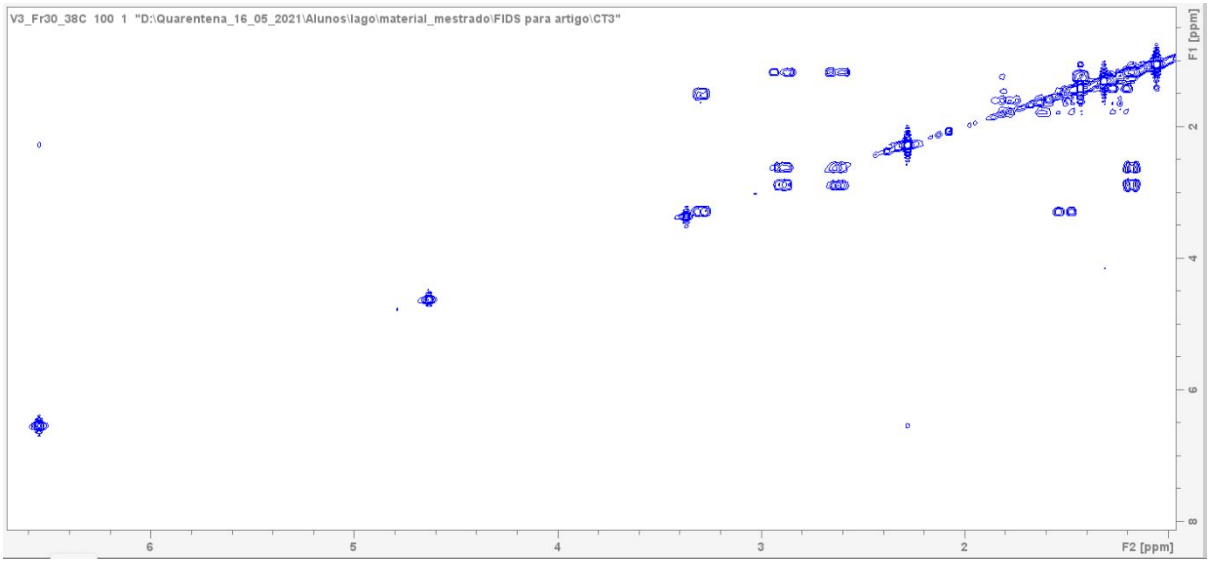

Supplementary Figure 66. COSY NMR spectrum of Pyranthanone A **(3)** [600 MHz, $CDCl_3$, ppm].

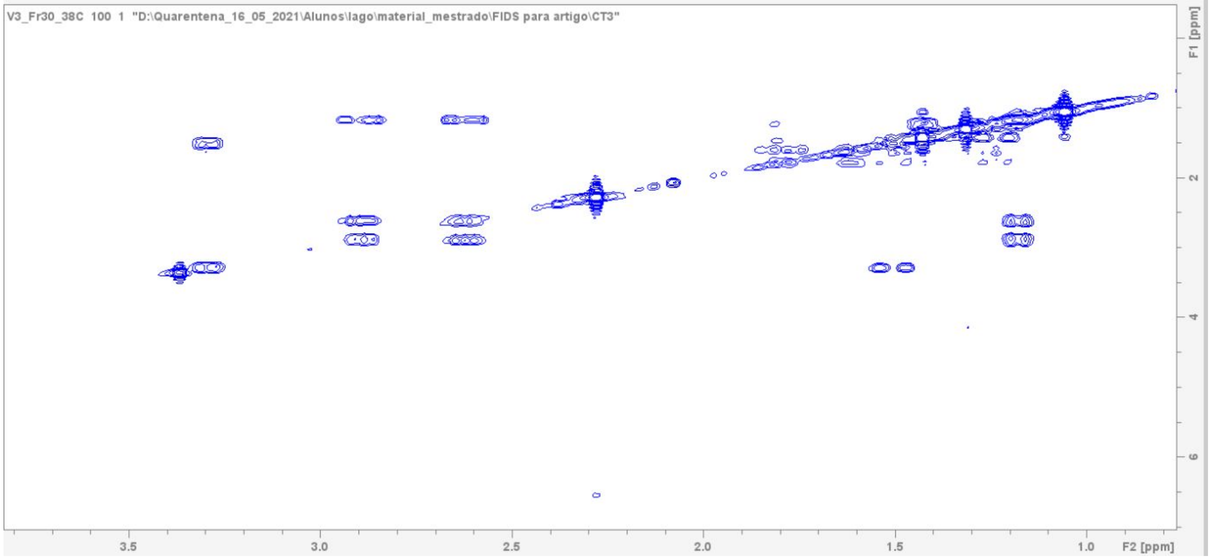

Supplementary Figure 67. COSY NMR spectrum of Pyranthanone A **(3)** [600 MHz, $CDCl_3$, ppm].

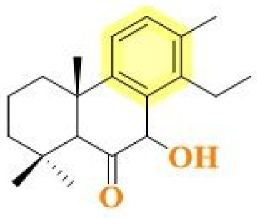

4

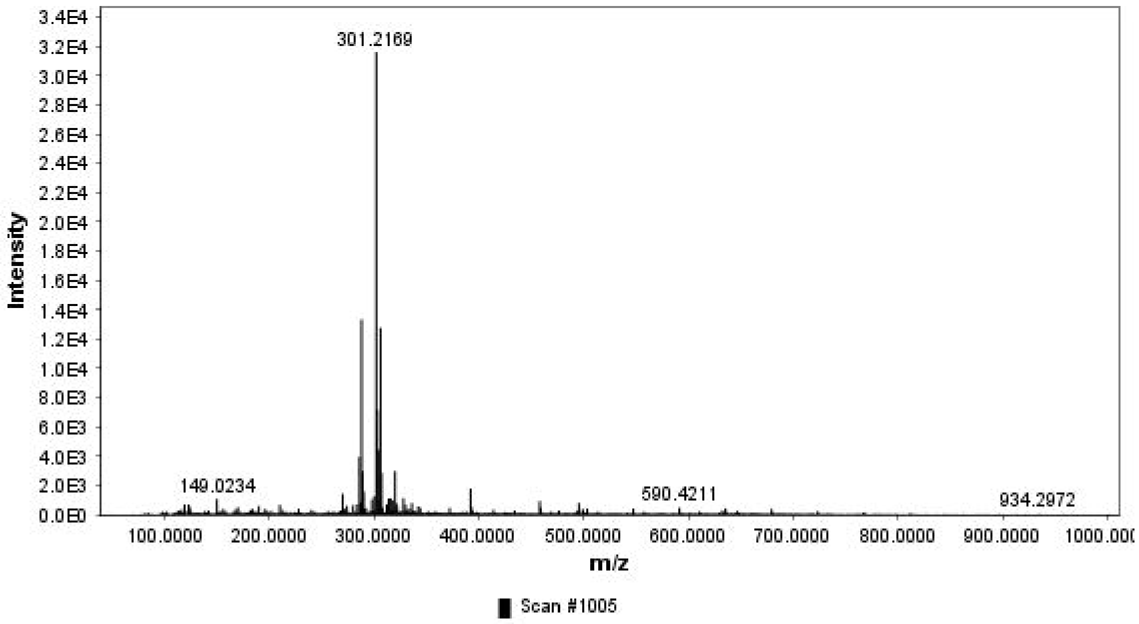

Supplementary Figure 68. High resolution mass spectrum of Pyranthanone B **(4)**.

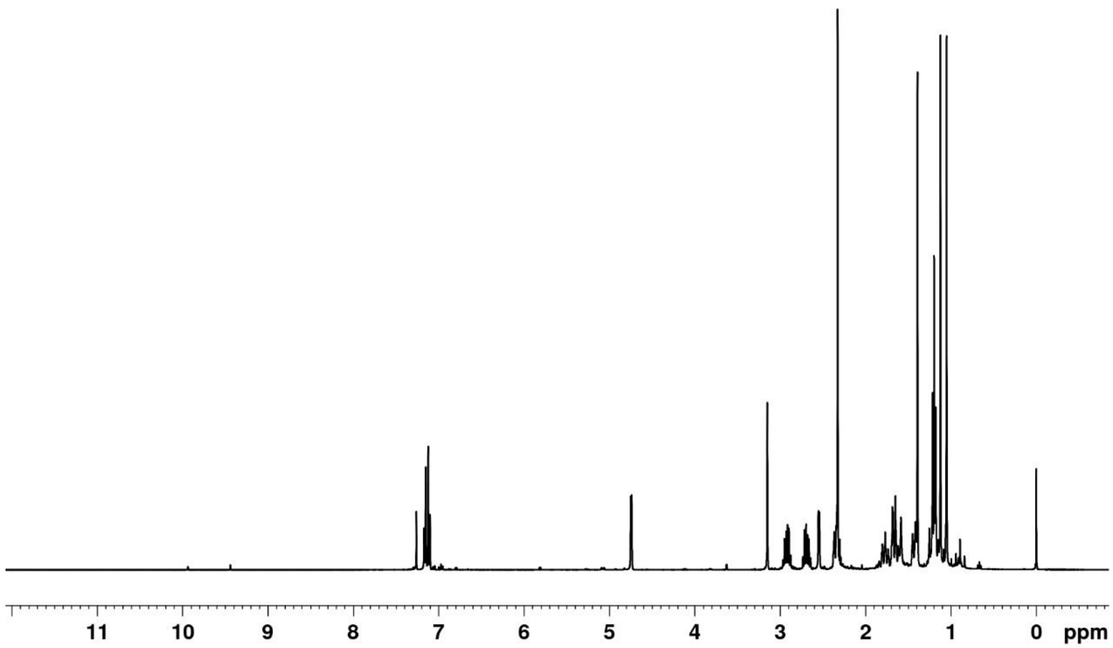

Supplementary Figure 69. ^{1}H NMR spectrum of Pyranthanone B **(4)** [600 MHz, $CDCl_3$, ppm].

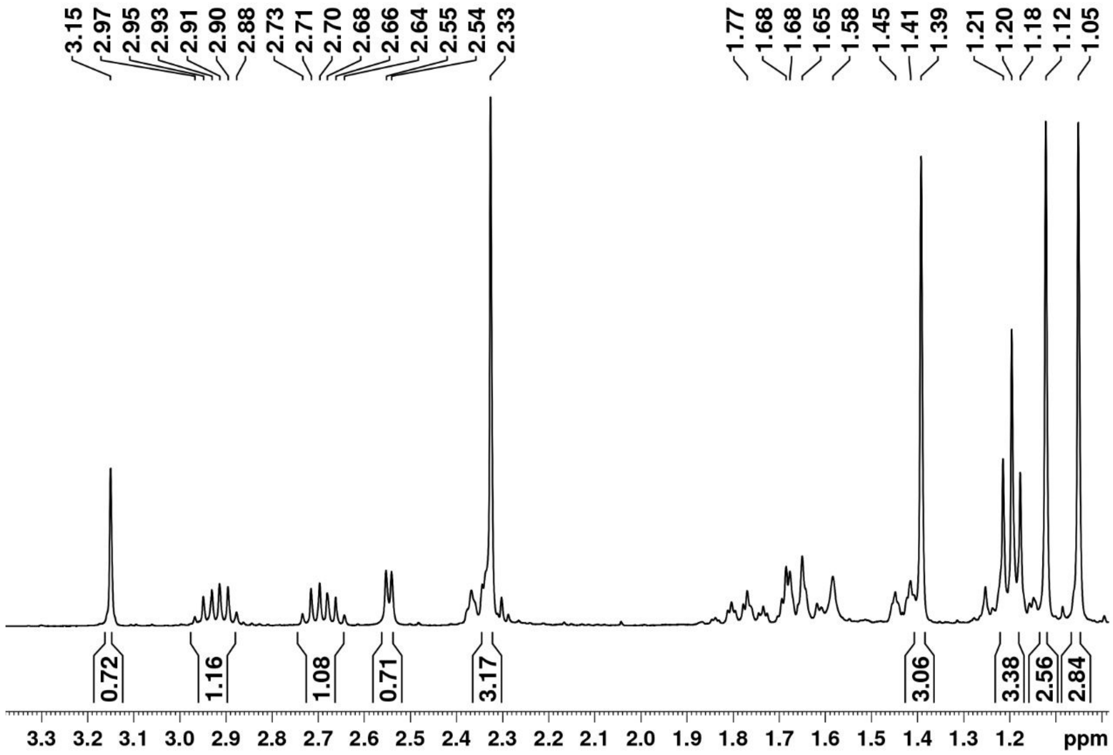

Supplementary Figure 70. ^{1}H NMR spectrum of Pyranthanone B **(4)** [600 MHz, $CDCl_3$, ppm].

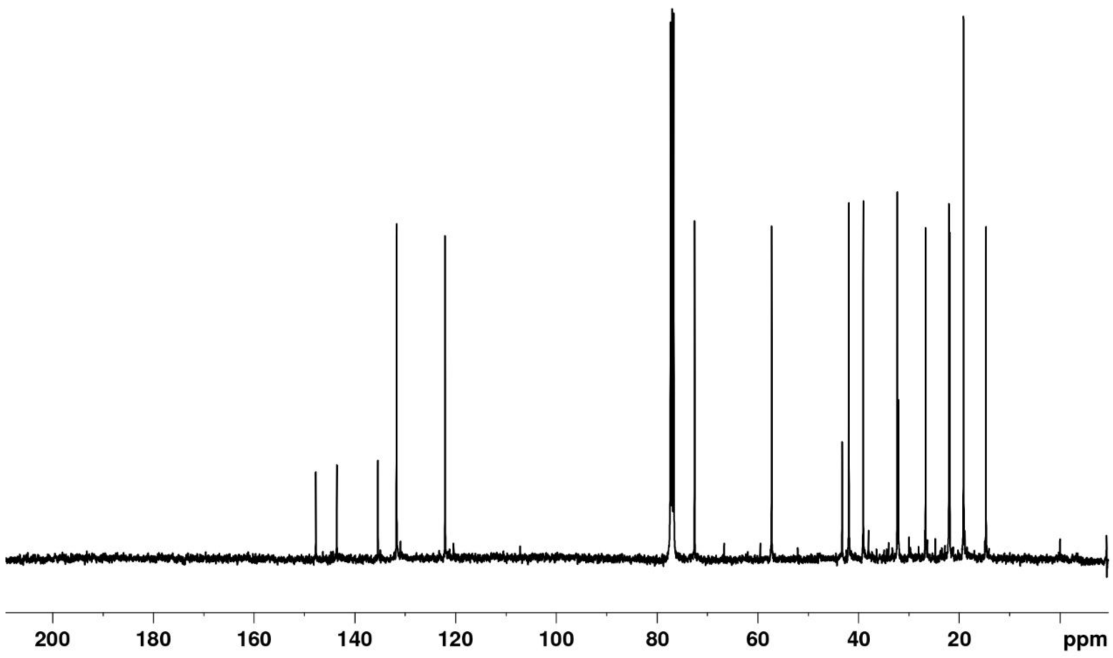

Supplementary Figure 71. ^{13}C NMR spectrum of Pyranthanone B **(4)** [150 MHz, $CDCl_3$, ppm].

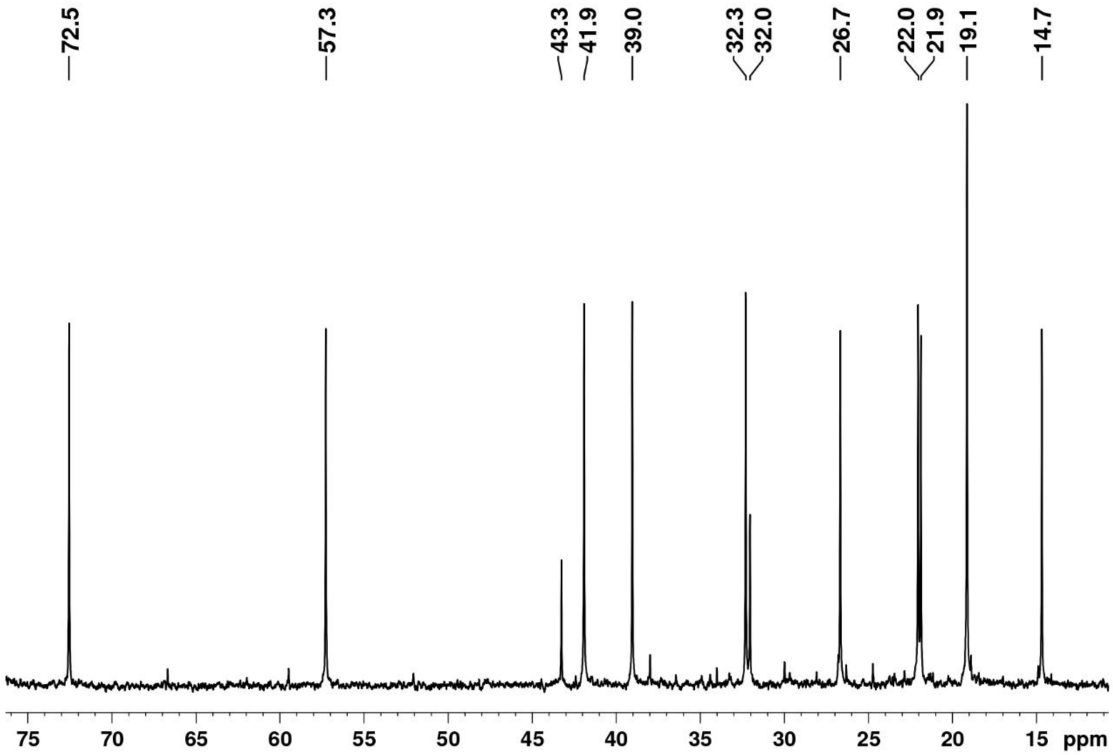

Supplementary Figure 72. ^{13}C NMR spectrum of Pyranthanone B **(4)** [150 MHz, $CDCl_3$, ppm].

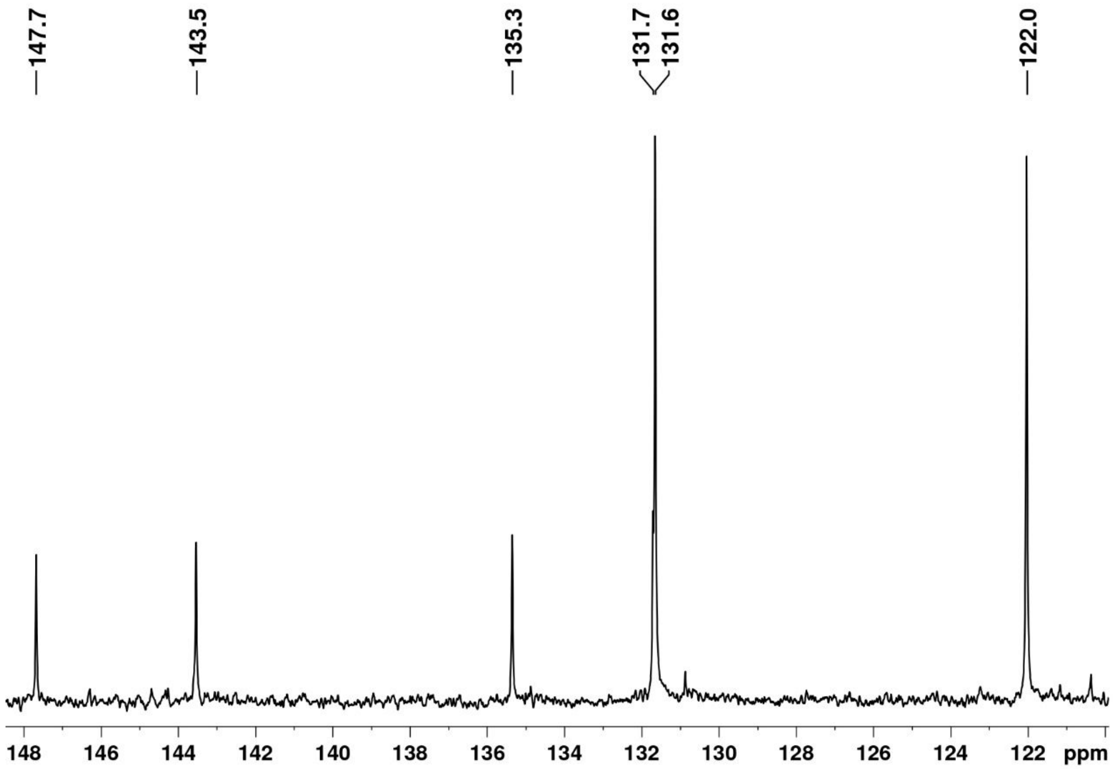

Supplementary Figure 73. ^{13}C NMR spectrum of Pyranthanone B **(4)** [150 MHz, $CDCl_3$, ppm].

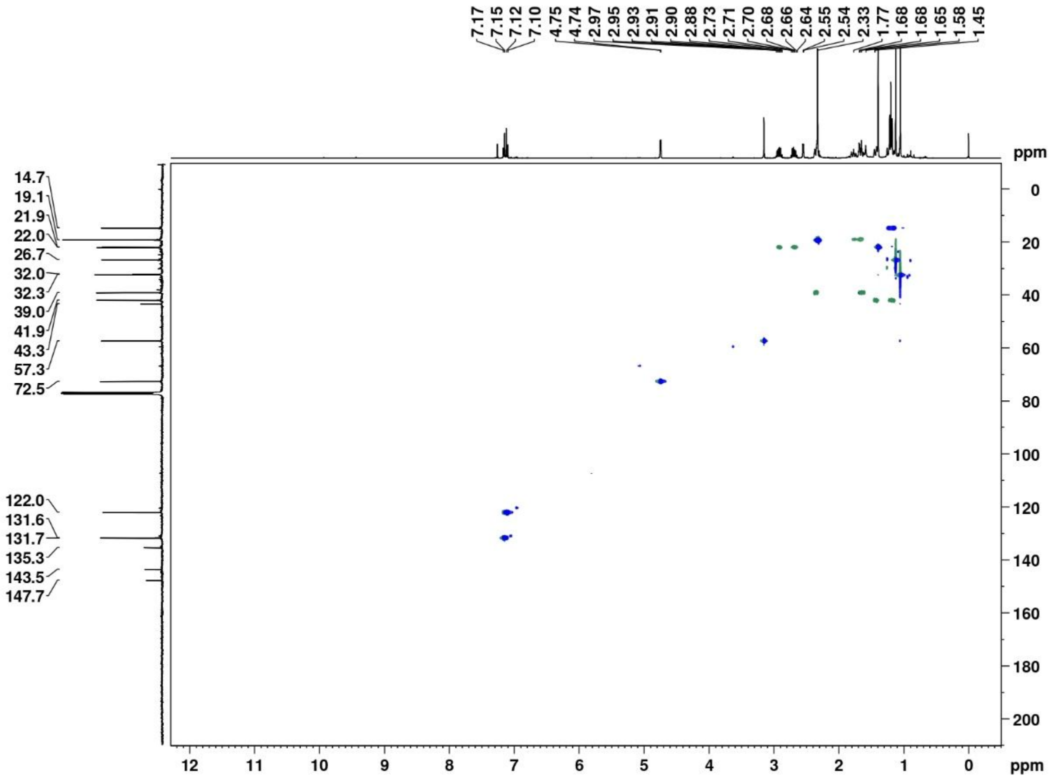

Supplementary Figure 74. HSQC NMR spectrum of Pyranthanone B **(4)** [600 MHz, $CDCl_3$, ppm].

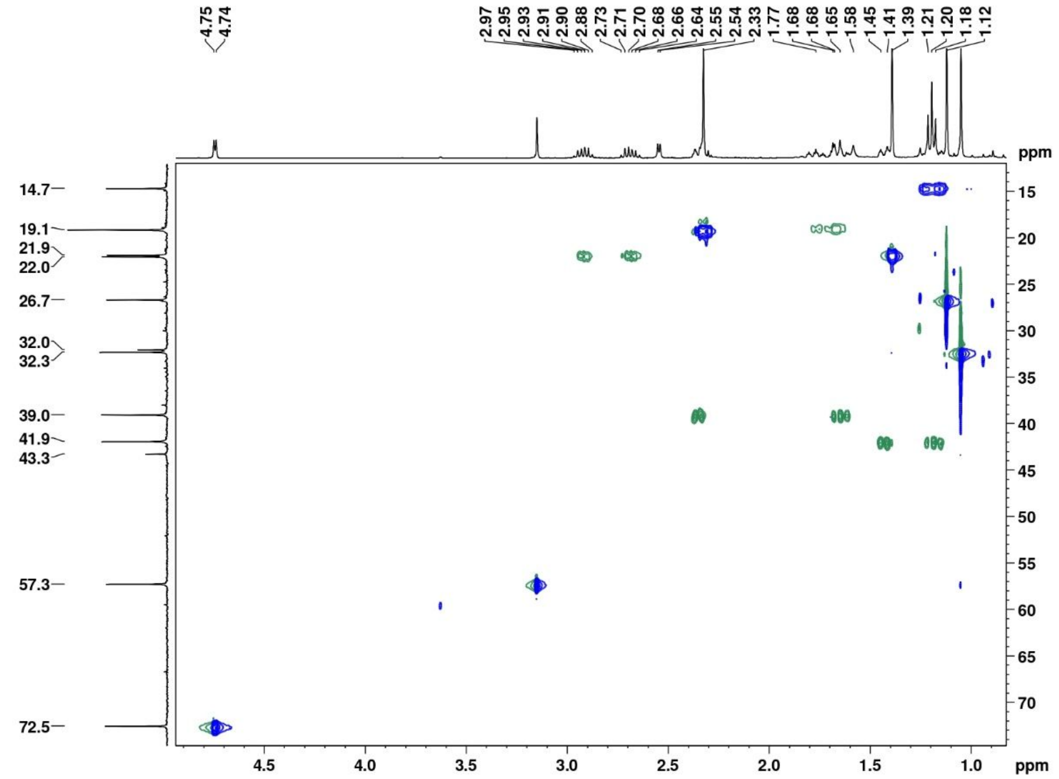

Supplementary Figure 75. HSQC NMR spectrum of Pyranthanone B **(4)** [600 MHz, $CDCl_3$, ppm].

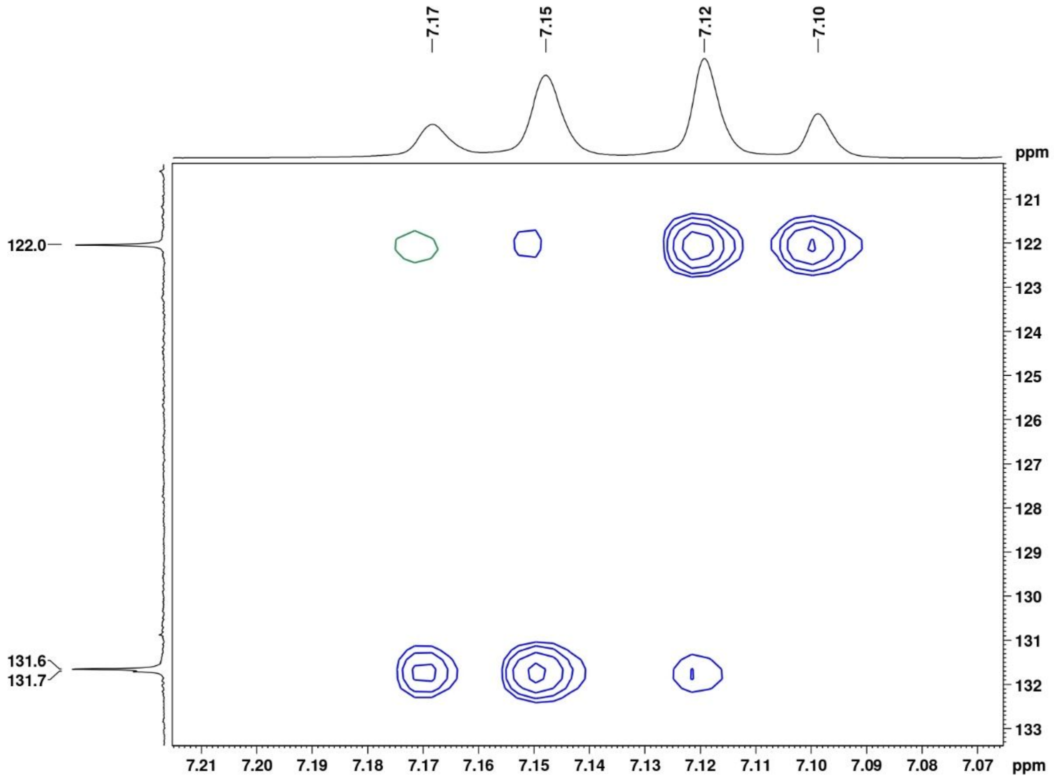

Supplementary Figure 76. HSQC NMR spectrum of Pyranthanone B **(4)** [600 MHz, $CDCl_3$, ppm].

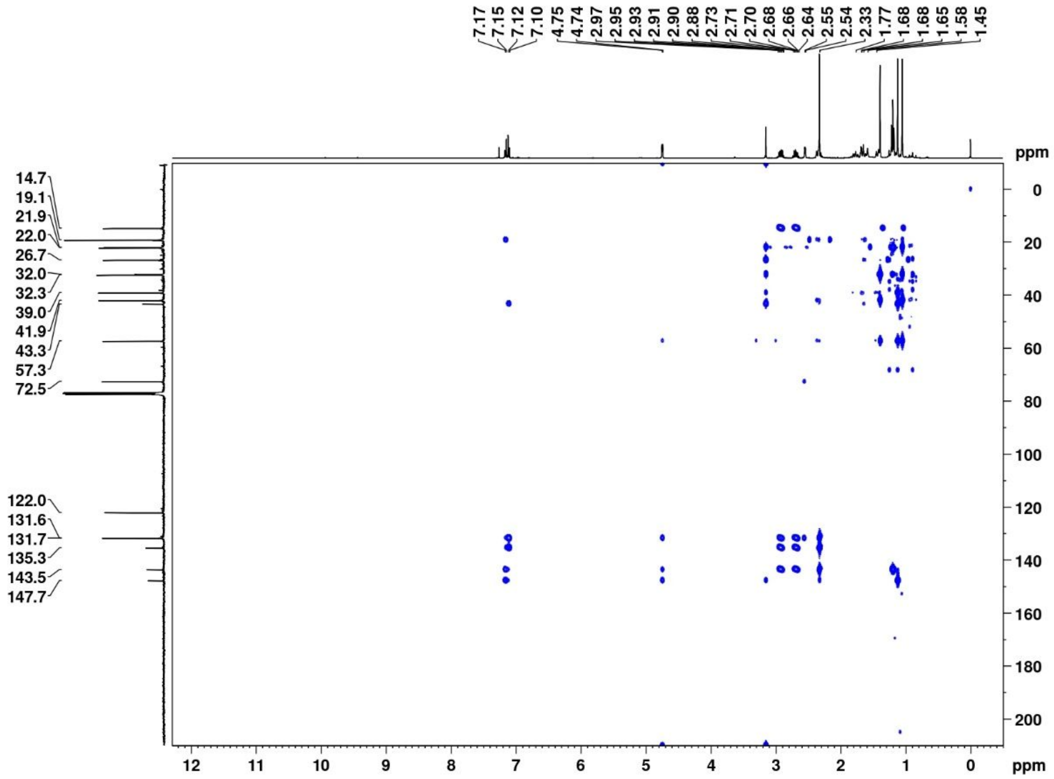

Supplementary Figure 77. HMBC NMR spectrum of Pyranthanone B **(4)** [600 MHz, $CDCl_3$, ppm].

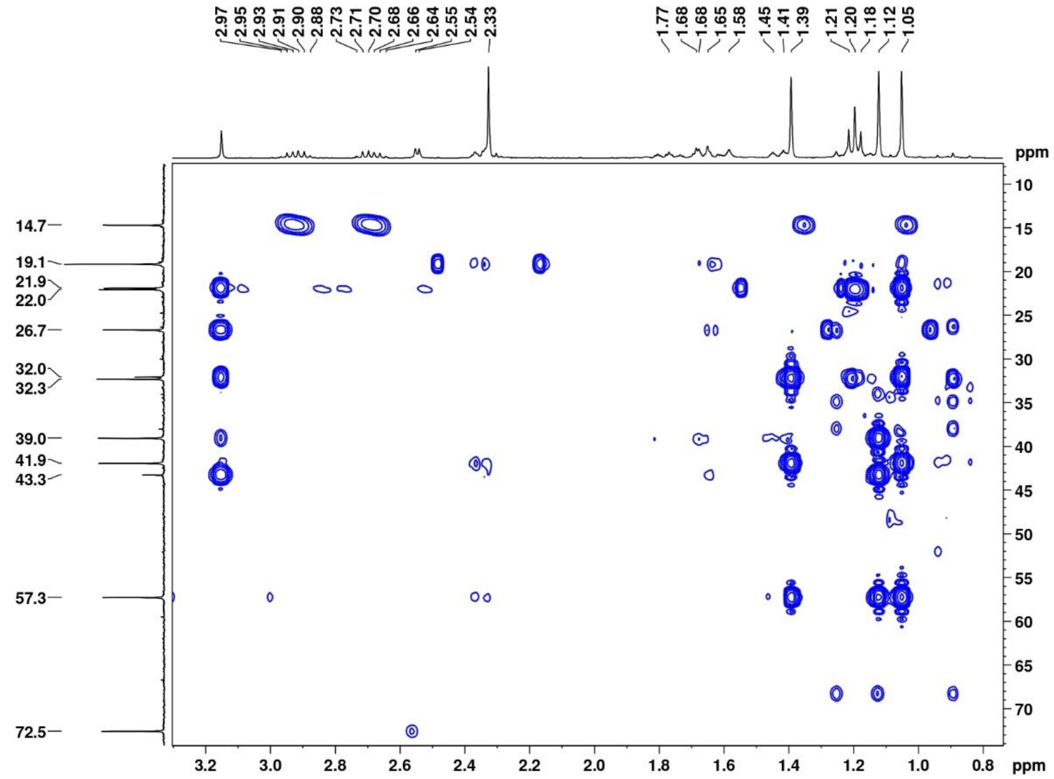

Supplementary Figure 78. HMBC NMR spectrum of Pyranthanone B **(4)** [600 MHz, $CDCl_3$, ppm].

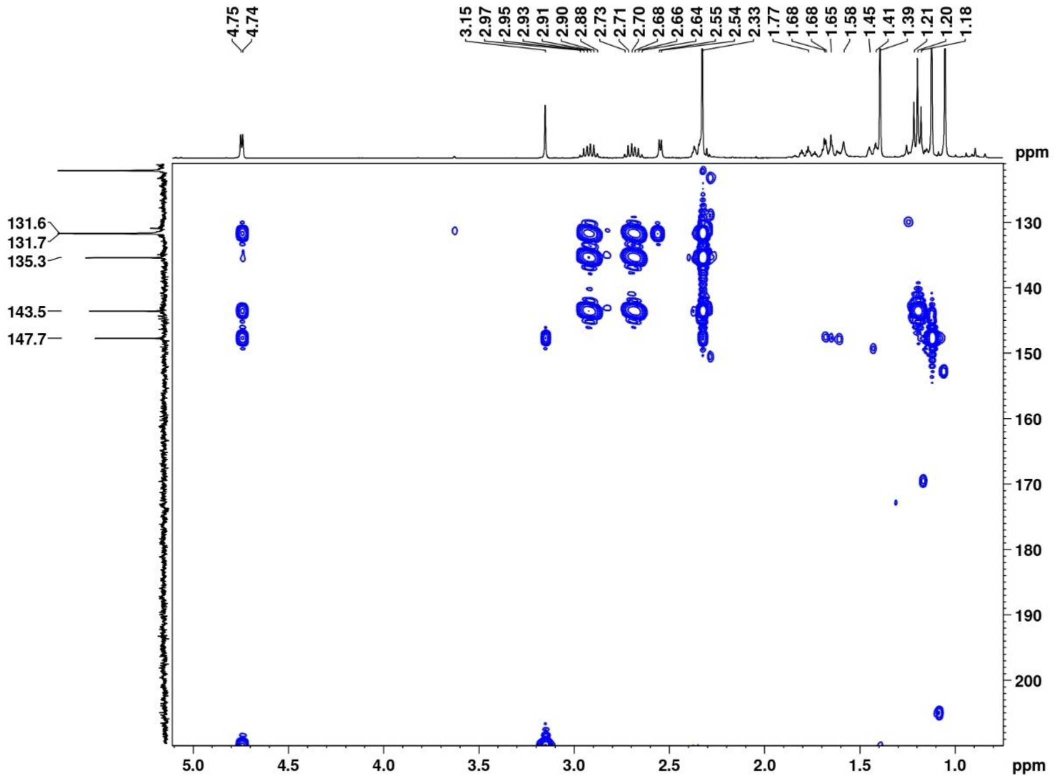

Supplementary Figure 79. HMBC NMR spectrum of Pyranthanone B **(4)** [600 MHz, $CDCl_3$, ppm].

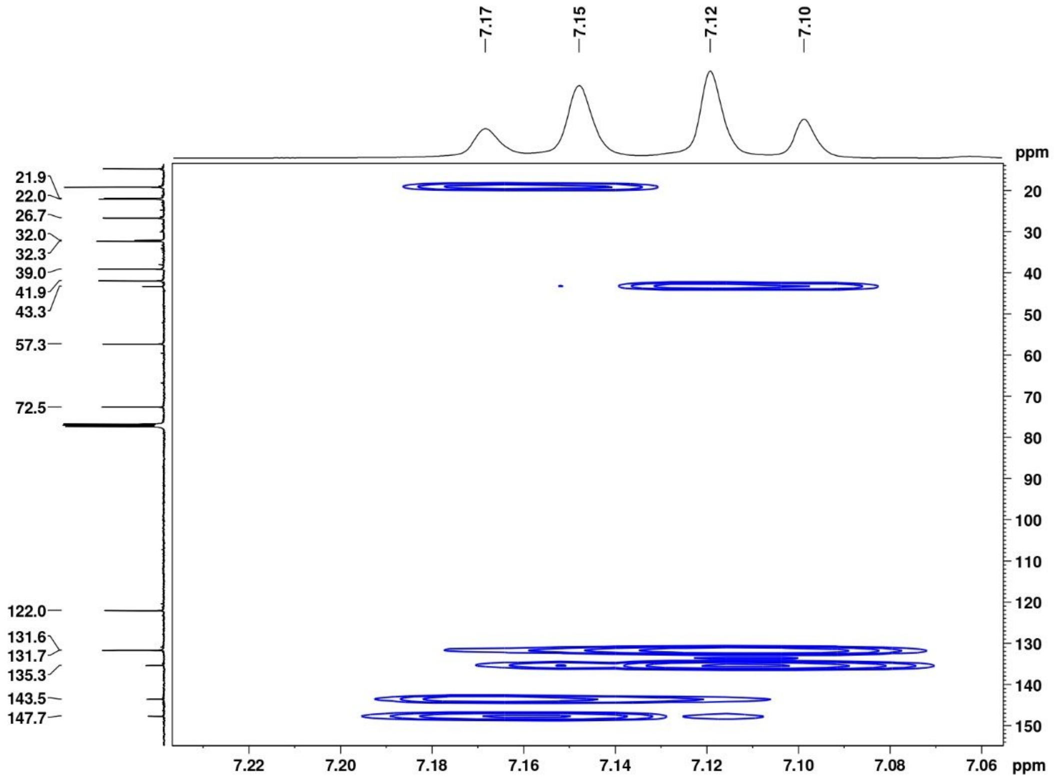

Supplementary Figure 80. HMBC NMR spectrum of Pyranthanone B **(4)** [600 MHz, $CDCl_3$, ppm].

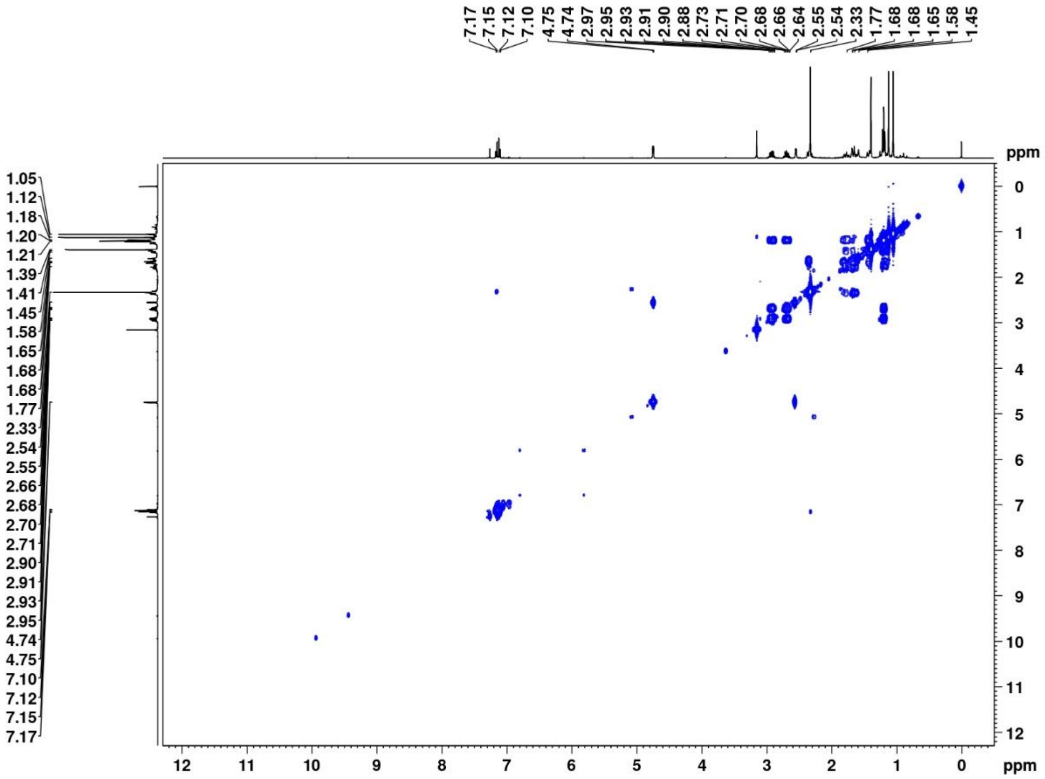

Supplementary Figure 81. COSY NMR spectrum of Pyranthanone B **(4)** [600 MHz, $CDCl_3$, ppm].

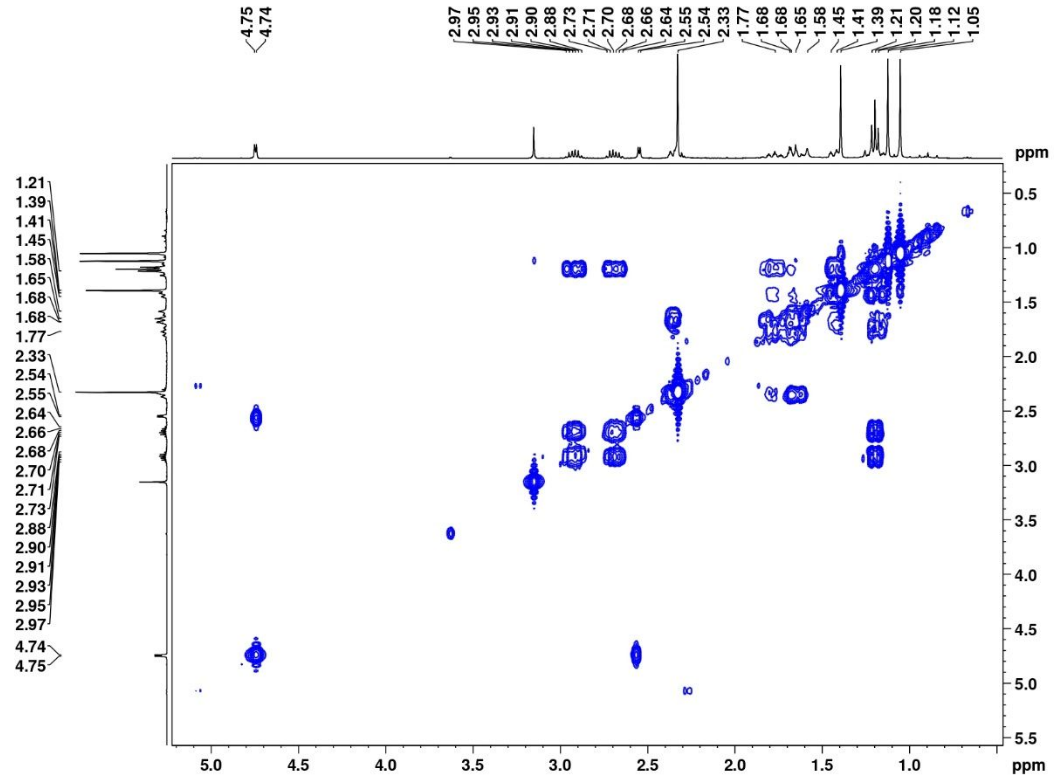

Supplementary Figure 82. COSY NMR spectrum of Pyranthanone B **(4)** [600 MHz, $CDCl_3$, ppm].

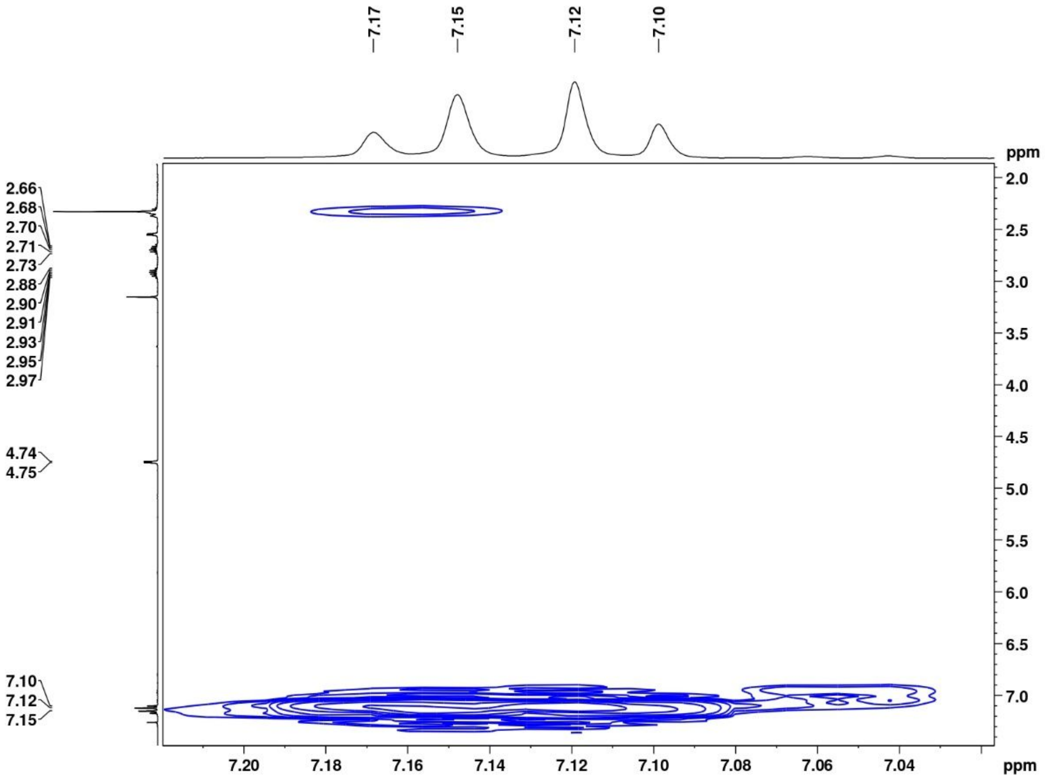

Supplementary Figure 83. COSY NMR spectrum of Pyranthanone B **(4)** [600 MHz, $CDCl_3$, ppm].

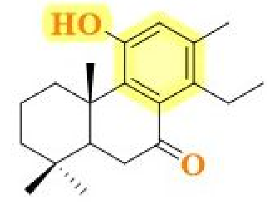

5

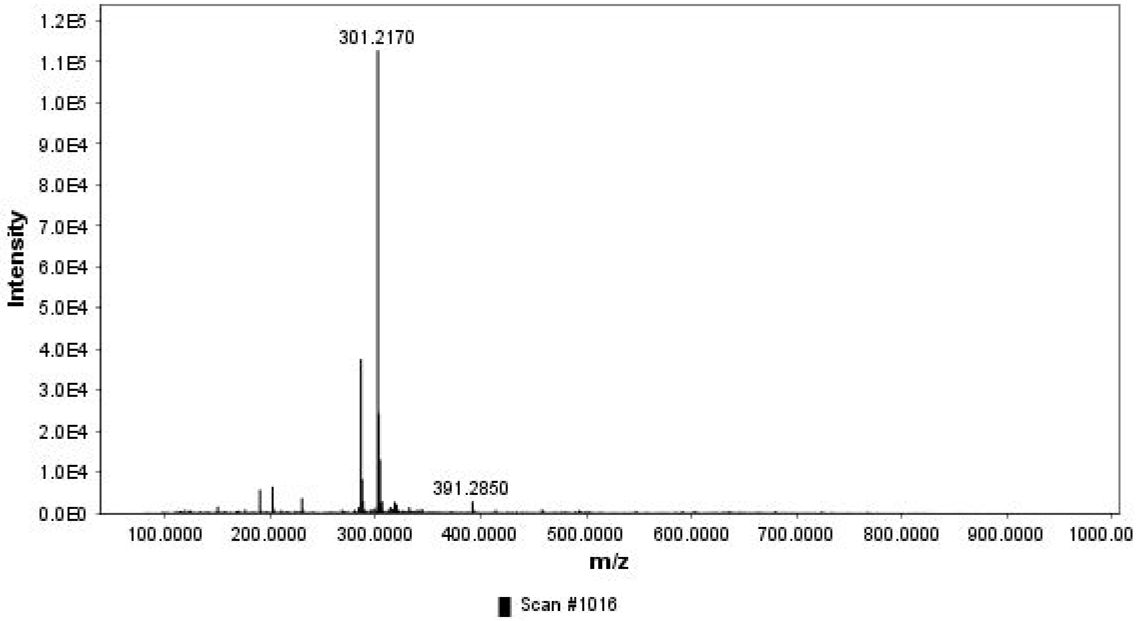

Supplementary Figure 84. High resolution mass spectrum of 11-hydroxy-cleisthantan-8,11,13-trien-7-one **(5).**

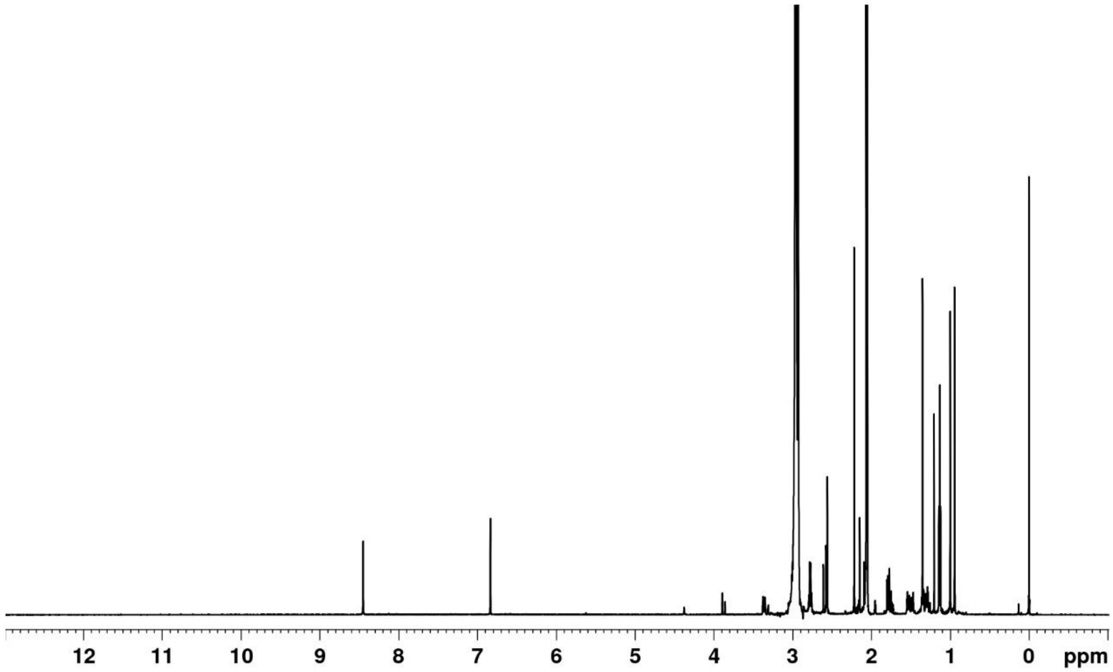

Supplementary Figure 85. ^{1}H NMR spectrum of 11-hydroxy-cleisthantan-8,11,13-trien-7-one **(5)** [600 MHz, acetone-d6, ppm].

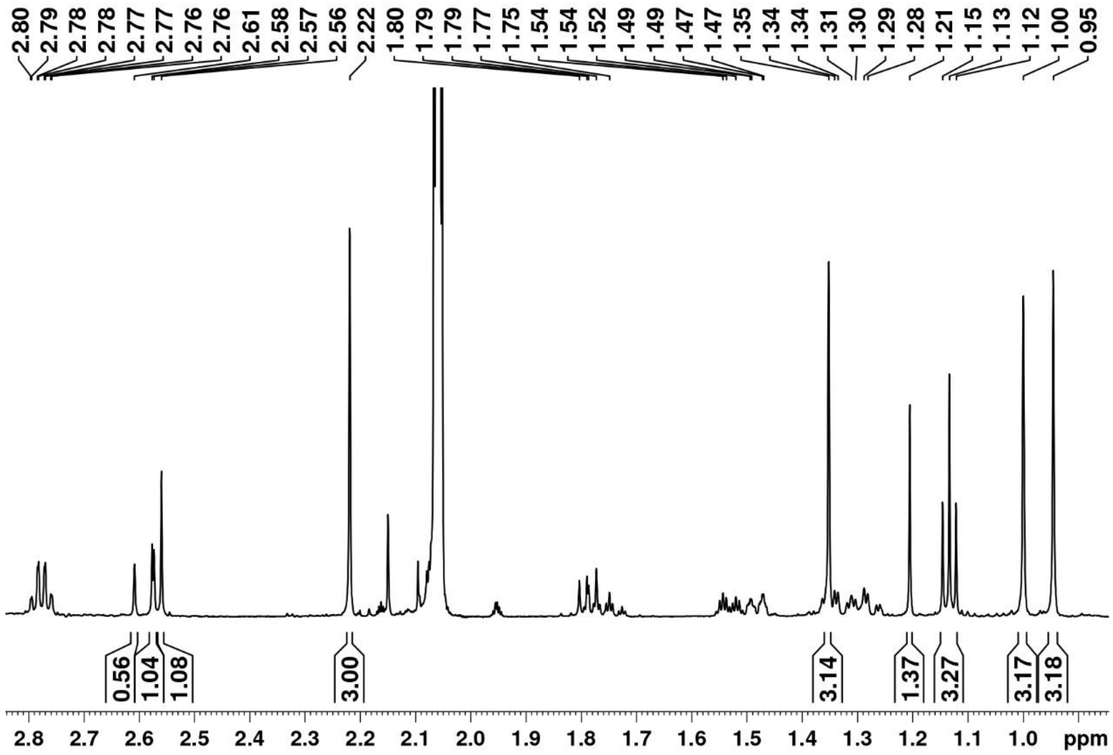

Supplementary Figure 86. ^{1}H NMR spectrum of 11-hydroxy-cleisthantan-8,11,13-trien-7-one **(5)** [600 MHz, acetone-d6, ppm].

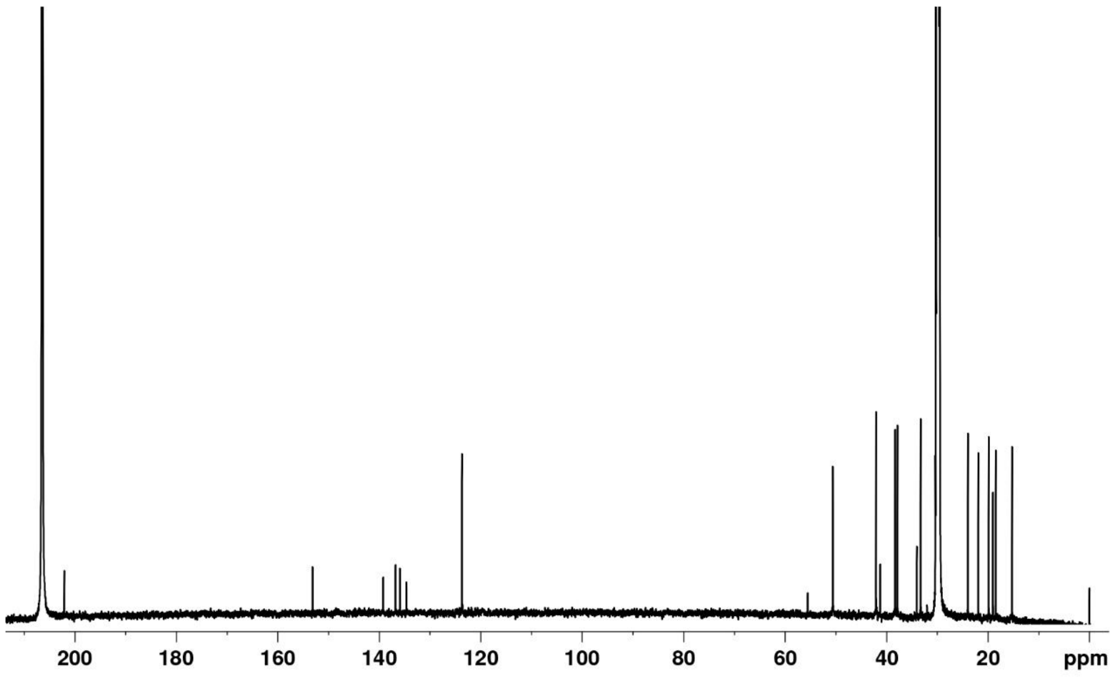

Supplementary Figure 87. ^{13}C NMR spectrum of 11-hydroxy-cleisthantan-8,11,13-trien-7-one **(5)** [150 MHz, acetone-d6, ppm].

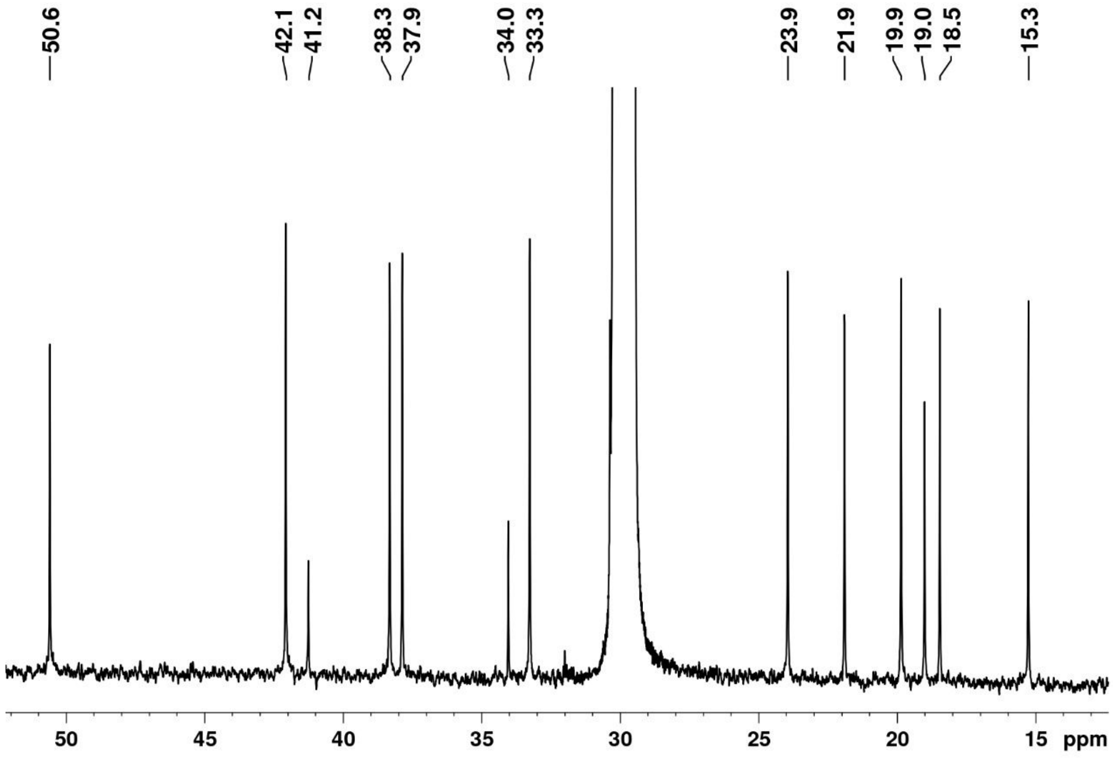

Supplementary Figure 88. ^{13}C NMR spectrum of 11-hydroxy-cleisthantan-8,11,13-trien-7-one **(5)** [150 MHz, acetone-d6, ppm].

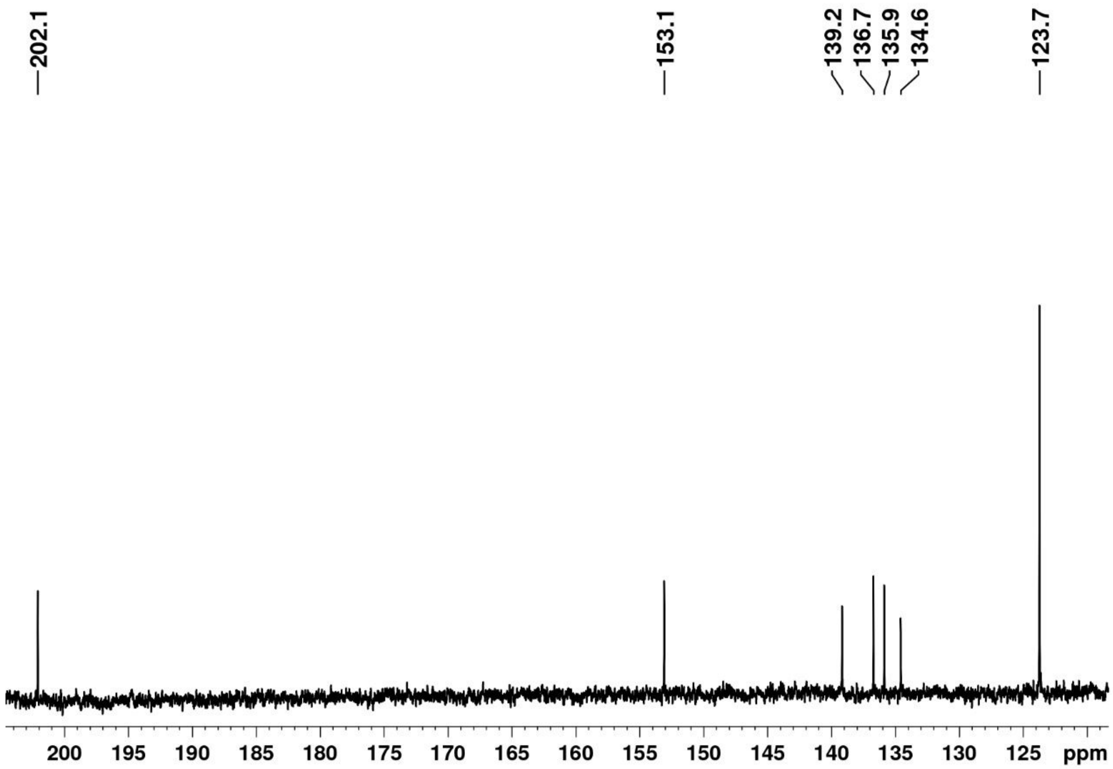

Supplementary Figure 89. ^{13}C NMR spectrum of 11-hydroxy-cleisthantan-8,11,13-trien-7-one **(5)** [150 MHz, acetone-d6, ppm].

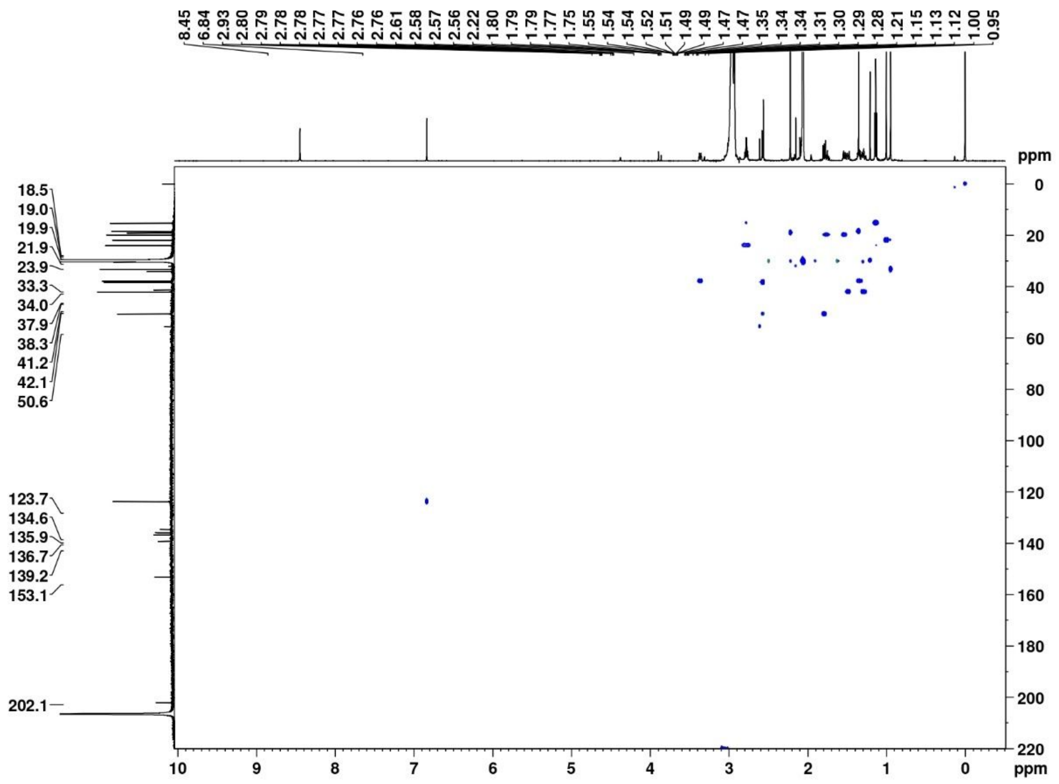

Supplementary Figure 90. HSQC NMR spectrum of 11-hydroxy-cleisthantan-8,11,13-trien-7-one **(5)** [600 MHz, acetone-d6, ppm].

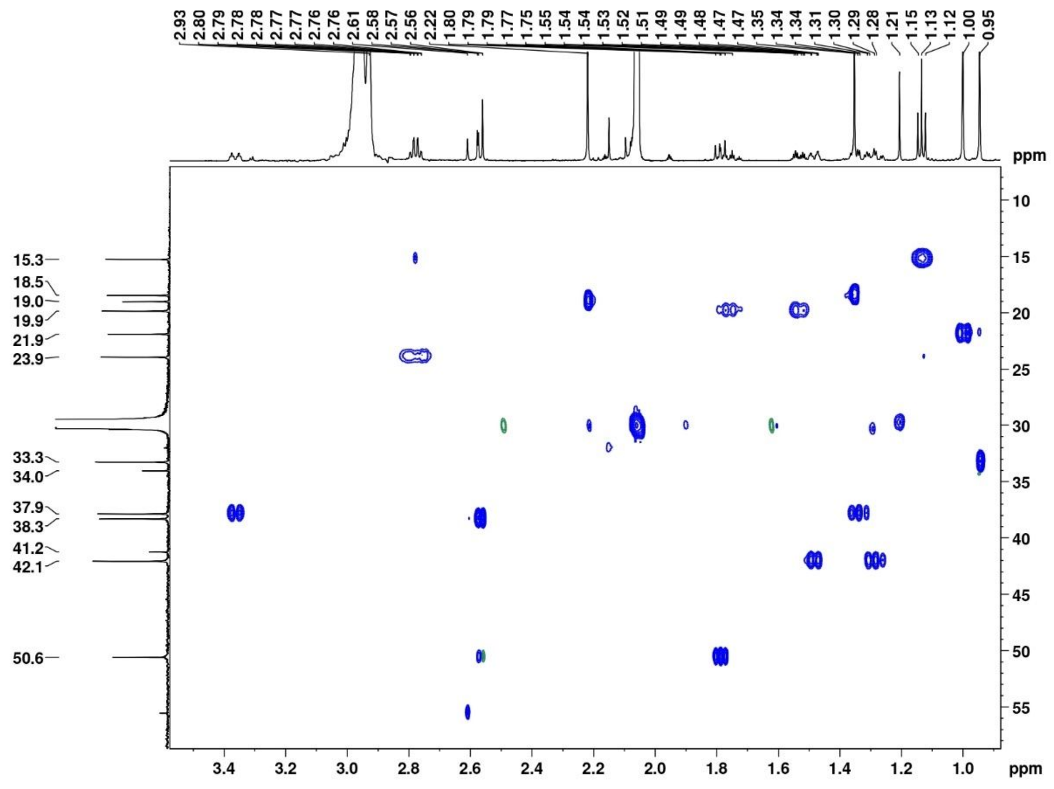

Supplementary Figure 91. HSQC NMR spectrum of 11-hydroxy-cleisthantan-8,11,13-trien-7-one **(5)** [600 MHz, acetone-d6 ppm].

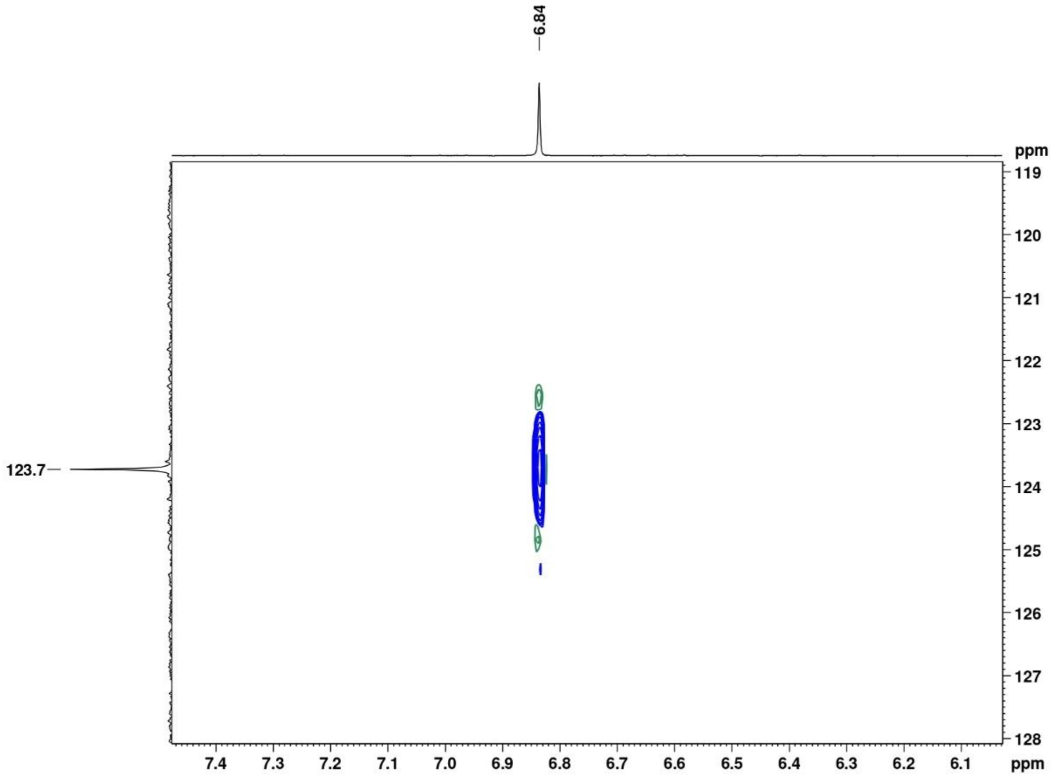

Supplementary Figure 92. HSQC NMR spectrum of 11-hydroxy-cleisthantan-8,11,13-trien-7-one **(5)** [600 MHz, acetone-d6, ppm].

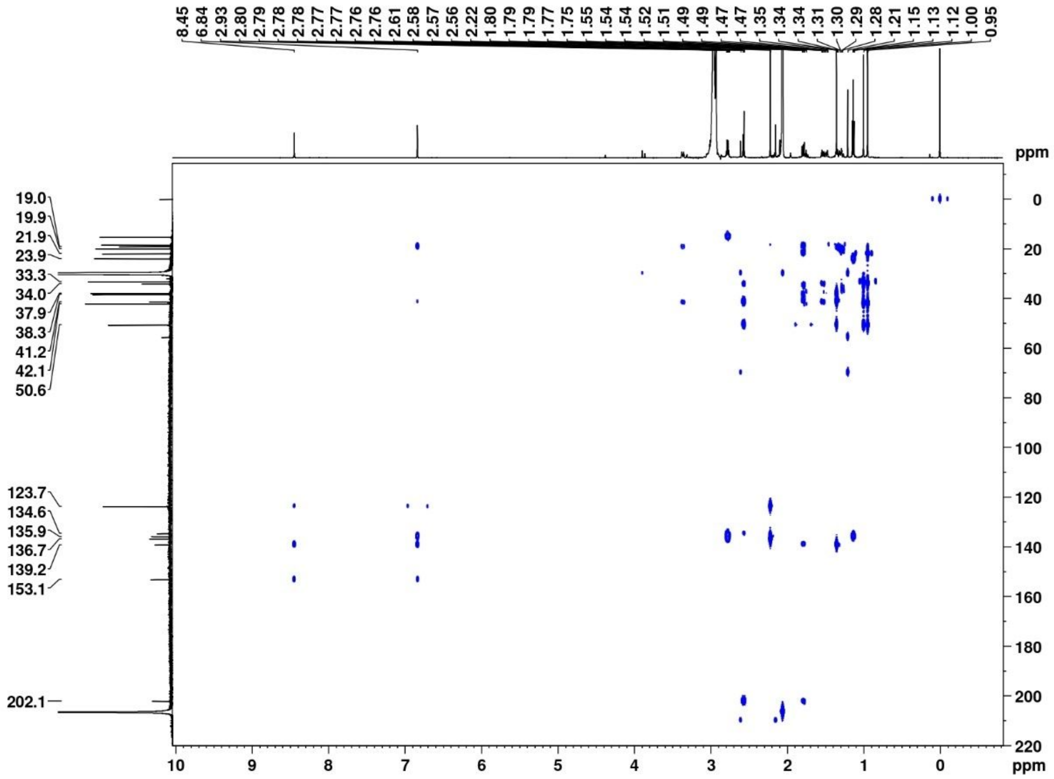

Supplementary Figure 93. HMBC NMR spectrum of 11-hydroxy-cleisthantan-8,11,13-trien-7-one **(5)** [600 MHz, acetone-d6, ppm].

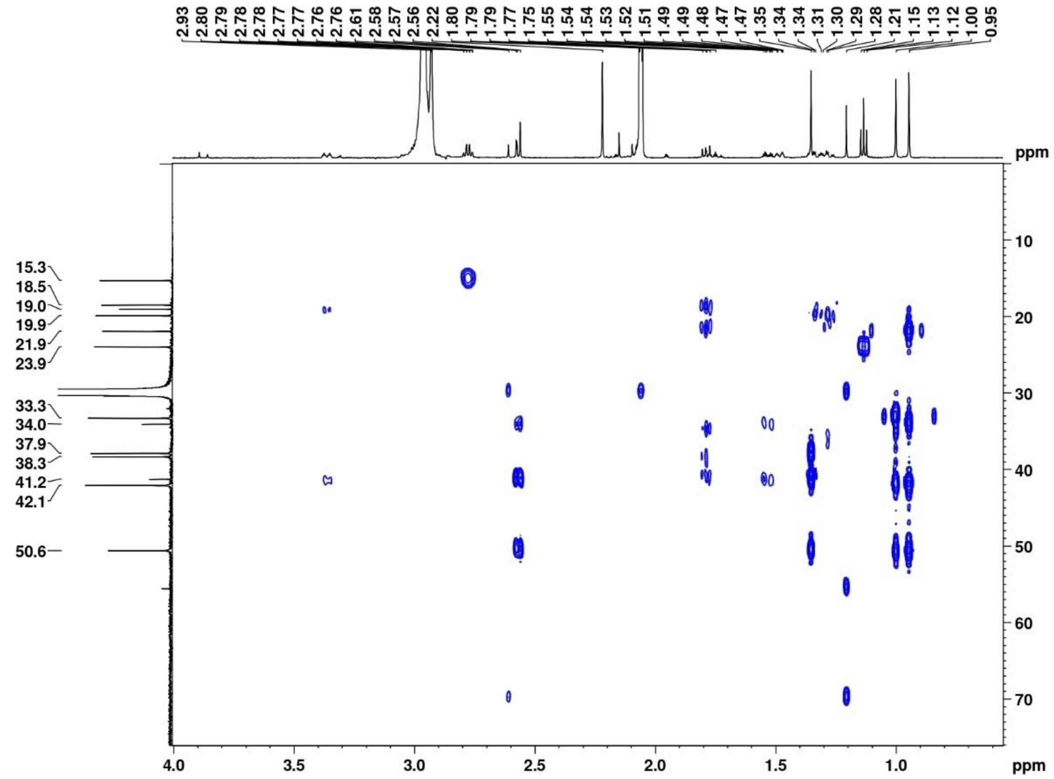

Supplementary Figure 94. HMBC NMR spectrum of 11-hydroxy-cleisthantan-8,11,13-trien-7-one **(5)** [600 MHz, acetone-d6, ppm].

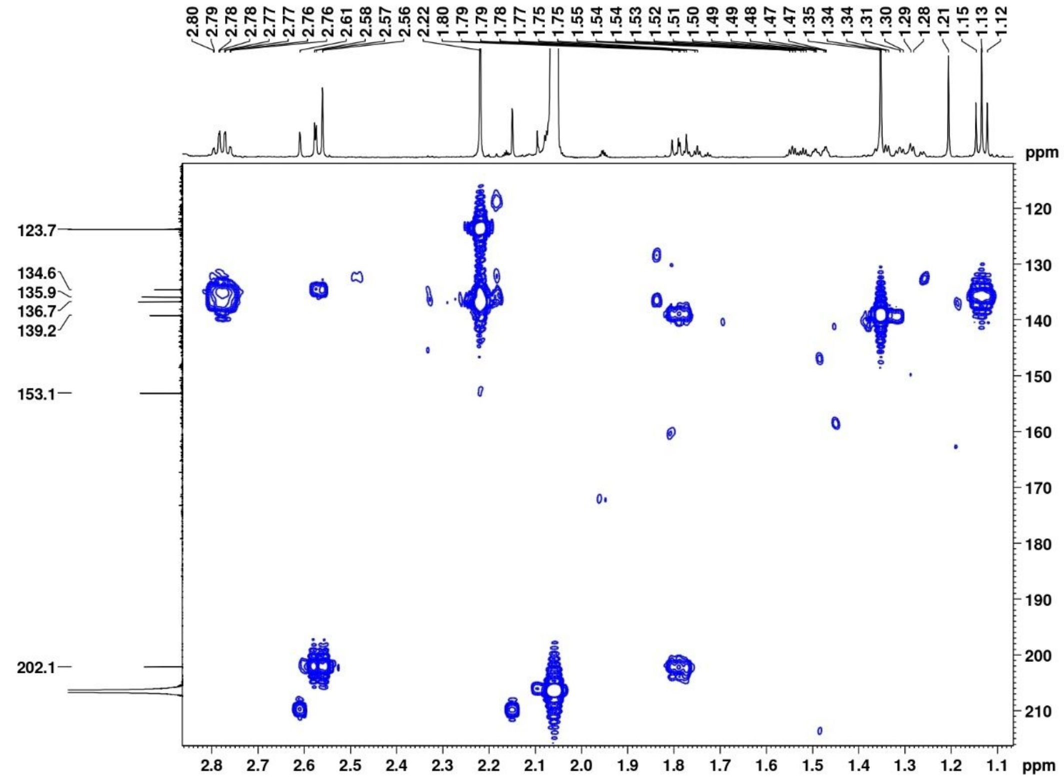

Supplementary Figure 95. HMBC NMR spectrum of 11-hydroxy-cleisthantan-8,11,13-trien-7-one **(5)** [600 MHz, acetone-d6, ppm].

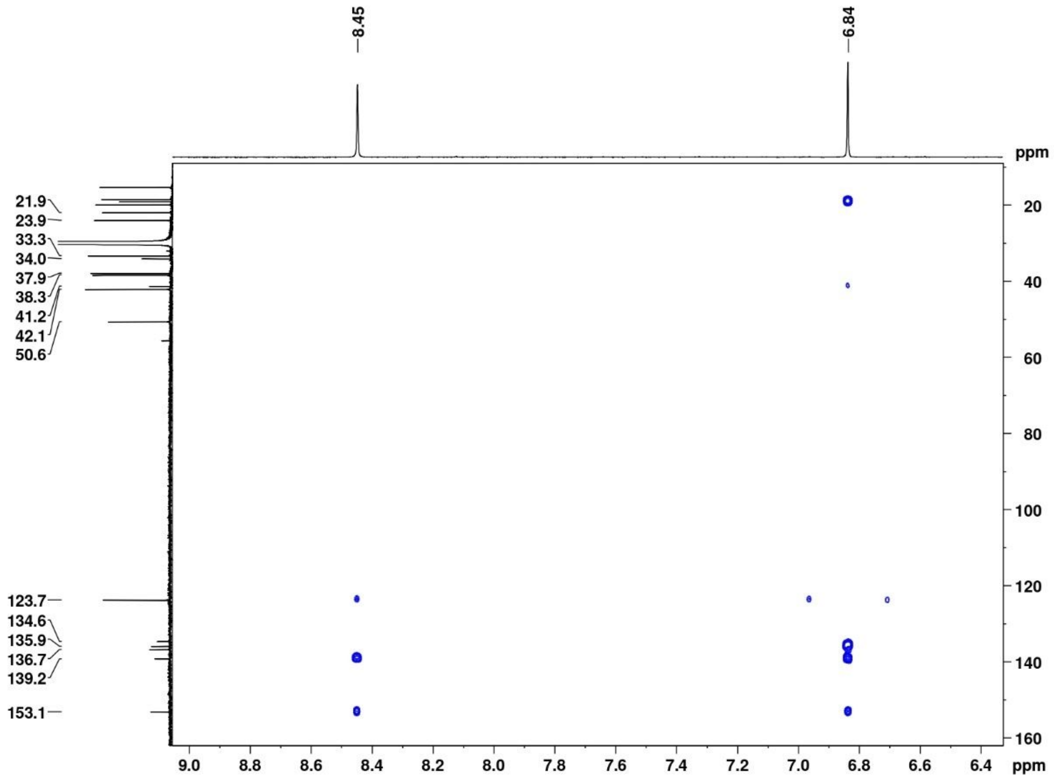

Supplementary Figure 96. HMBC NMR spectrum of 11-hydroxy-cleisthantan-8,11,13-trien-7-one **(5)** [600 MHz, acetone-d6, ppm].

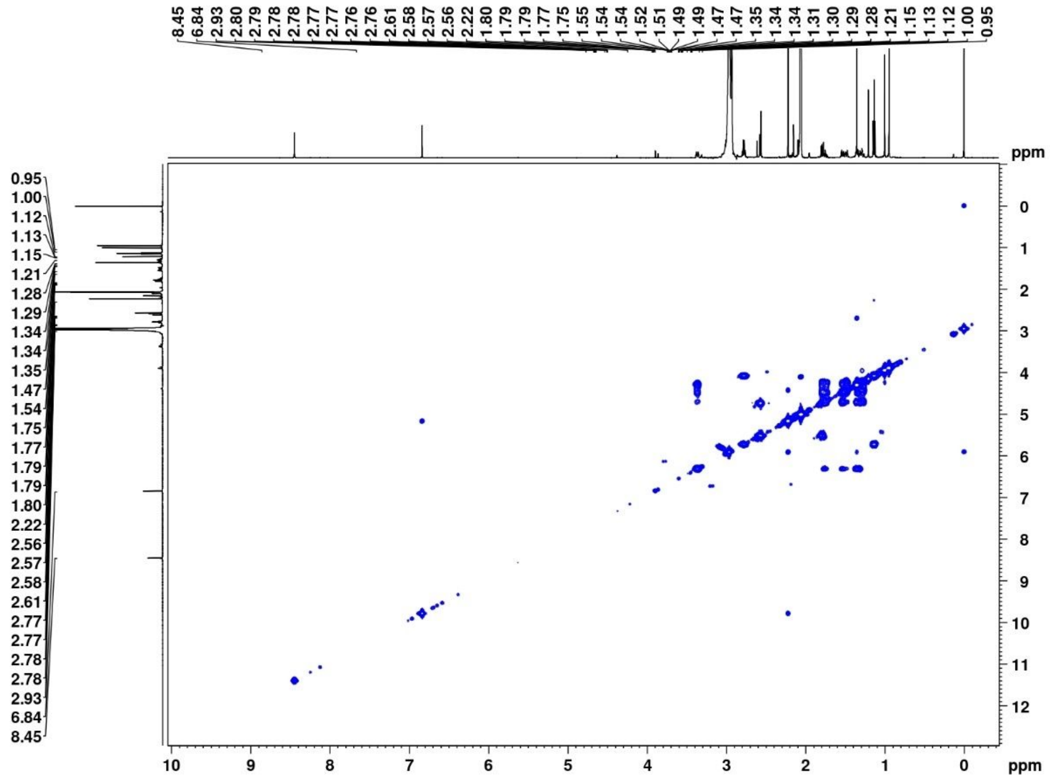

Supplementary Figure 97. COSY NMR spectrum of 11-hydroxy-cleisthantan-8,11,13-trien-7-one **(5)** [600 MHz, acetone-d6, ppm].

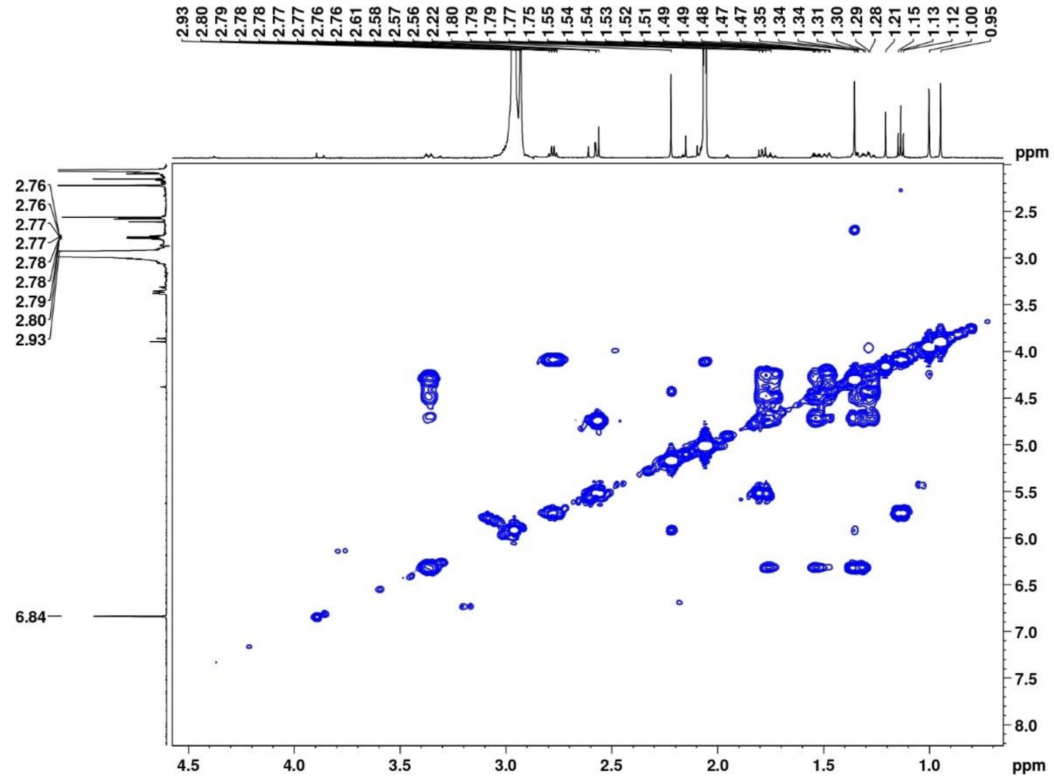

Supplementary Figure 98. COSY NMR spectrum of 11-hydroxy-cleisthantan-8,11,13-trien-7-one **(5)** [600 MHz, acetone-d6, ppm].

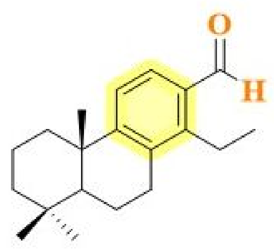

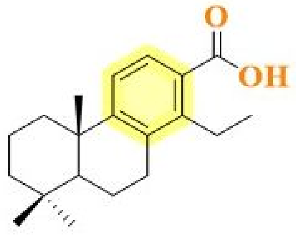

6 **7**

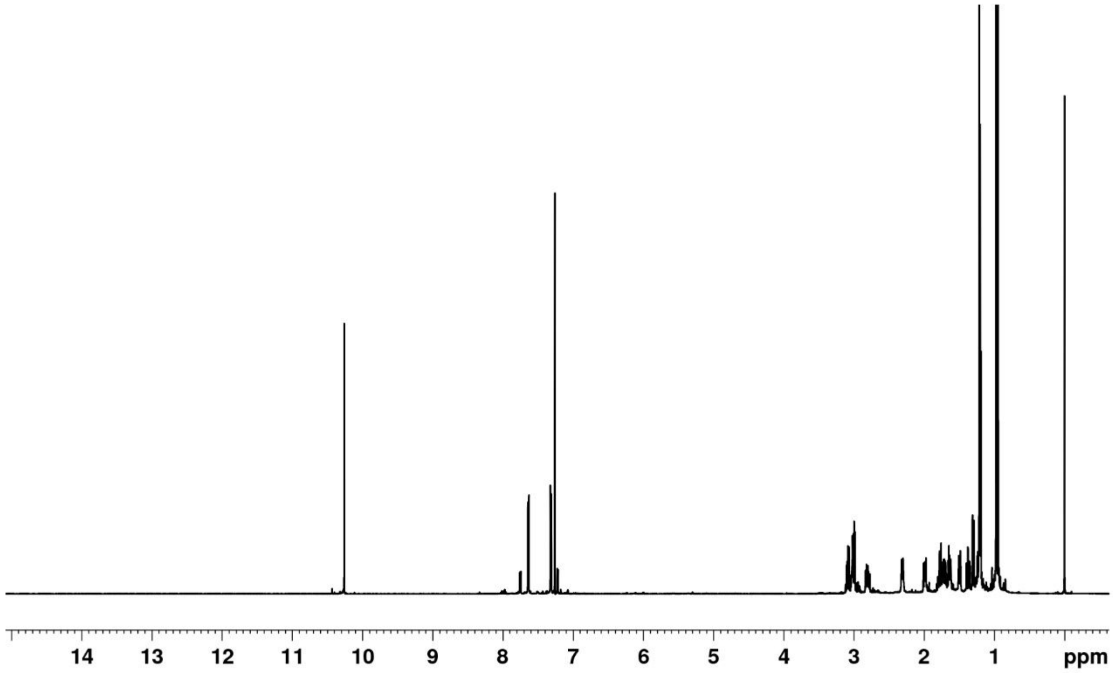

Supplementary Figure 99. ^{1}H NMR spectrum of Veadeiral **(6)** (major) and Veadeiroic Acid **(7)** (minor) [600 MHz, $CDCl_3$, ppm].

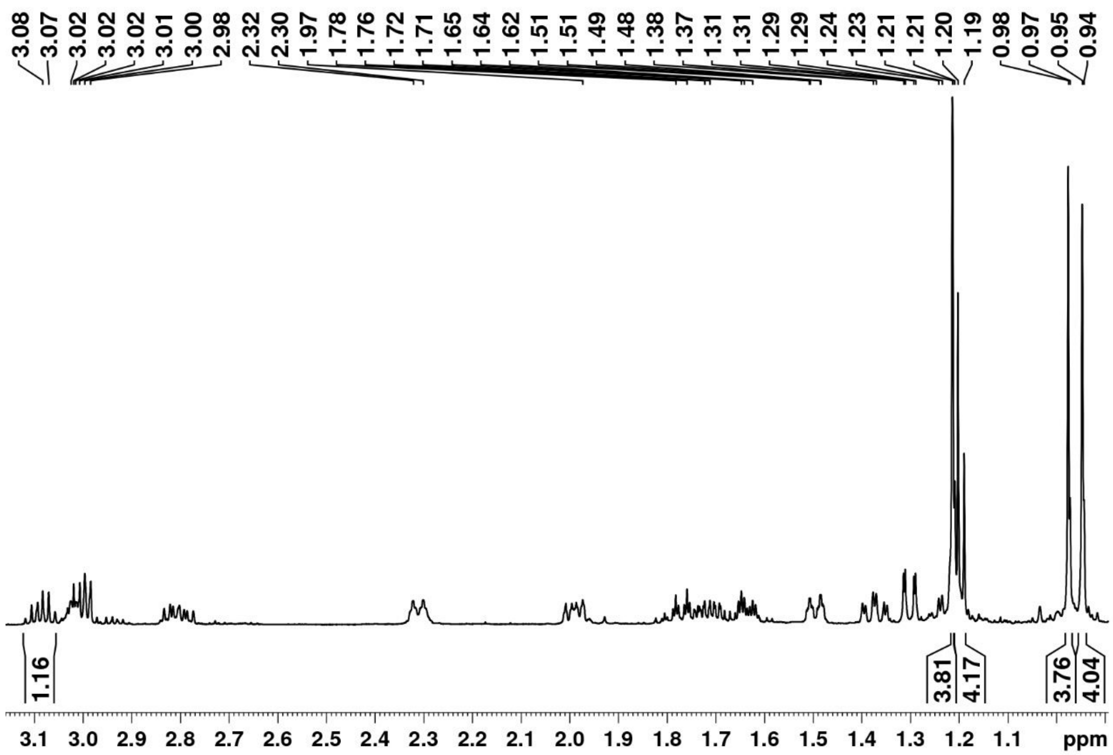

Supplementary Figure 100. ^{1}H NMR spectrum of Veadeiral **(6)** (major) and Veadeiroic Acid **(7)** (minor) [600 MHz, $CDCl_3$, ppm].

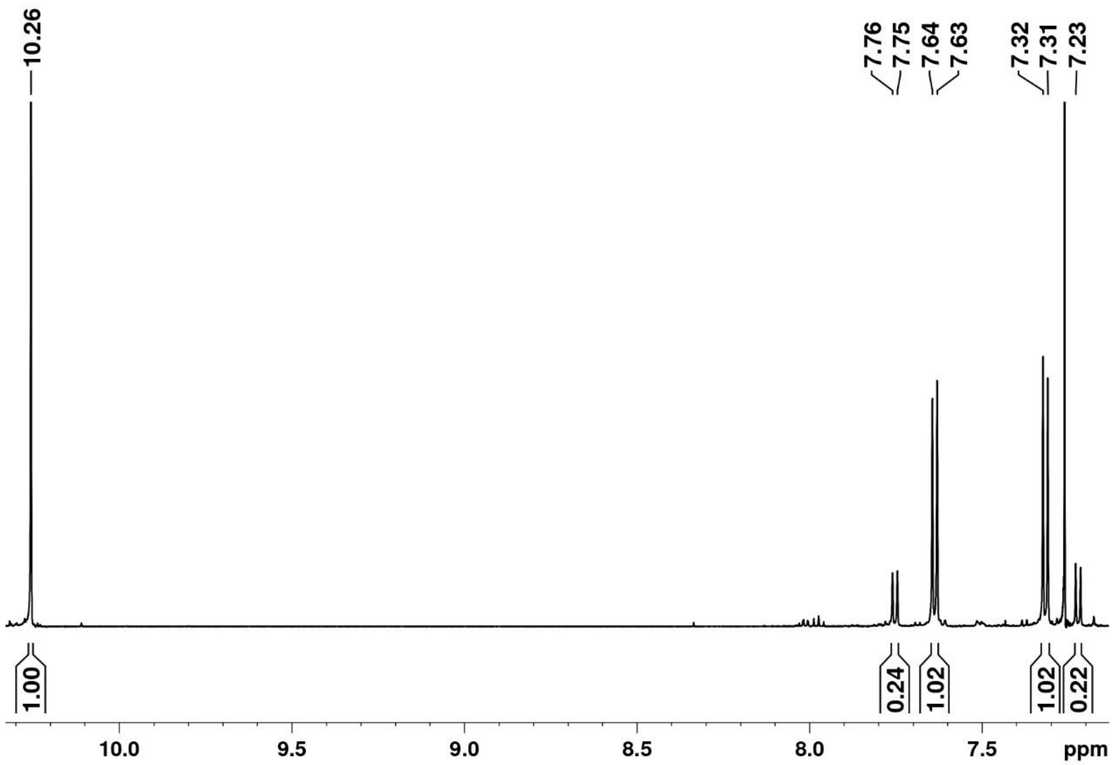

Supplementary Figure 101. ^{1}H NMR spectrum of Veadeiral **(6)** (major) and Veadeiroic Acid **(7)** (minor) [600 MHz, $CDCl_3$, ppm].

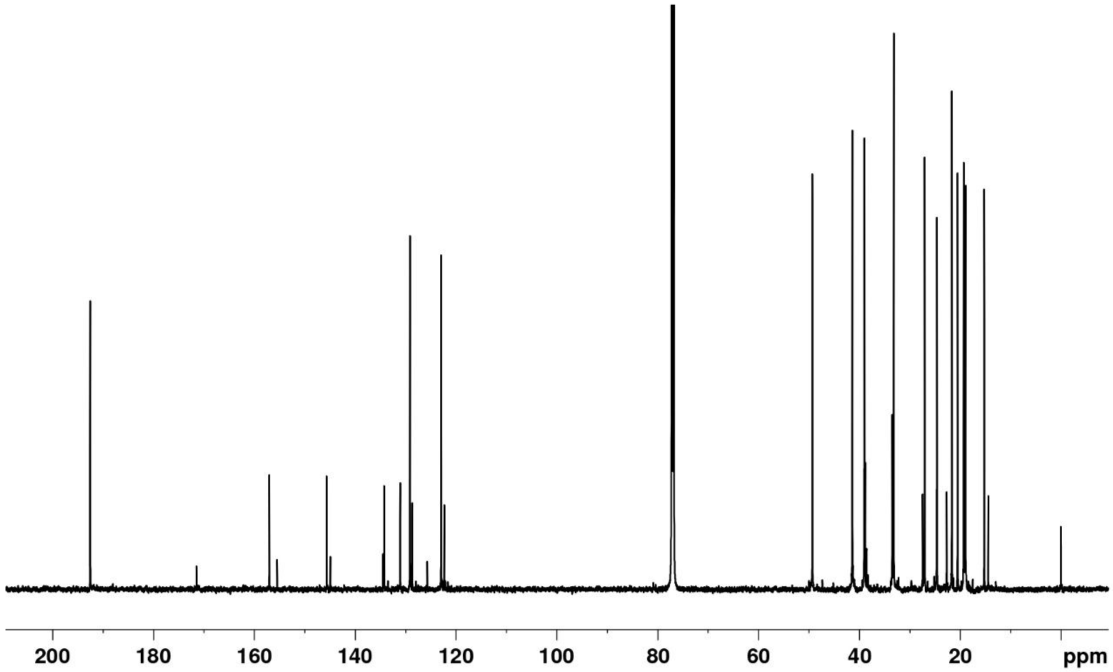

Supplementary Figure 102. ^{13}C NMR spectrum of Veadeiral **(6)** (major) and Veadeiroic Acid **(7)** (minor) [150 MHz, $CDCl_3$, ppm].

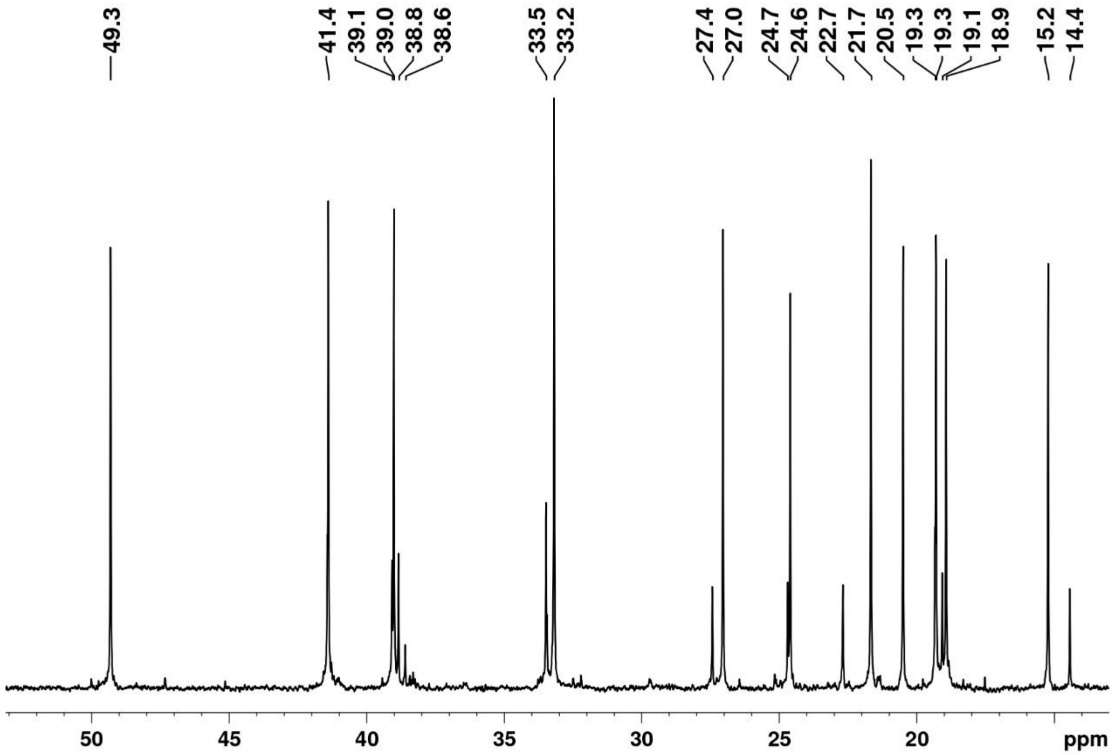

Supplementary Figure 103. ^{13}C NMR spectrum of Veadeiral **(6)** (major) and Veadeiroic Acid **(7)** (minor) [150 MHz, $CDCl_3$, ppm].

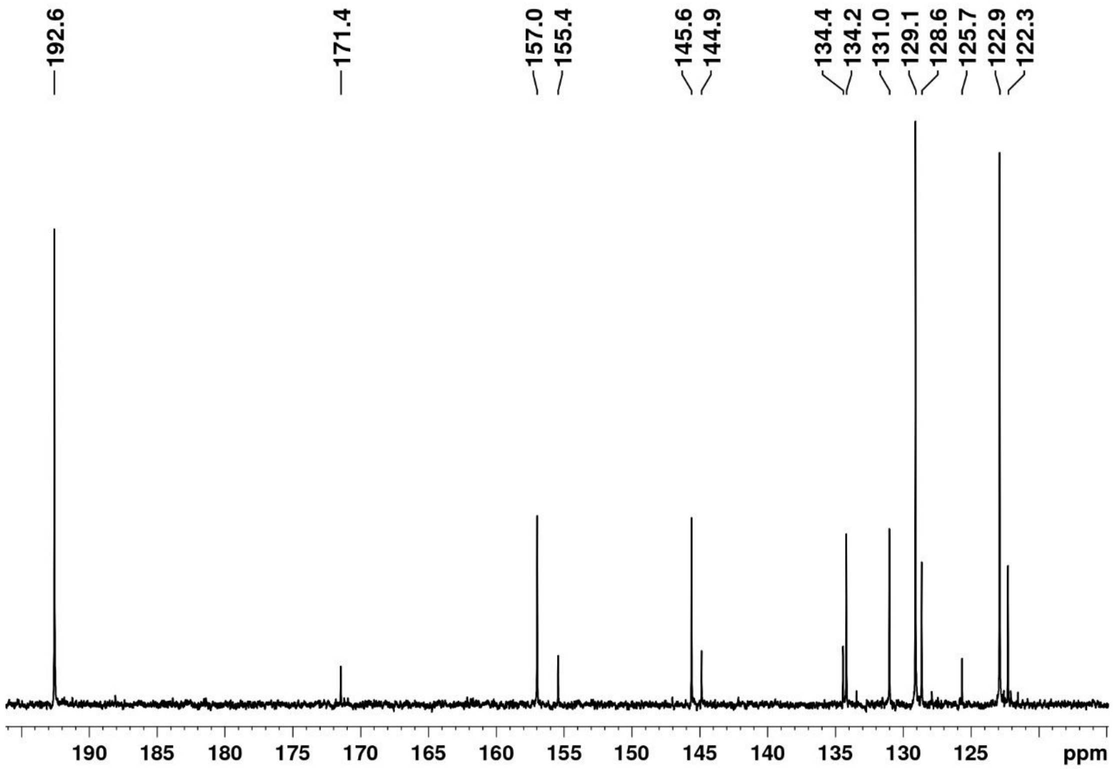

Supplementary Figure 104. ^{13}C NMR spectrum of Veadeiral **(6)** (major) and Veadeiroic Acid **(7)** (minor) [150 MHz, $CDCl_3$, ppm].

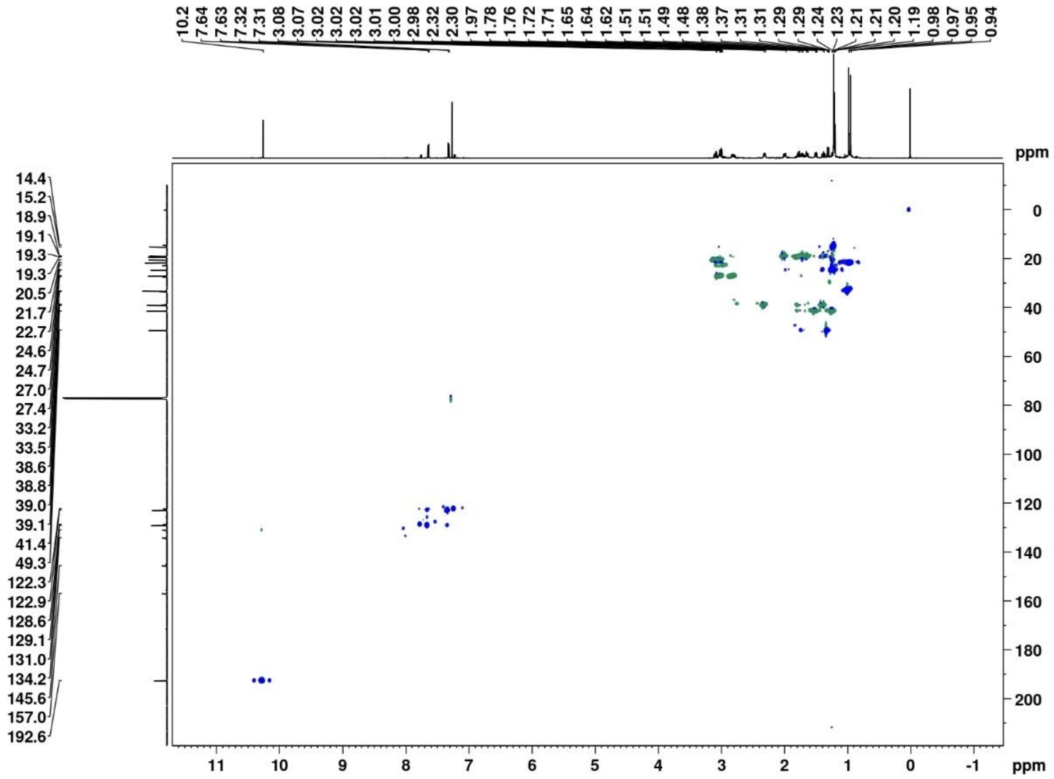

Supplementary Figure 105. HSQC NMR spectrum of Veadeiral **(6)** (major) and Veadeiroic Acid **(7)** (minor) [600 MHz, $CDCl_3$, ppm].

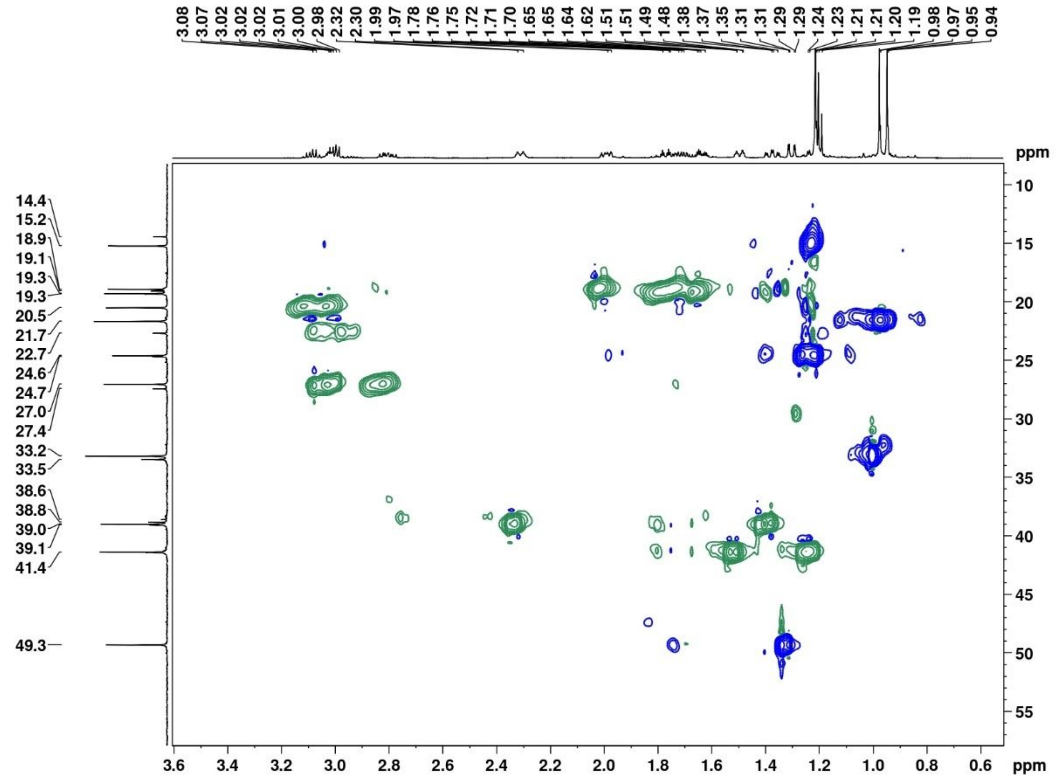

Supplementary Figure 106. HSQC NMR spectrum of Veadeiral **(6)** (major) and Veadeiroic Acid **(7)** (minor) [600 MHz, $CDCl_3$, ppm].

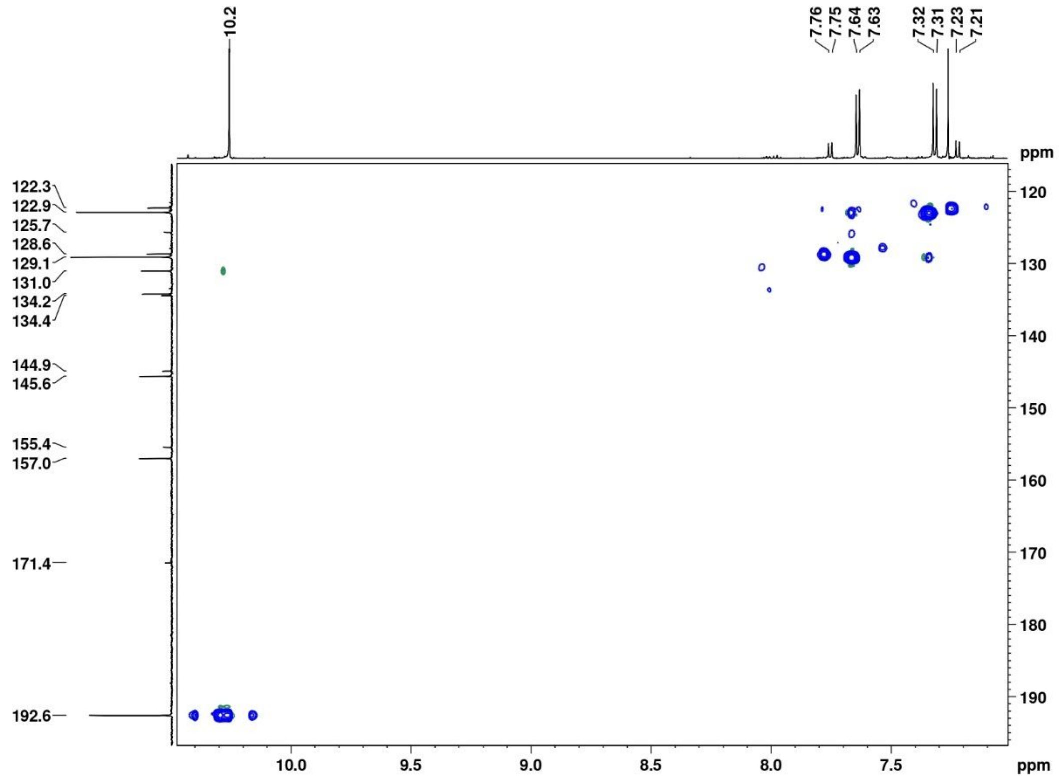

Supplementary Figure 107. HSQC NMR spectrum of Veadeiral **(6)** (major) and Veadeiroic Acid **(7)** (minor) [600 MHz, $CDCl_3$, ppm].

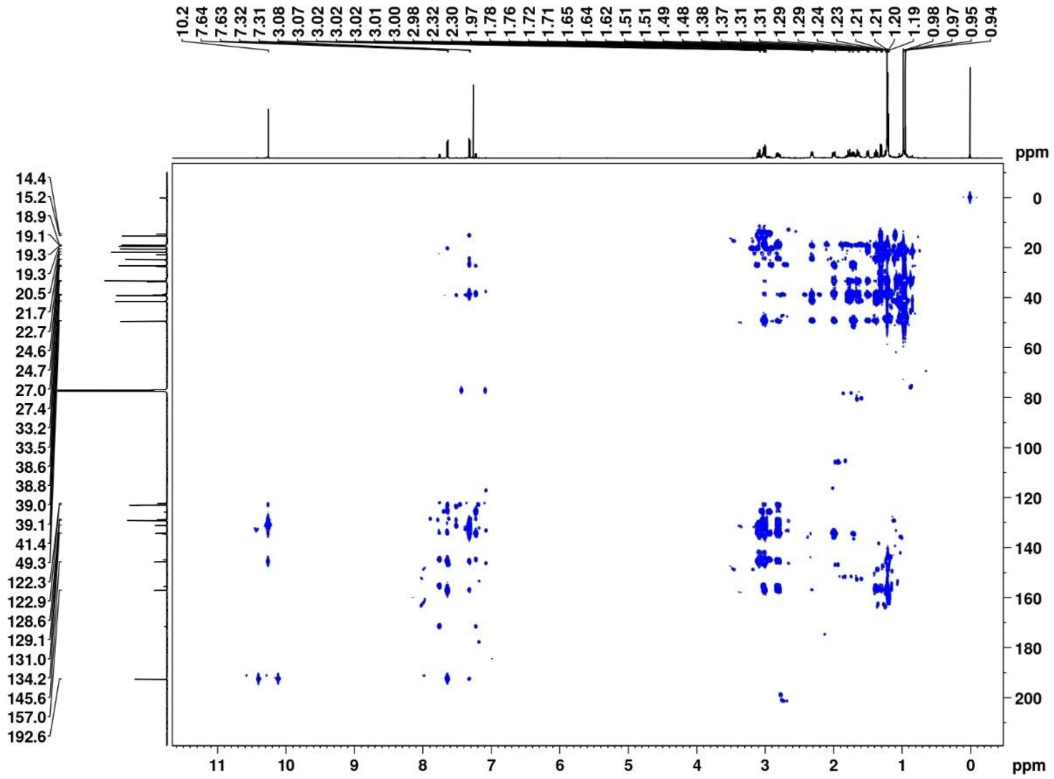

Supplementary Figure 108. HMBC NMR spectrum of Veadeiral **(6)** (major) and Veadeiroic Acid **(7)** (minor) [600 MHz, $CDCl_3$, ppm].

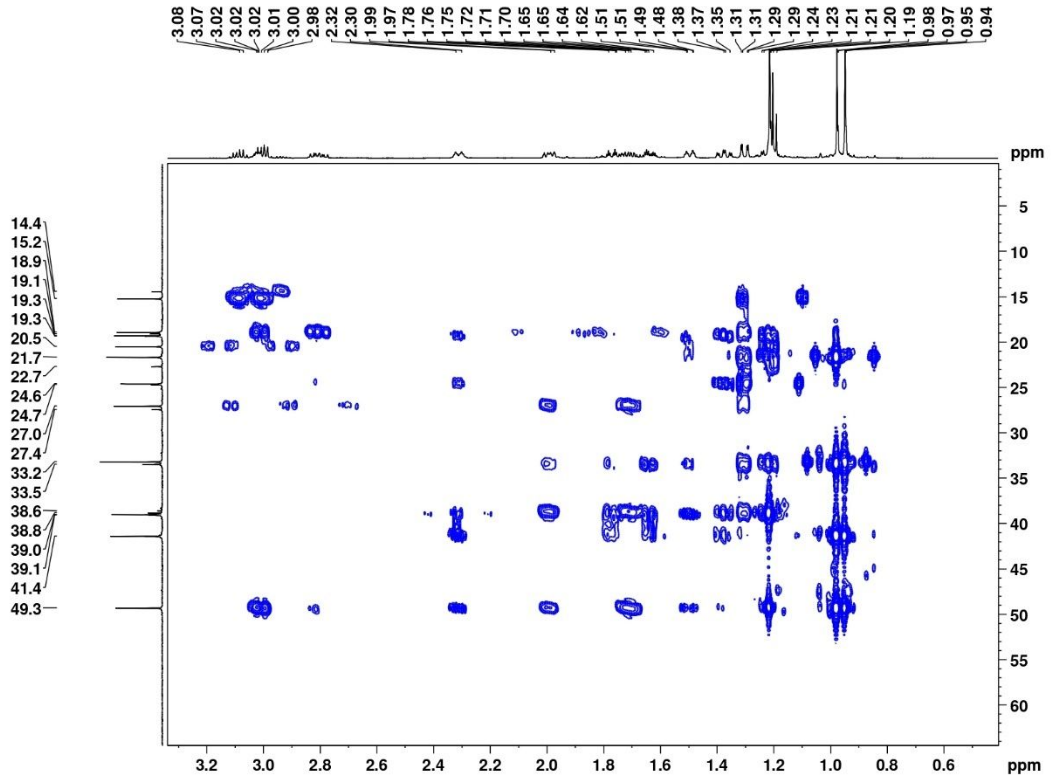

Supplementary Figure 109. HMBC NMR spectrum of Veadeiral **(6)** (major) and Veadeiroic Acid **(7)** (minor) [600 MHz, $CDCl_3$, ppm].

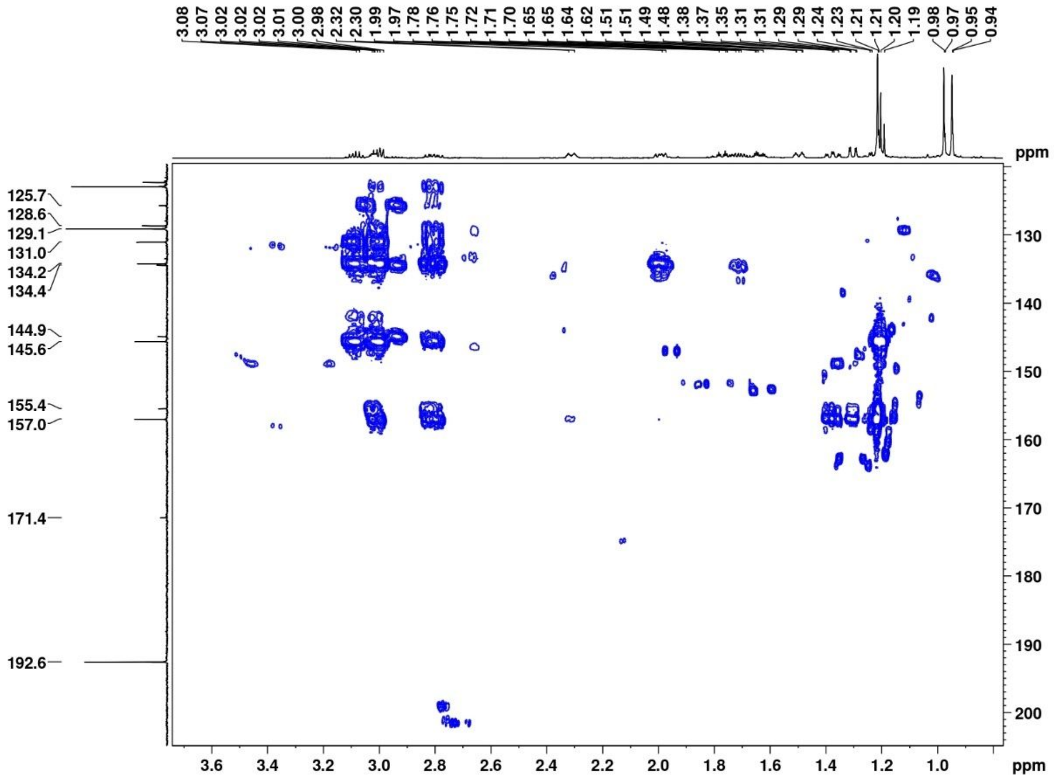

Supplementary Figure 110. HMBC NMR spectrum of Veadeiral **(6)** (major) and Veadeiroic Acid **(7)** (minor) [600 MHz, $CDCl_3$, ppm].

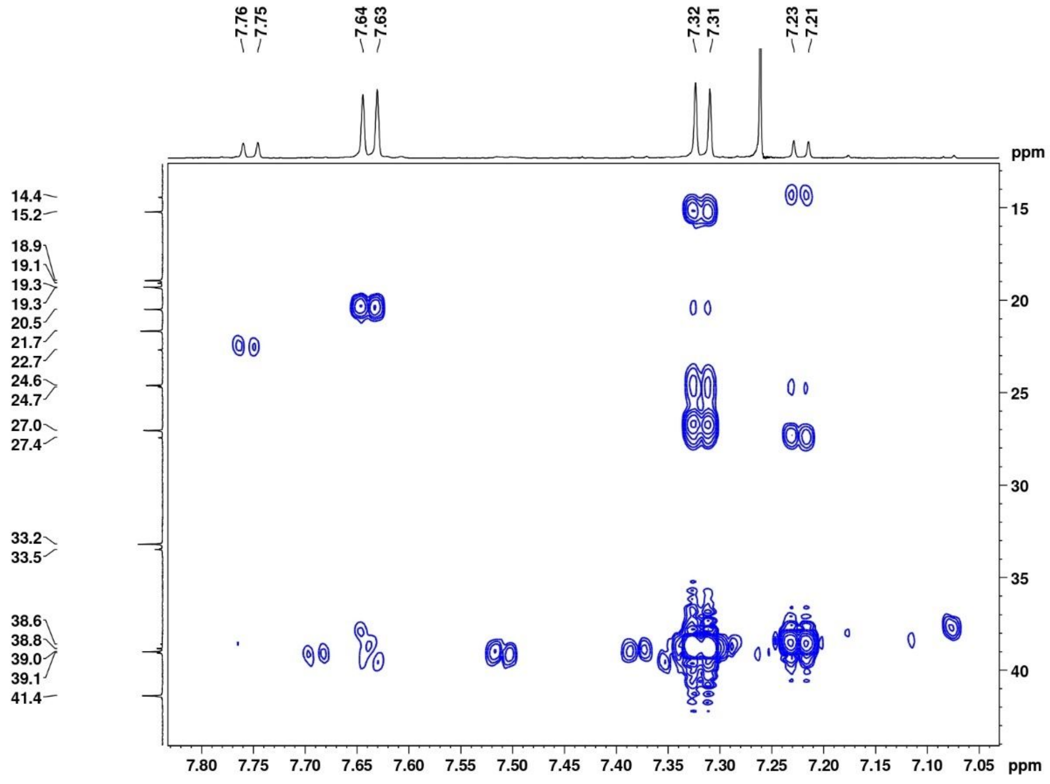

Supplementary Figure 111. HMBC NMR spectrum of Veadeiral **(6)** (major) and Veadeiroic Acid **(7)** (minor) [600 MHz, $CDCl_3$, ppm].

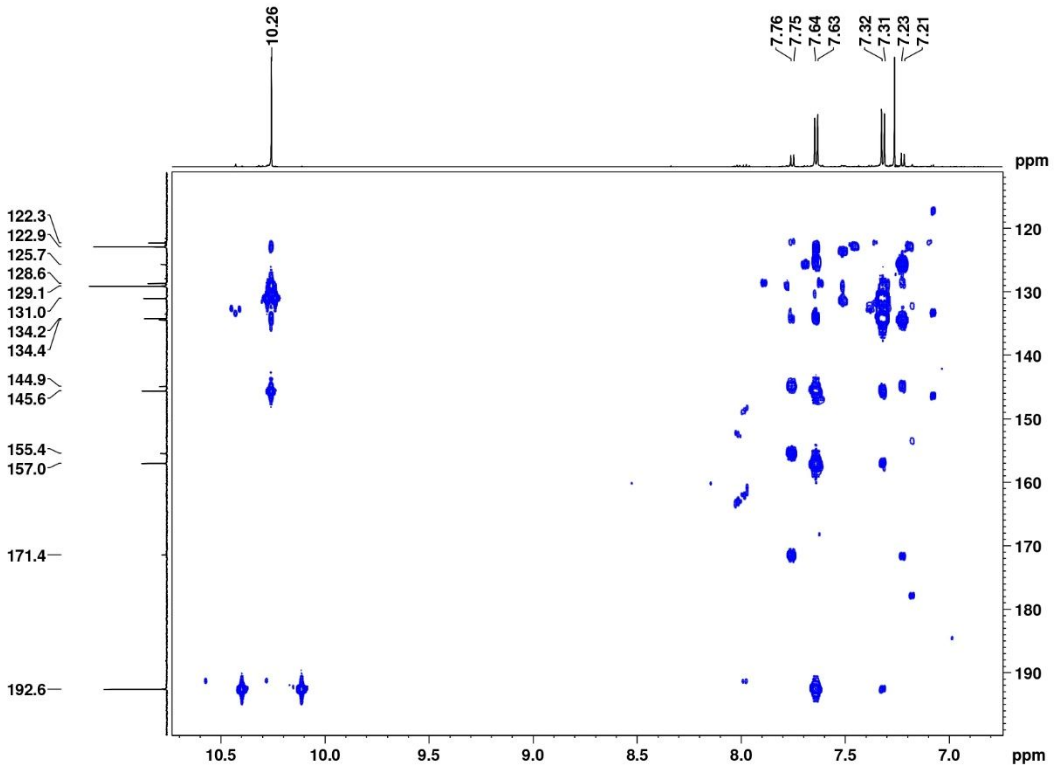

Supplementary Figure 112. HMBC NMR spectrum of Veadeiral **(6)** (major) and Veadeiroic Acid **(7)** (minor) [600 MHz, $CDCl_3$, ppm].

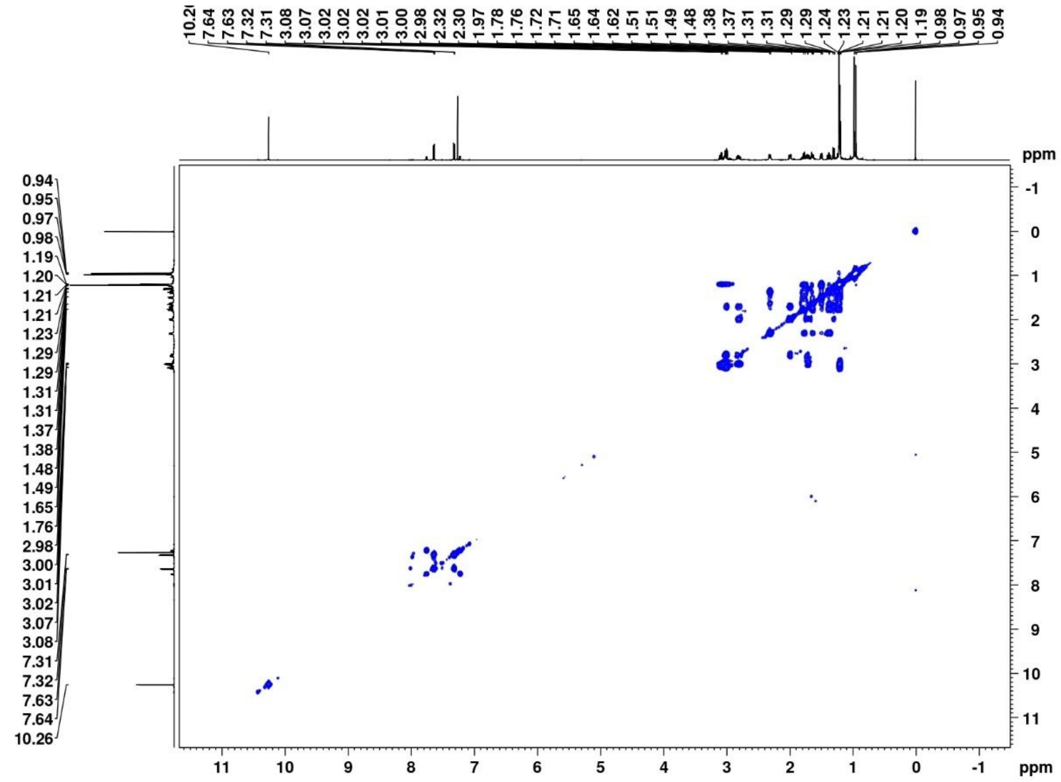

Supplementary Figure 113. COSY NMR spectrum of Veadeiral **(6)** (major) and Veadeiroic Acid **(7)** (minor) [600 MHz, $CDCl_3$, ppm].

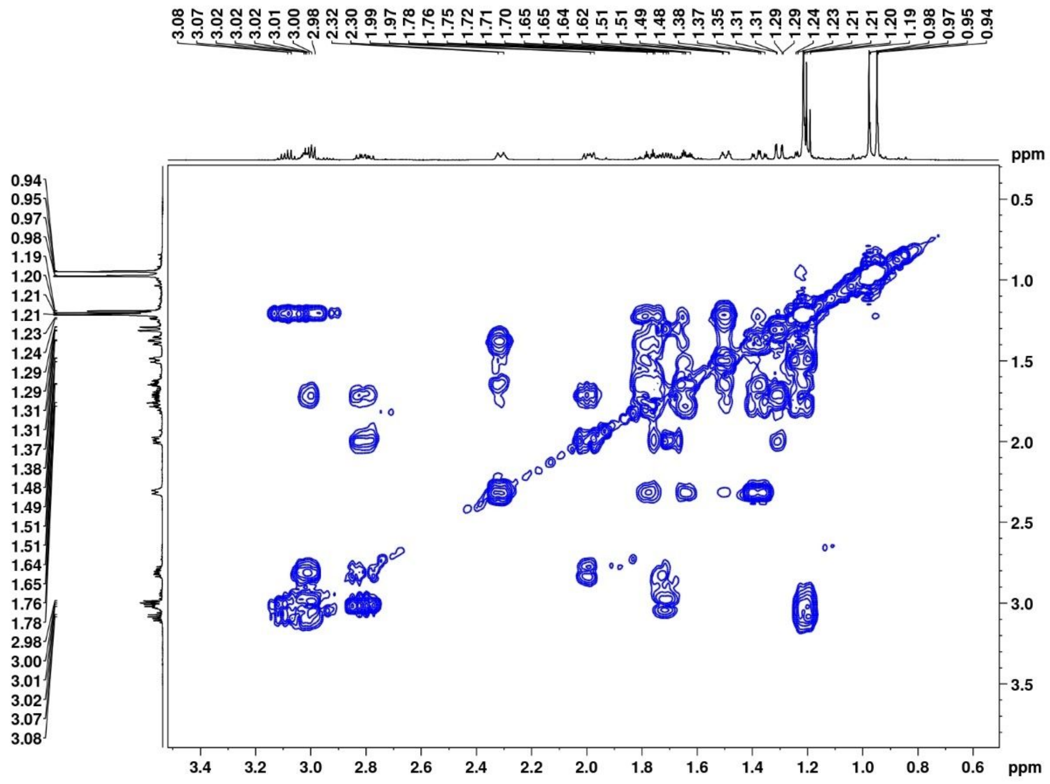

Supplementary Figure 114. COSY NMR spectrum of Veadeiral **(6)** (major) and Veadeiroic Acid **(7)** (minor) [600 MHz, $CDCl_3$, ppm].

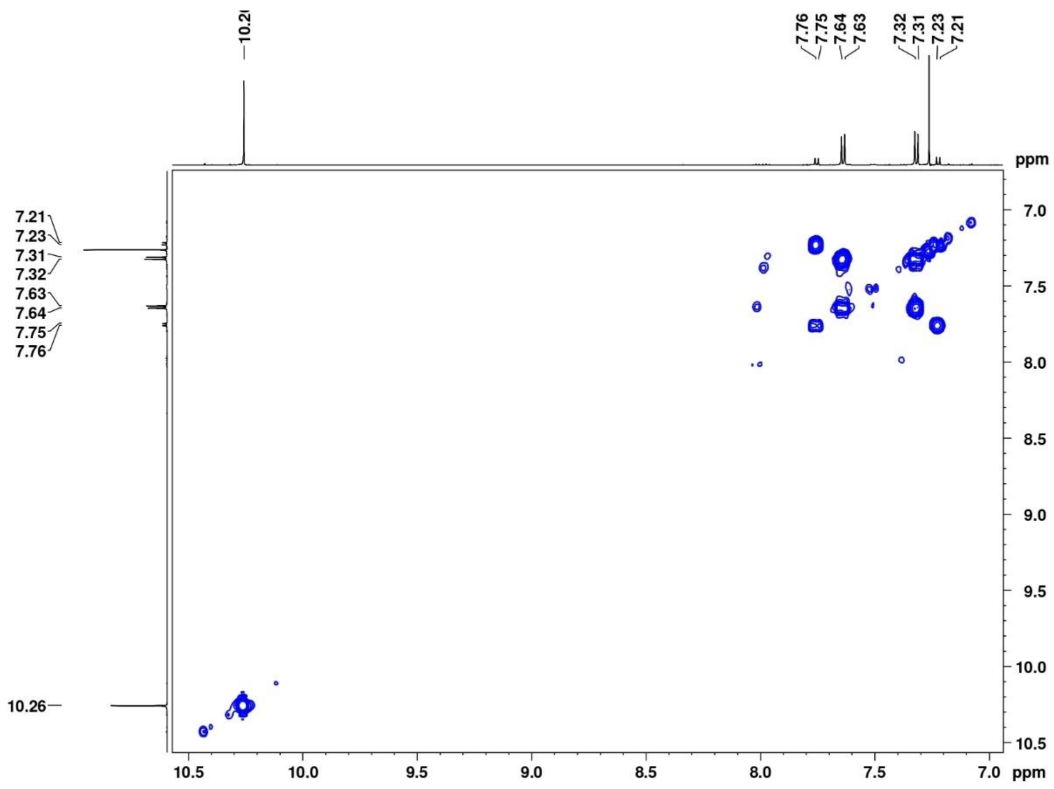

Supplementary Figure 115. COSY NMR spectrum of Veadeiral **(6)** (major) and Veadeiroic Acid **(7)** (minor) [600 MHz, $CDCl_3$, ppm].

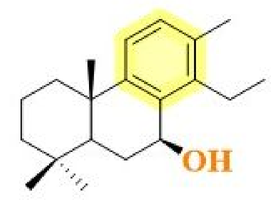

8

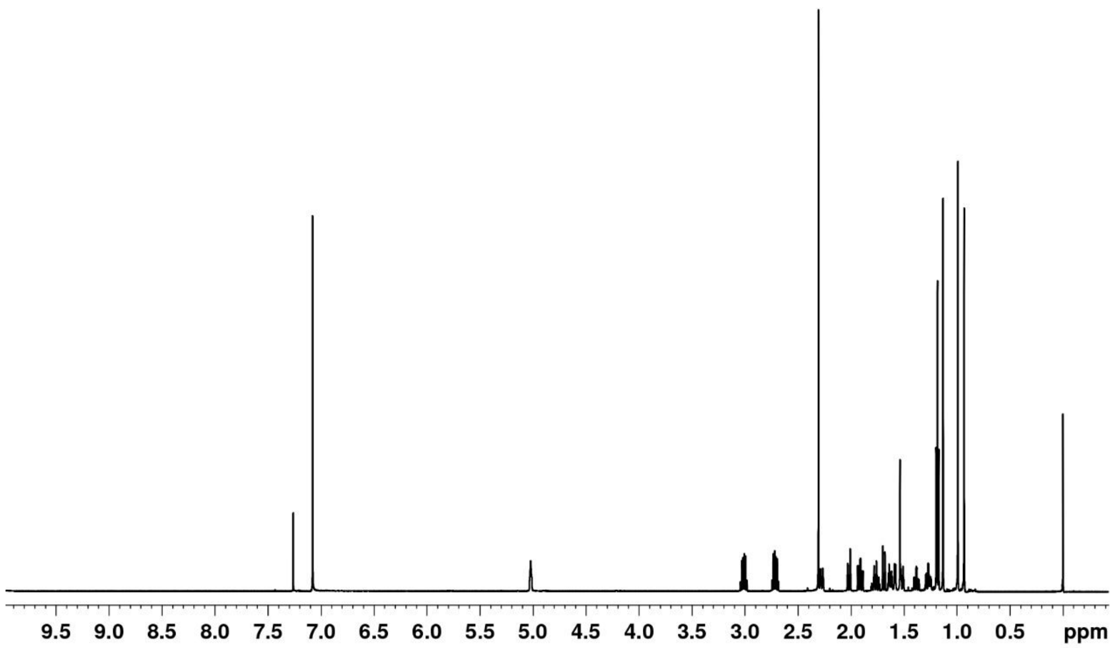

Supplementary Figure 116. ^{1}H NMR spectrum of 7β-hydroxy-clesitantha-8,11,13-triene **(8)** [600 MHz, $CDCl_3$, ppm].

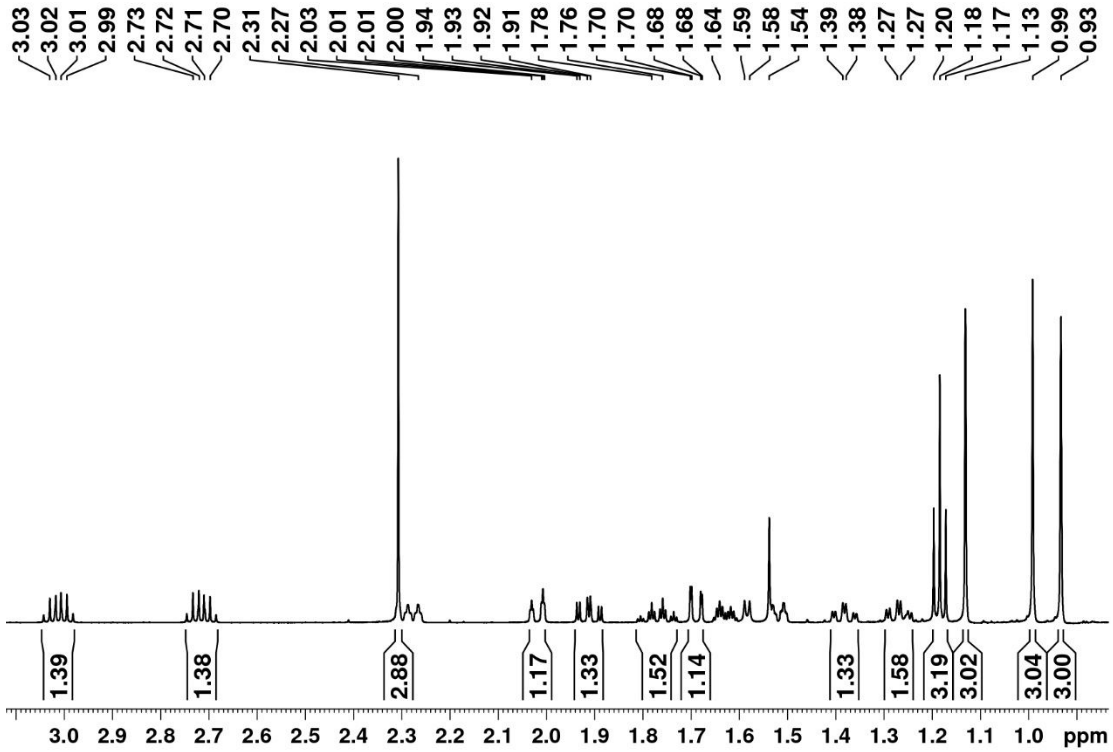

Supplementary Figure 117. ^{1}H NMR spectrum of 7β-hydroxy-clesitantha-8,11,13-triene **(8)** [600 MHz, $CDCl_3$, ppm].

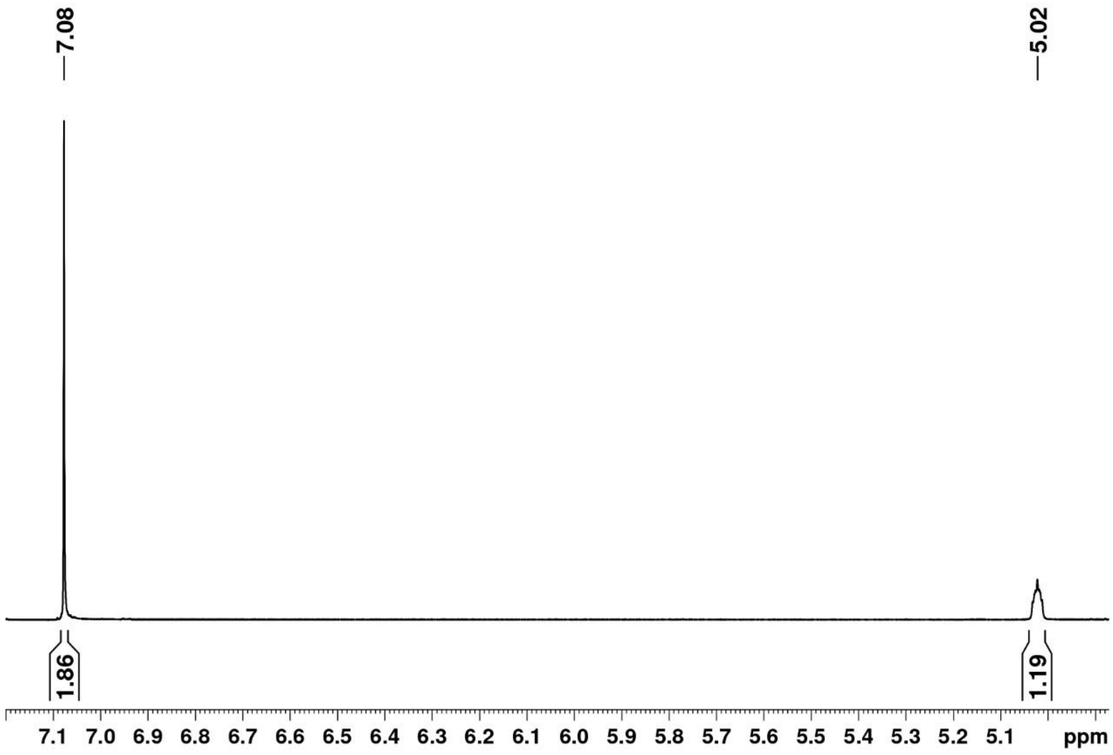

Supplementary Figure 118. ^{1}H NMR spectrum of 7β-hydroxy-clesitantha-8,11,13-triene **(8)** [600 MHz, $CDCl_3$, ppm].

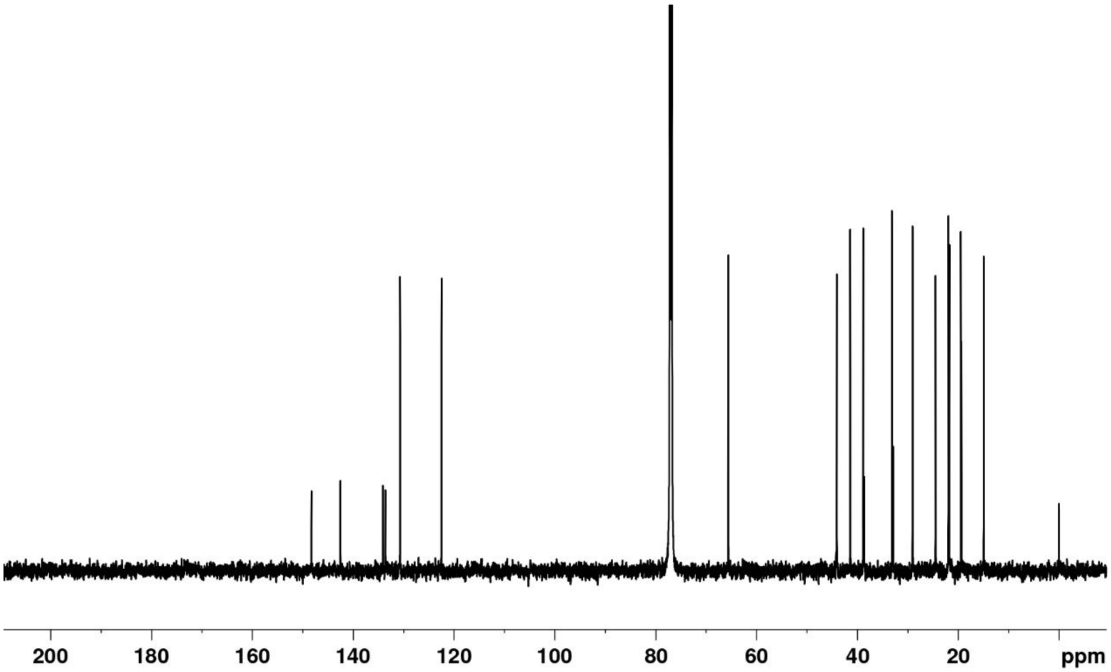

Supplementary Figure 119. ^{13}C NMR spectrum of 7β-hydroxy-clesitantha-8,11,13-triene **(8)** [150 MHz, $CDCl_3$, ppm].

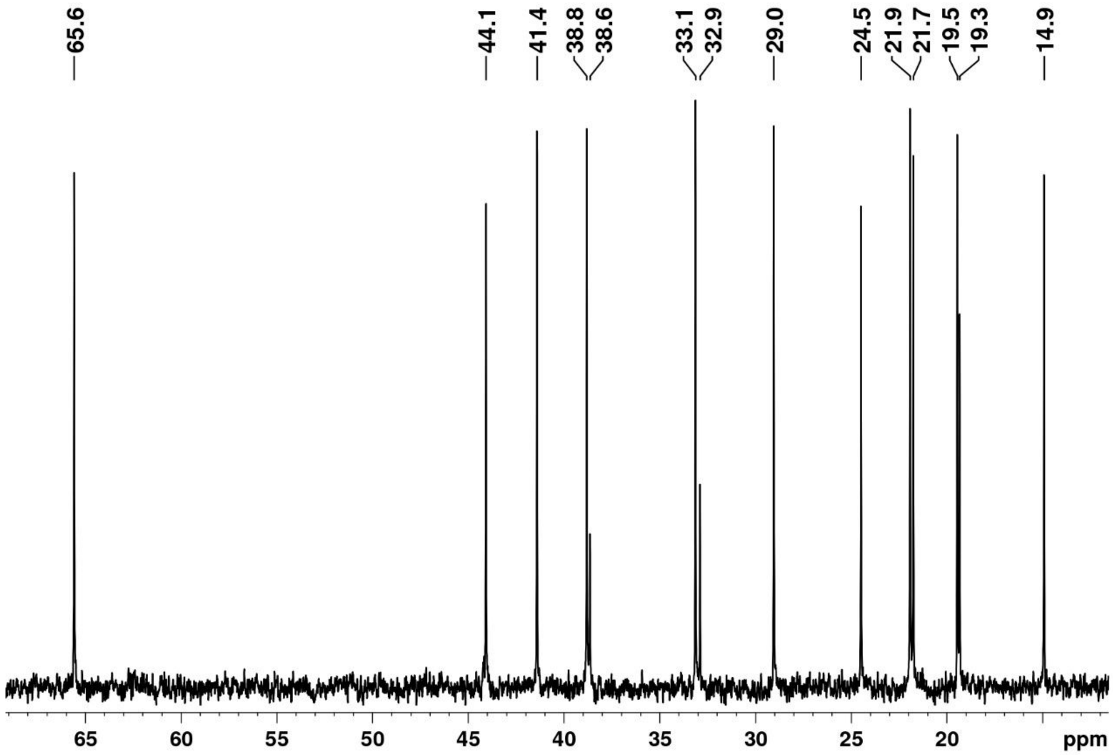

Supplementary Figure 120. ^{13}C NMR spectrum of 7β-hydroxy-clesitantha-8,11,13-triene **(8)** [150 MHz, $CDCl_3$, ppm].

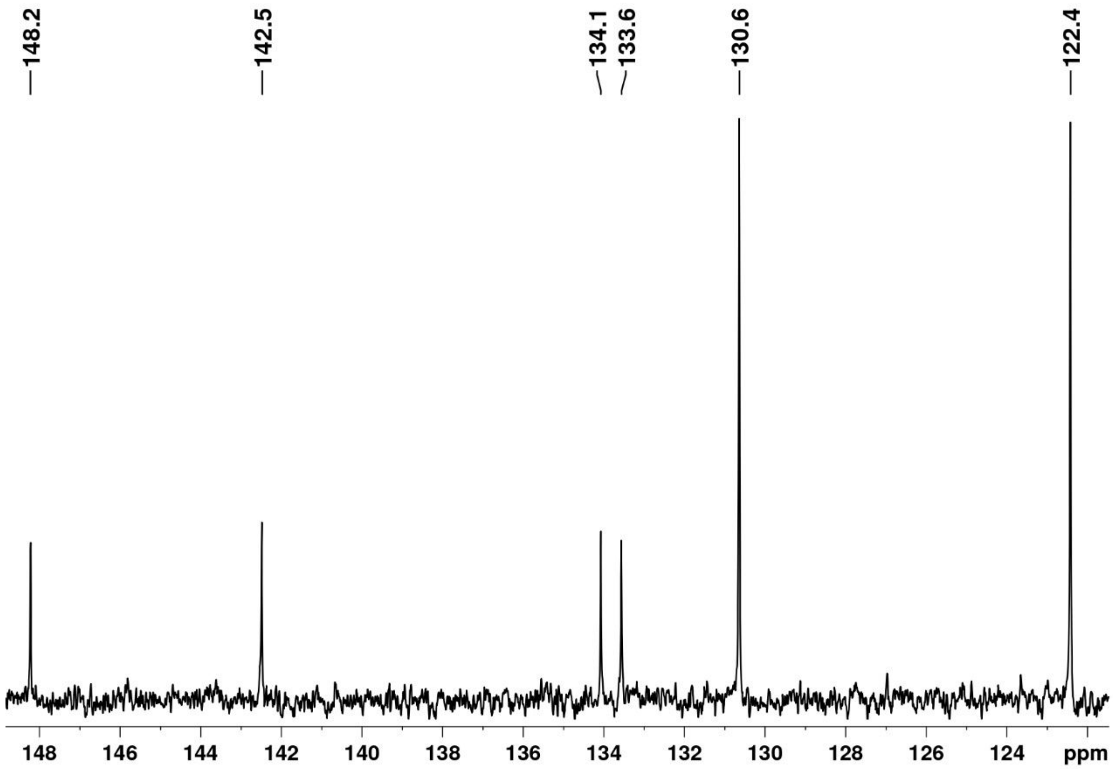

Supplementary Figure 121. ^{13}C NMR spectrum of 7β-hydroxy-clesitantha-8,11,13-triene **(8)** [150 MHz, $CDCl_3$, ppm].

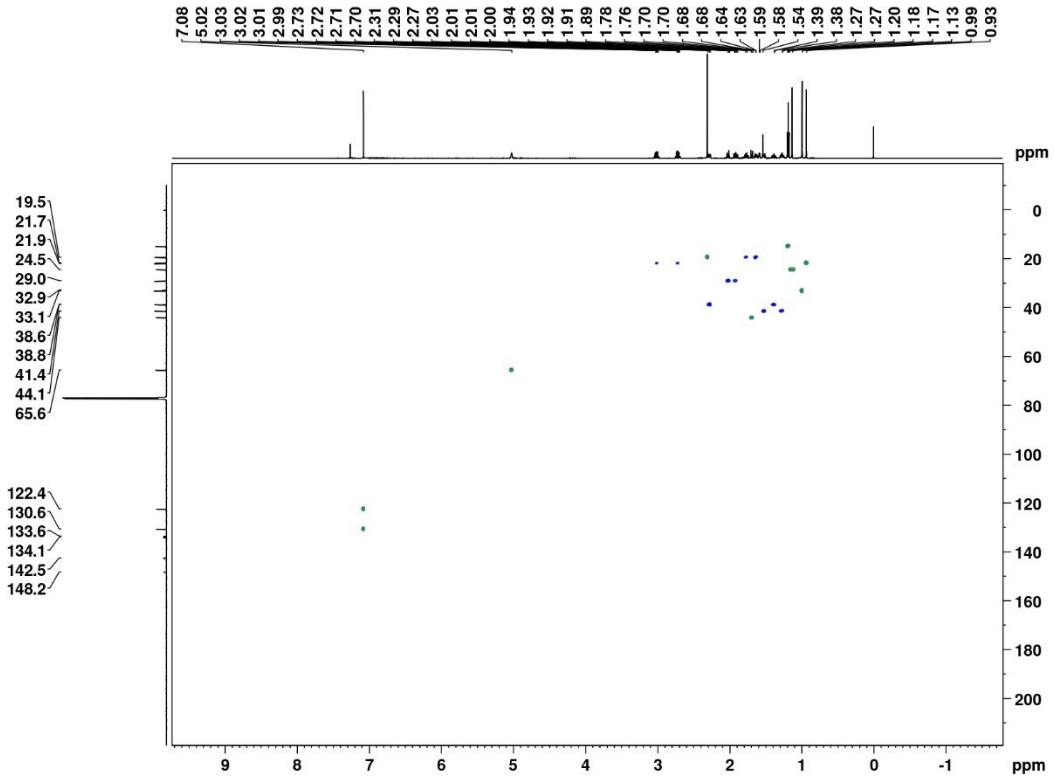

Supplementary Figure 122. HSQC NMR spectrum of 7β-hydroxy-clesitantha-8,11,13-triene **(8)** [600 MHz, $CDCl_3$, ppm].

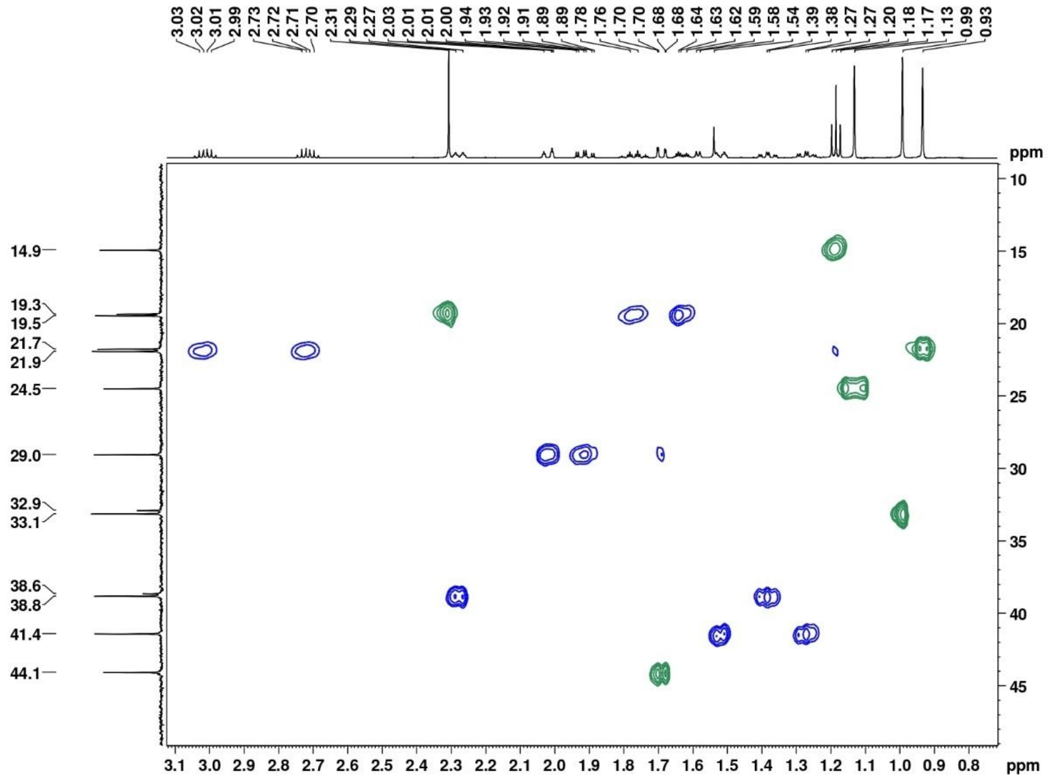

Supplementary Figure 123. HSQC NMR spectrum of 7β-hydroxy-clesitantha-8,11,13-triene **(8)** [600 MHz, $CDCl_3$, ppm].

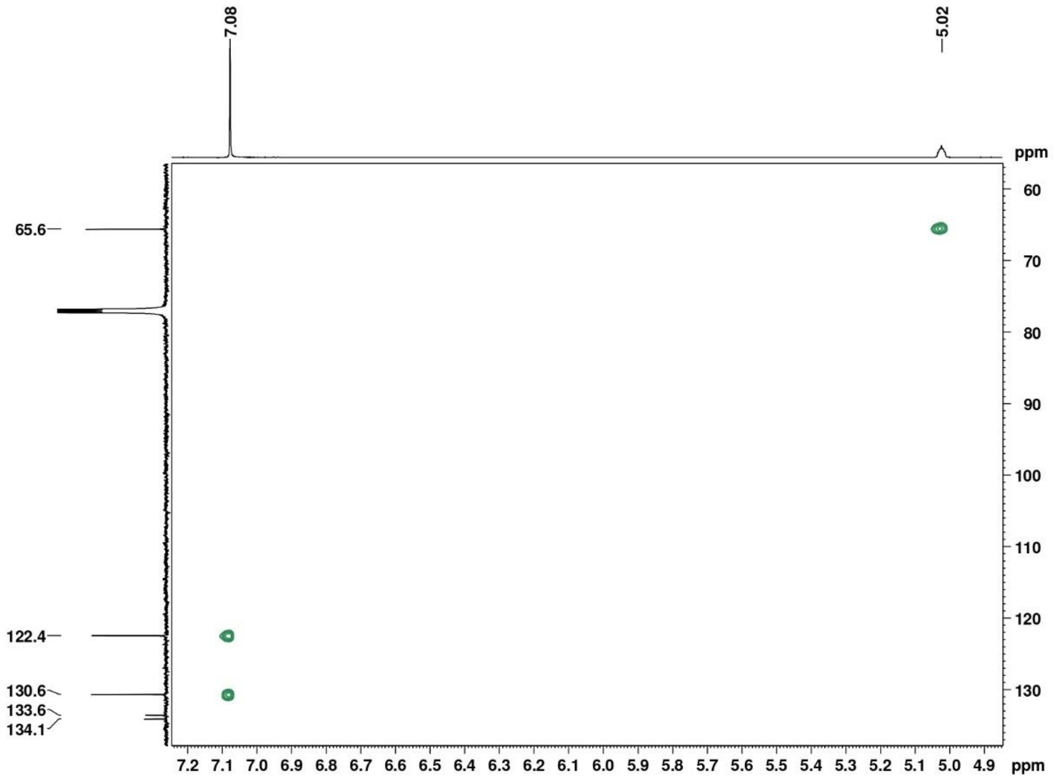

Supplementary Figure 124. HSQC NMR spectrum of 7β-hydroxy-clesitantha-8,11,13-triene **(8)** [600 MHz, $CDCl_3$, ppm].

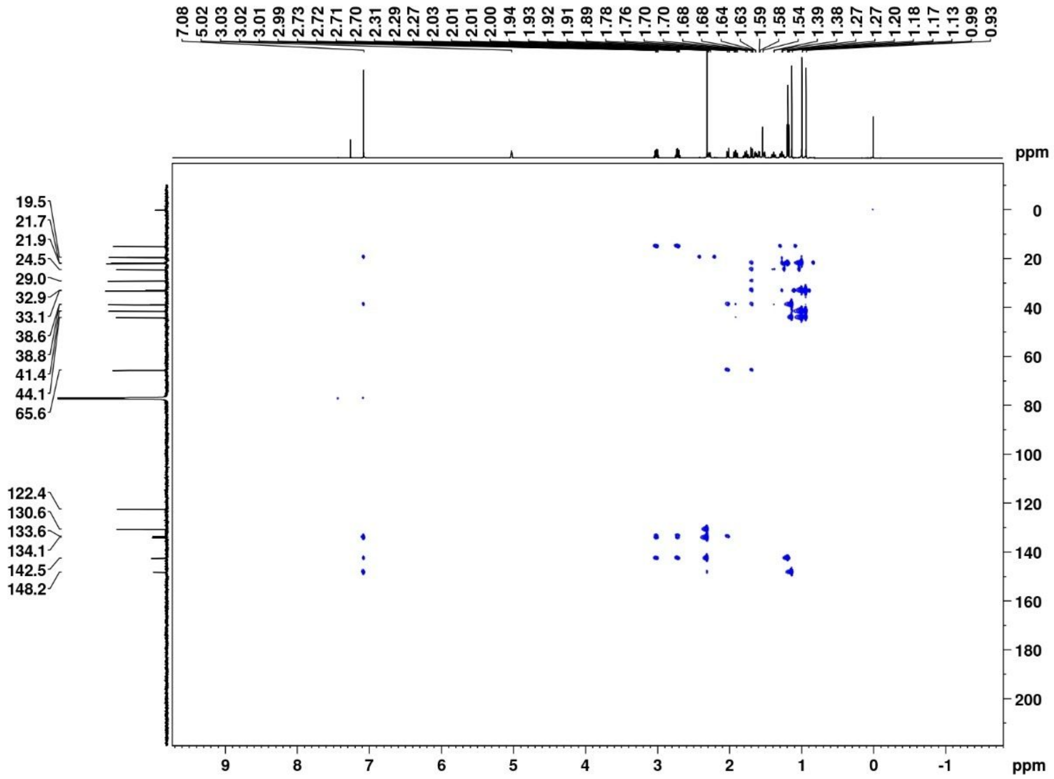

Supplementary Figure 125. HMBC NMR spectrum of 7β-hydroxy-clesitantha-8,11,13-triene **(8)** [600 MHz, $CDCl_3$, ppm].

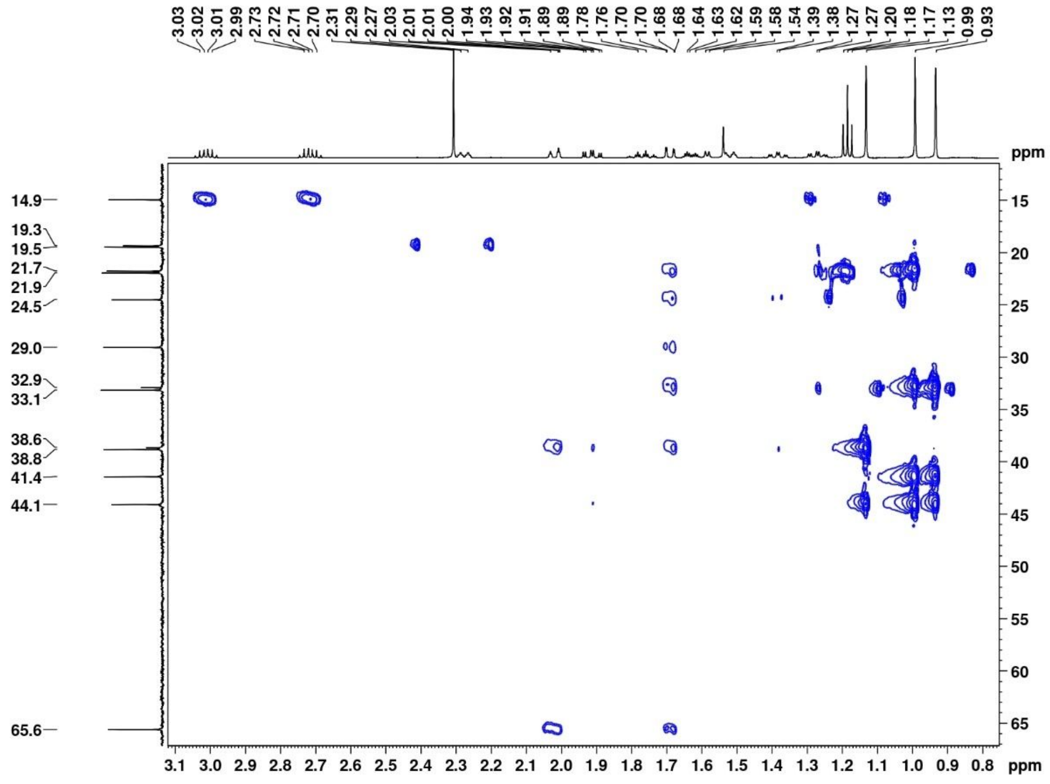

Supplementary Figure 126. HMBC NMR spectrum of 7β-hydroxy-clesitantha-8,11,13-triene **(8)** [600 MHz, $CDCl_3$, ppm].

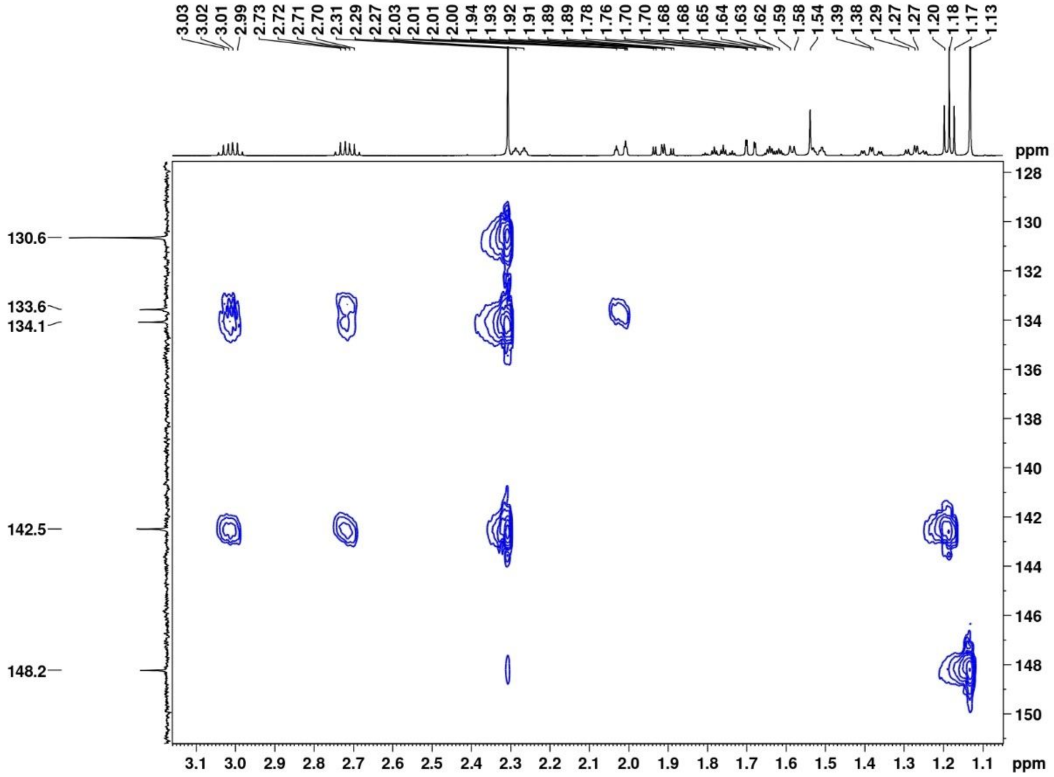

Supplementary Figure 127. HMBC NMR spectrum of 7β-hydroxy-clesitantha-8,11,13-triene **(8)** [600 MHz, $CDCl_3$, ppm].

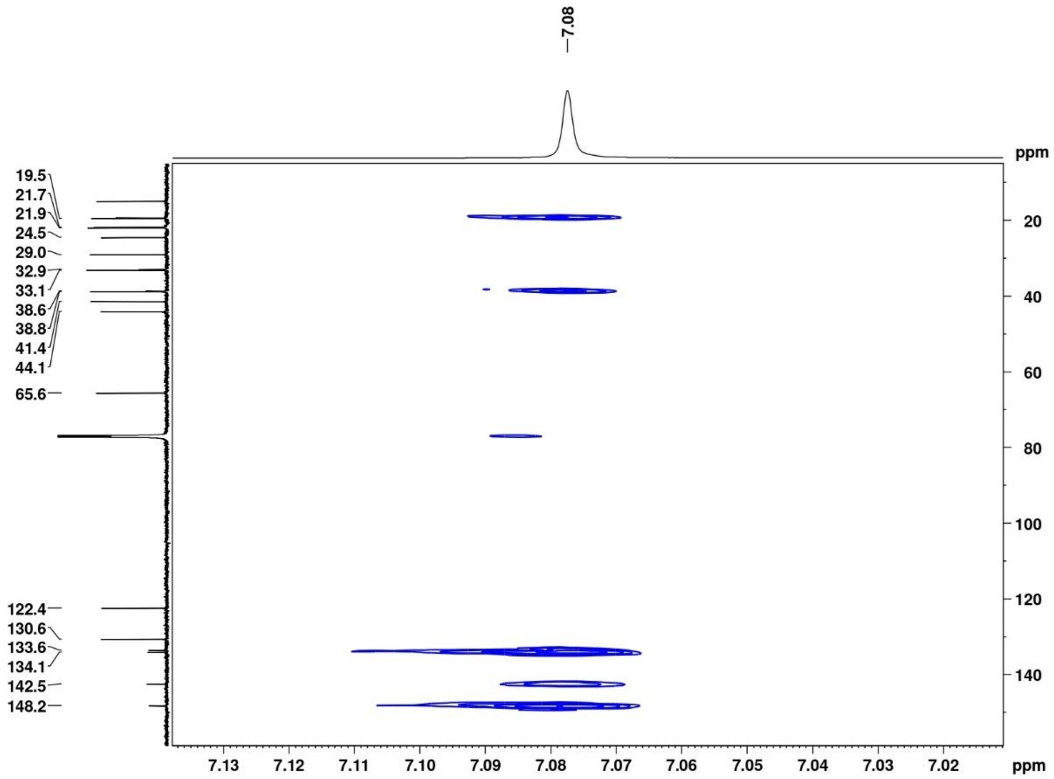

Supplementary Figure 128. HMBC NMR spectrum of 7β-hydroxy-clesitantha-8,11,13-triene **(8)** [600 MHz, $CDCl_3$, ppm].

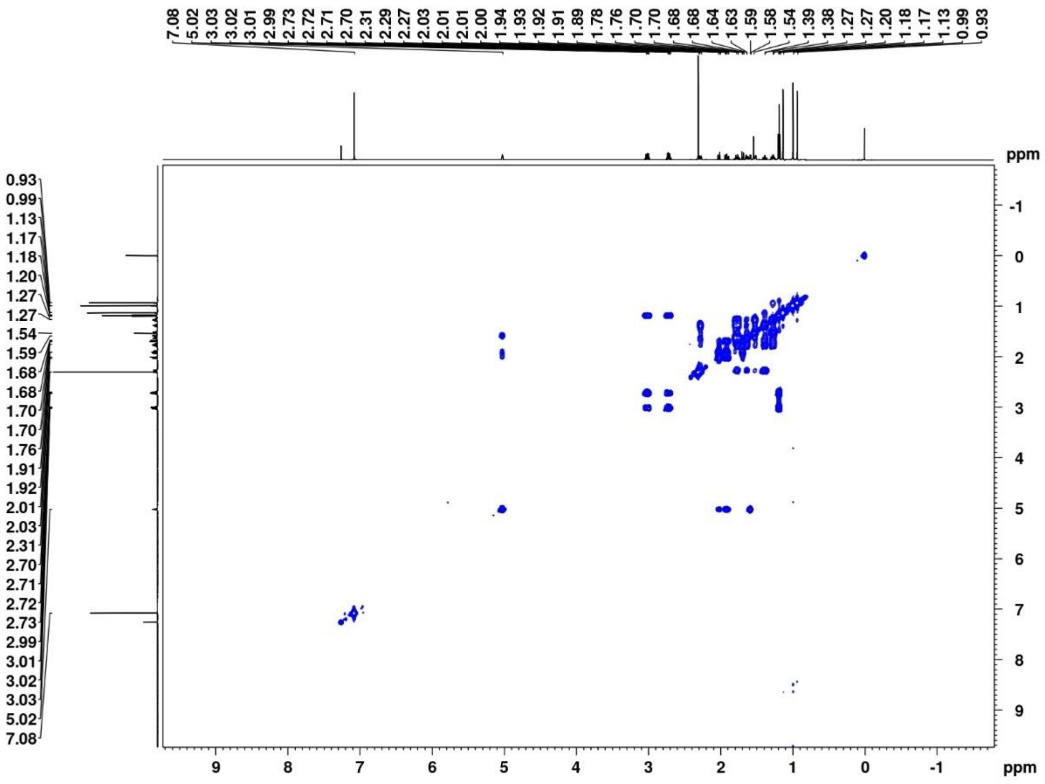

Supplementary Figure 129. COSY NMR spectrum of 7β-hydroxy-clesitantha-8,11,13-triene **(8)** [600 MHz, $CDCl_3$, ppm].

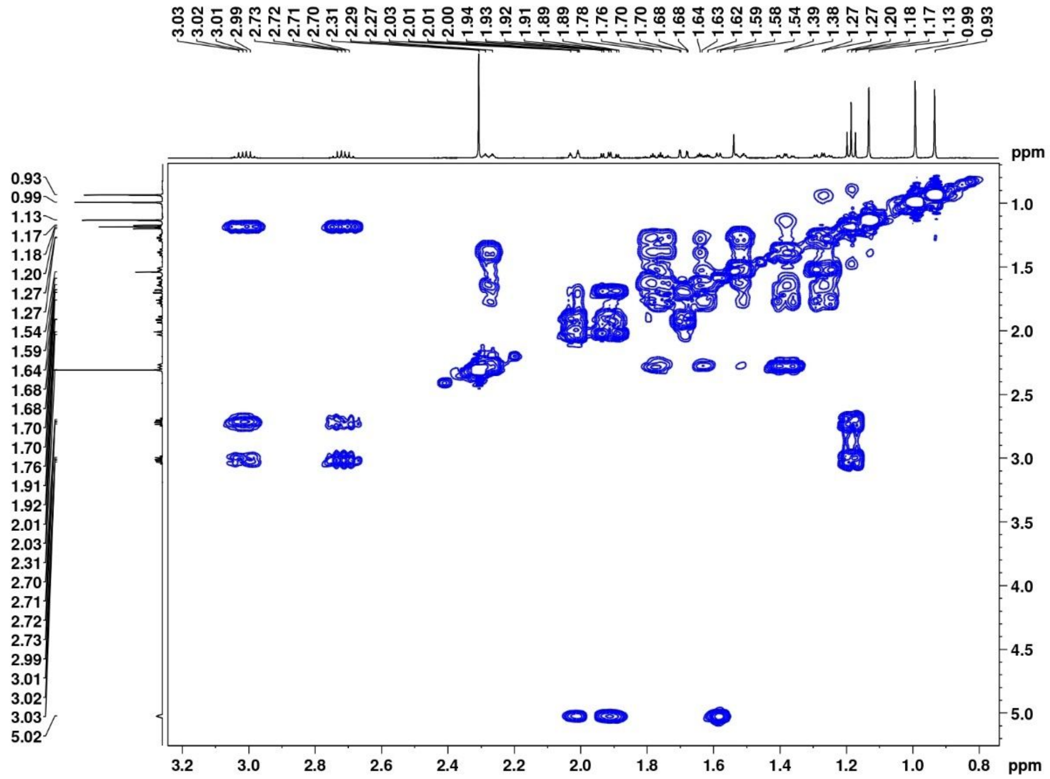

Supplementary Figure 130. COSY NMR spectrum of 7β-hydroxy-clesitantha-8,11,13-triene **(8)** [600 MHz, $CDCl_3$, ppm].

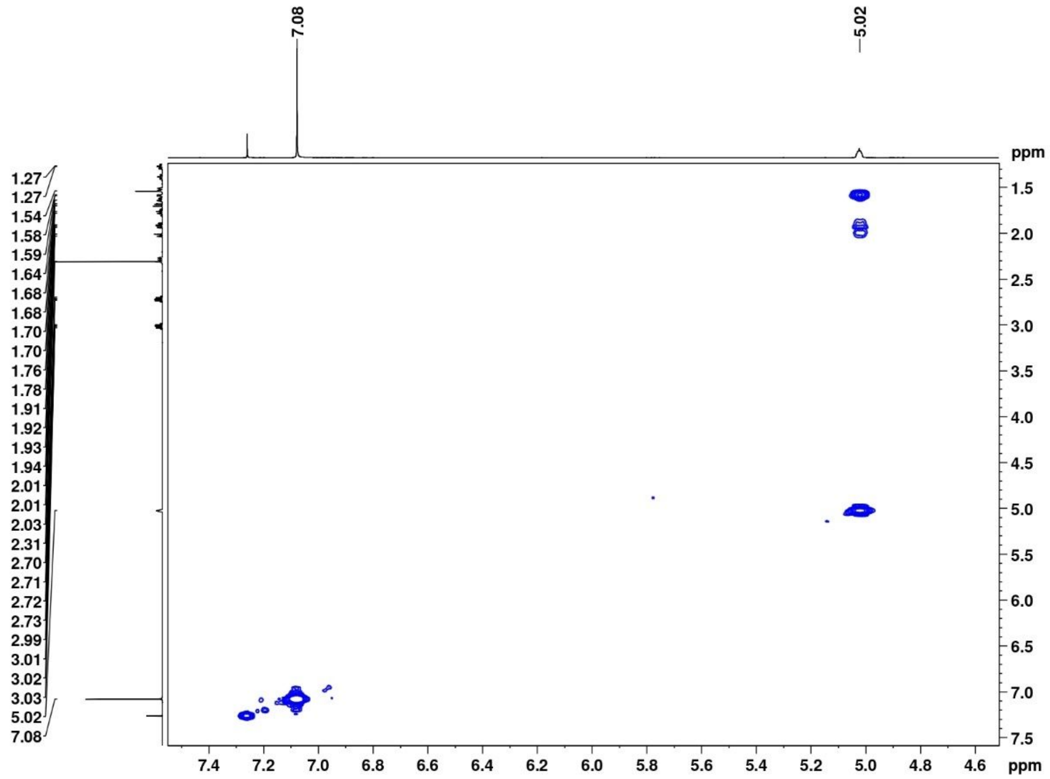

Supplementary Figure 131. COSY NMR spectrum of 7β-hydroxy-clesitantha-8,11,13-triene **(8)** [600 MHz, $CDCl_3$, ppm].

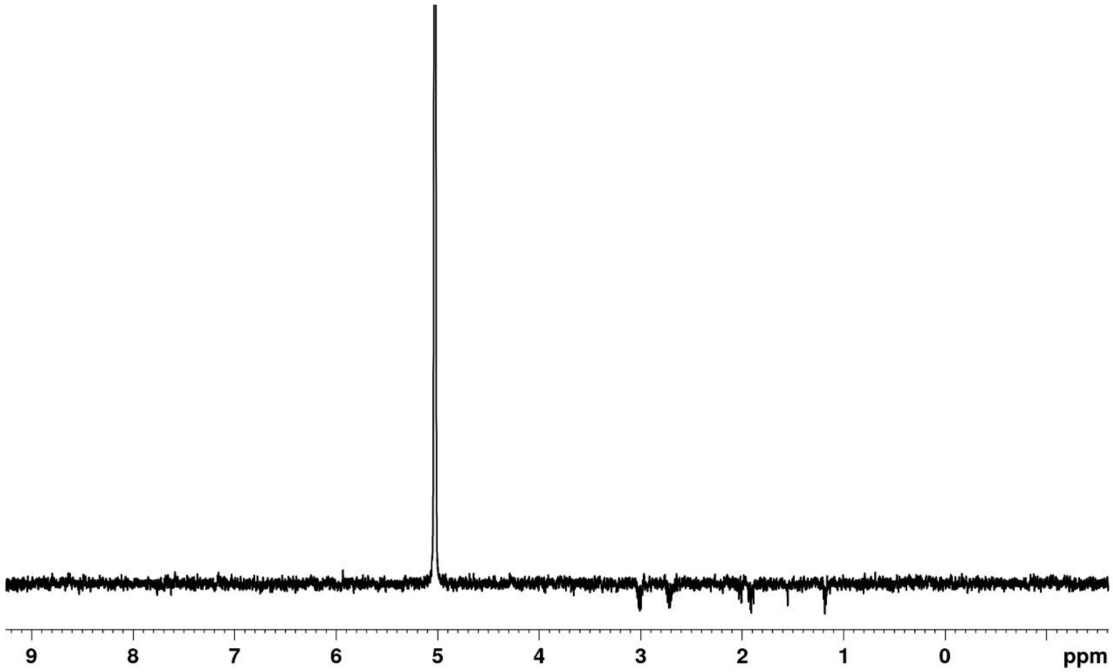

Supplementary Figure 132. gNoesy NMR spectrum of 7β-hydroxy-clesitantha-8,11,13-triene **(8)** [600 MHz, $CDCl_3$, ppm].

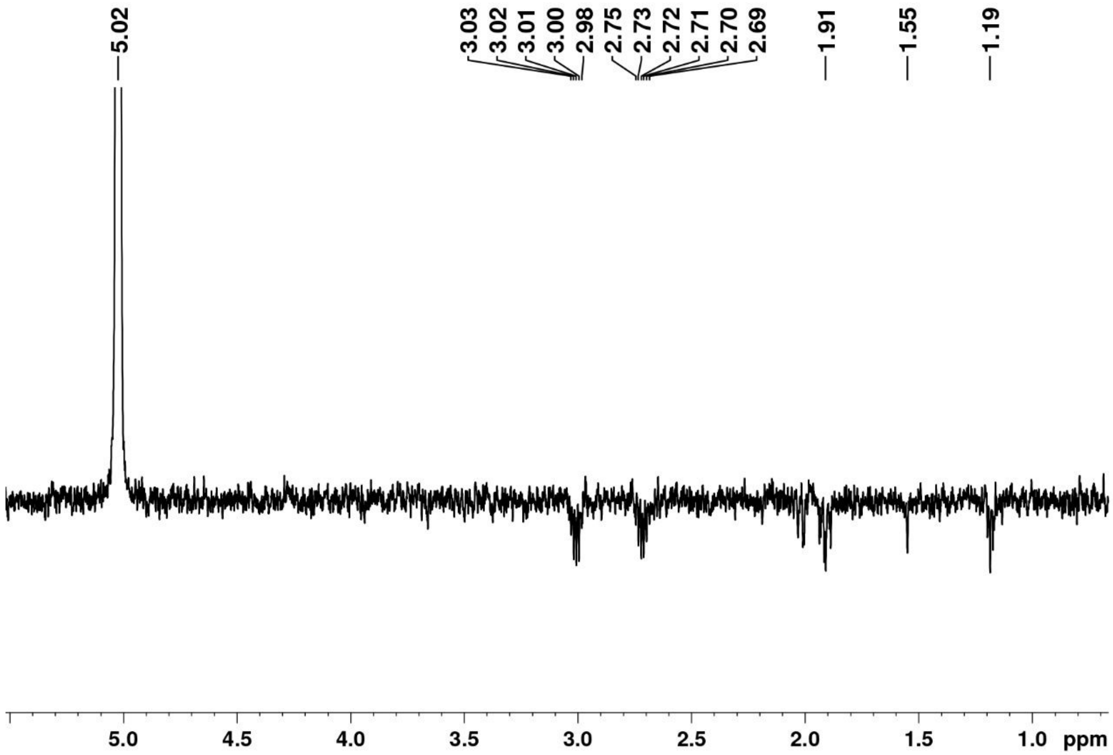

Supplementary Figure 133. gNoesy NMR spectrum of 7β-hydroxy-clesitantha-8,11,13-triene **(8)** [600 MHz, $CDCl_3$, ppm].

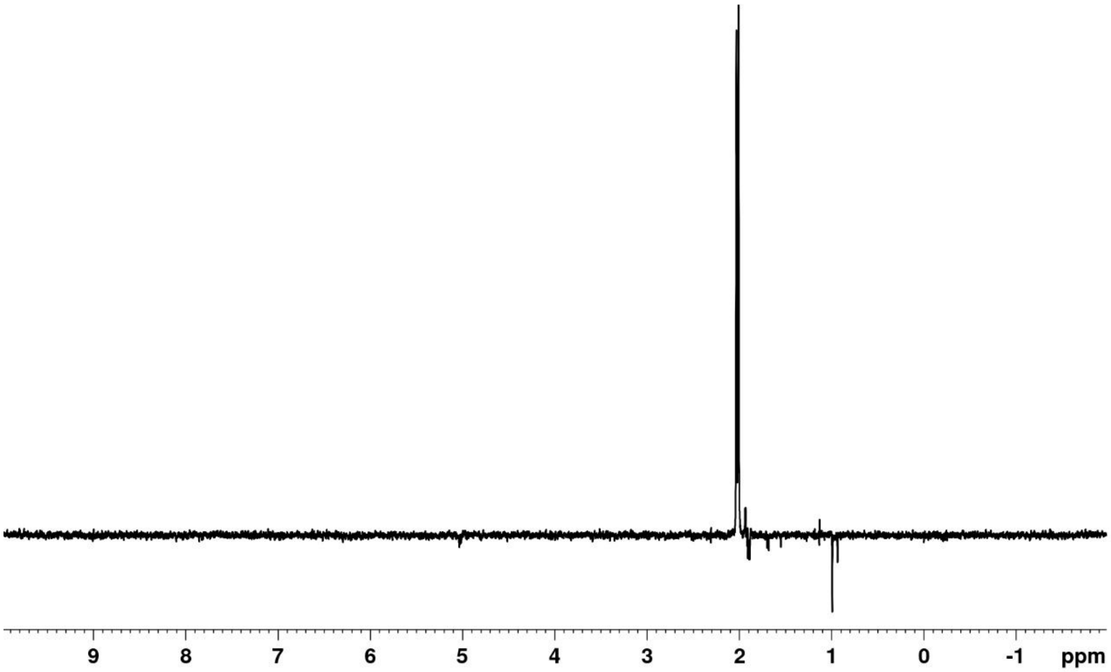

Supplementary Figure 134. gNoesy NMR spectrum of 7β-hydroxy-clesitantha-8,11,13-triene **(8)** [600 MHz, $CDCl_3$, ppm].

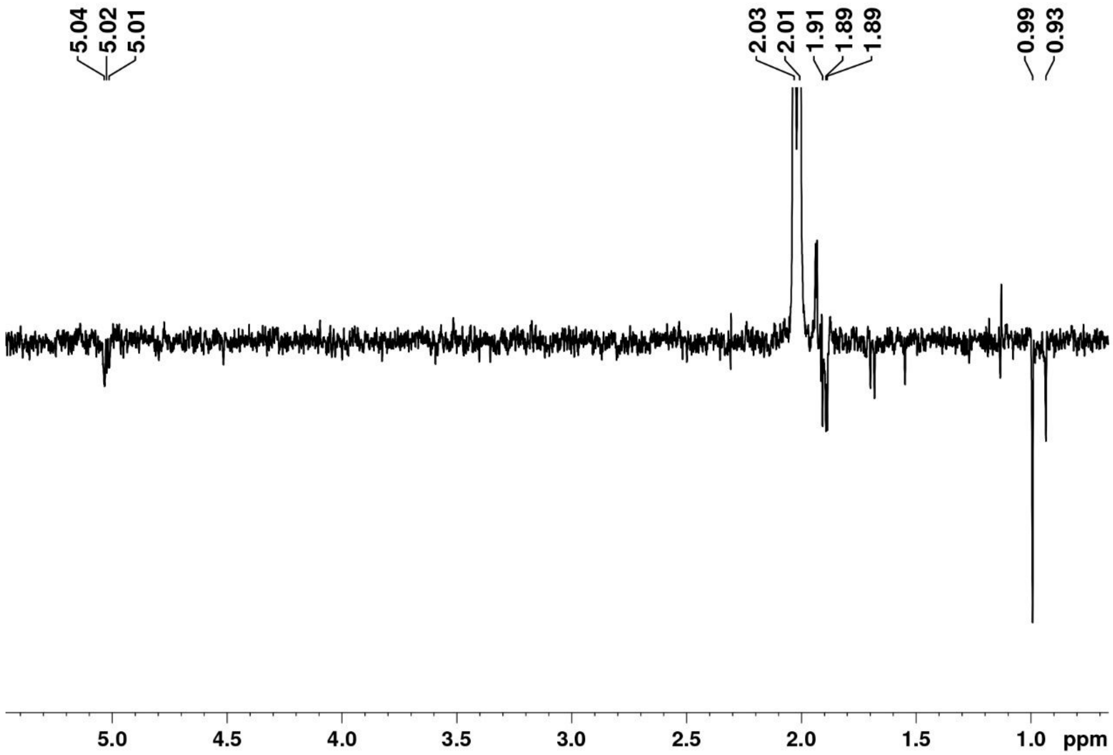

Supplementary Figure 135. gNoesy NMR spectrum of 7β-hydroxy-clesitantha-8,11,13-triene **(8)** [600 MHz, $CDCl_3$, ppm].

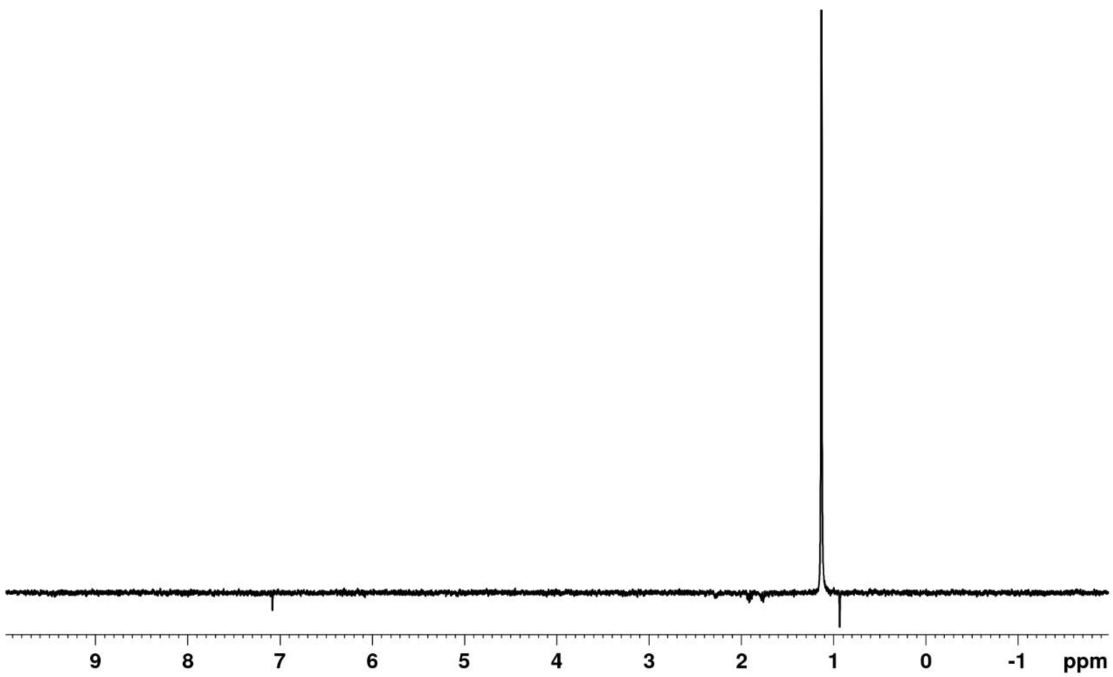

Supplementary Figure 136. gNoesy NMR spectrum of 7β-hydroxy-clesitantha-8,11,13-triene **(8)** [600 MHz, $CDCl_3$, ppm].

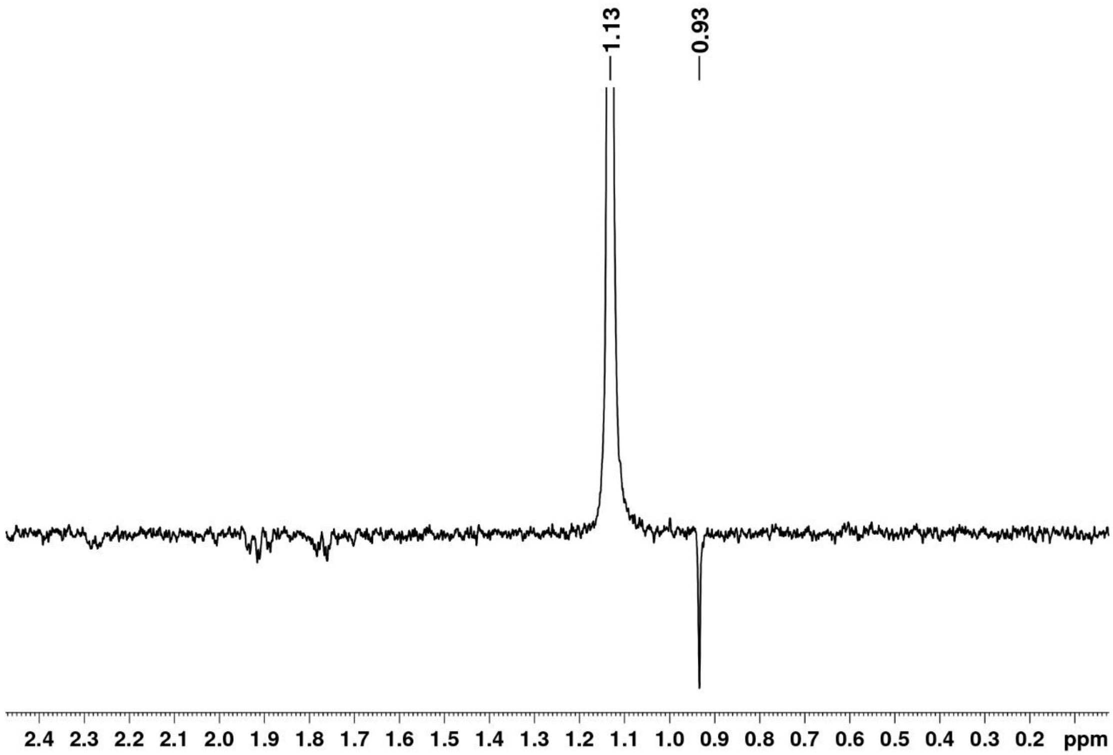

Supplementary Figure 137. gNoesy NMR spectrum of 7β-hydroxy-clesitantha-8,11,13-triene **(8)** [600 MHz, $CDCl_3$, ppm].

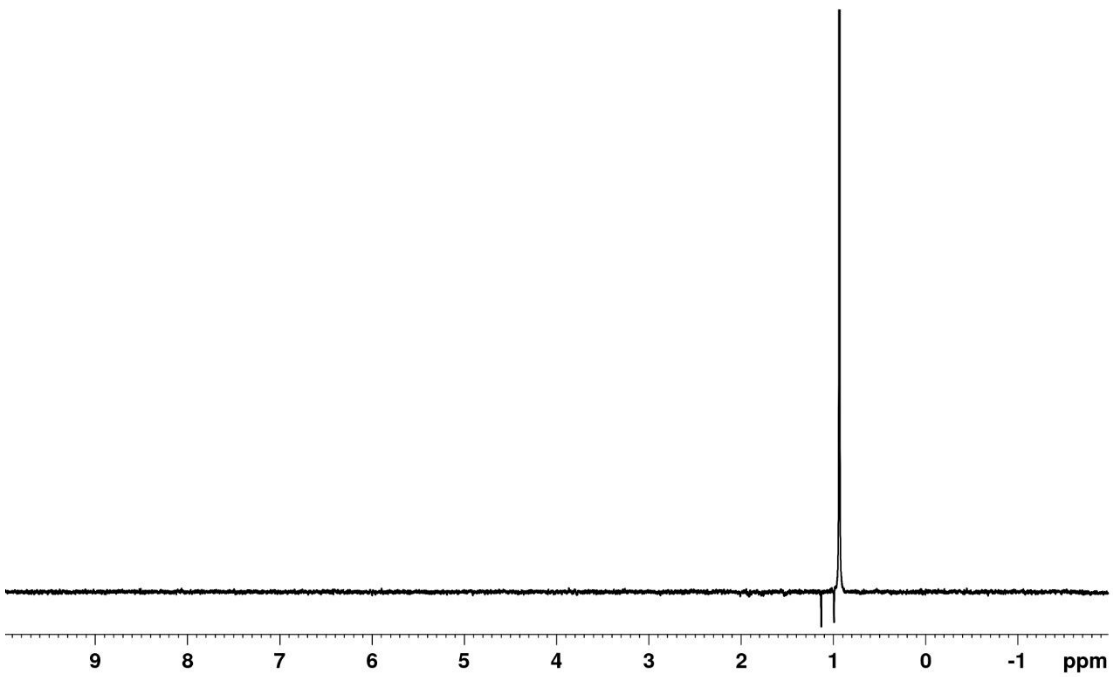

Supplementary Figure 138. gNoesy NMR spectrum of 7β-hydroxy-clesitantha-8,11,13-triene **(8)** [600 MHz, $CDCl_3$, ppm].

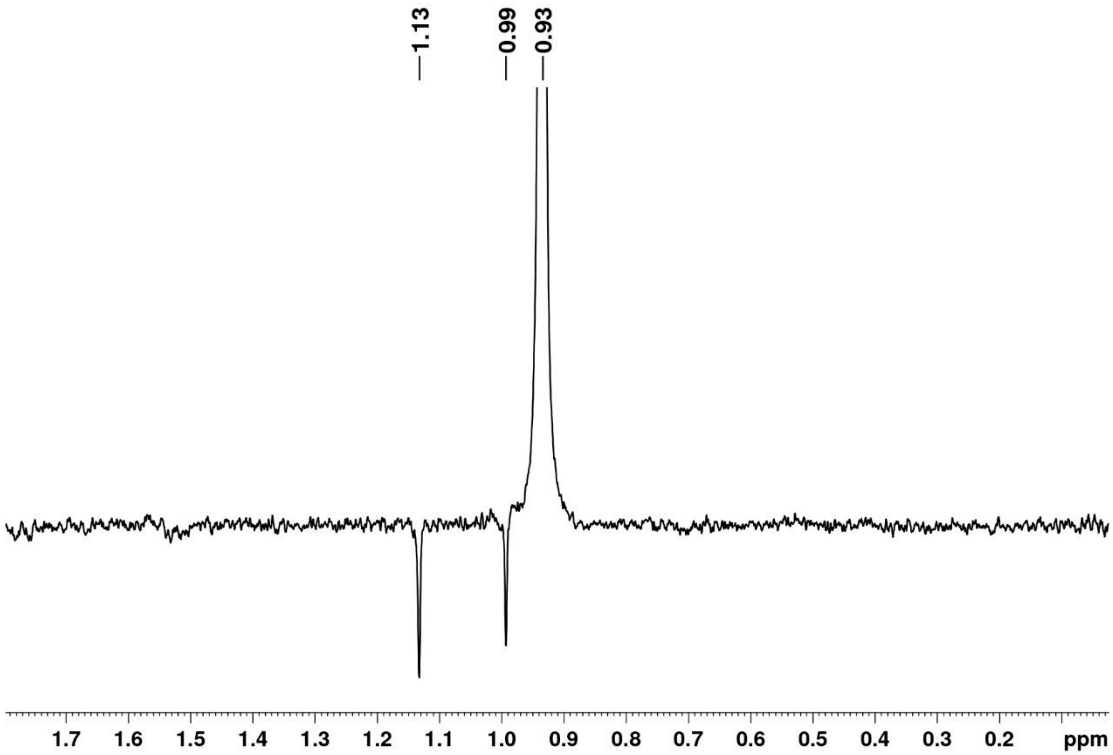

Supplementary Figure 139. gNoesy NMR spectrum of 7β-hydroxy-clesitantha-8,11,13-triene **(8)** [600 MHz, $CDCl_3$, ppm].

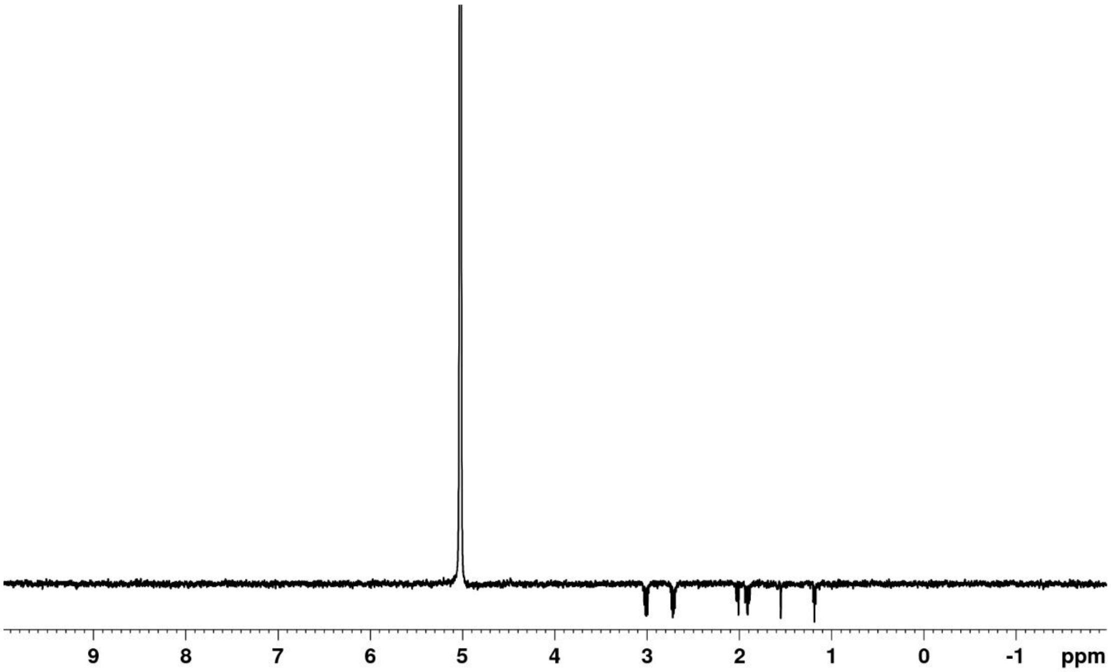

Supplementary Figure 140. gNoesy NMR spectrum of 7β-hydroxy-clesitantha-8,11,13-triene **(8)** [600 MHz, $CDCl_3$, ppm].

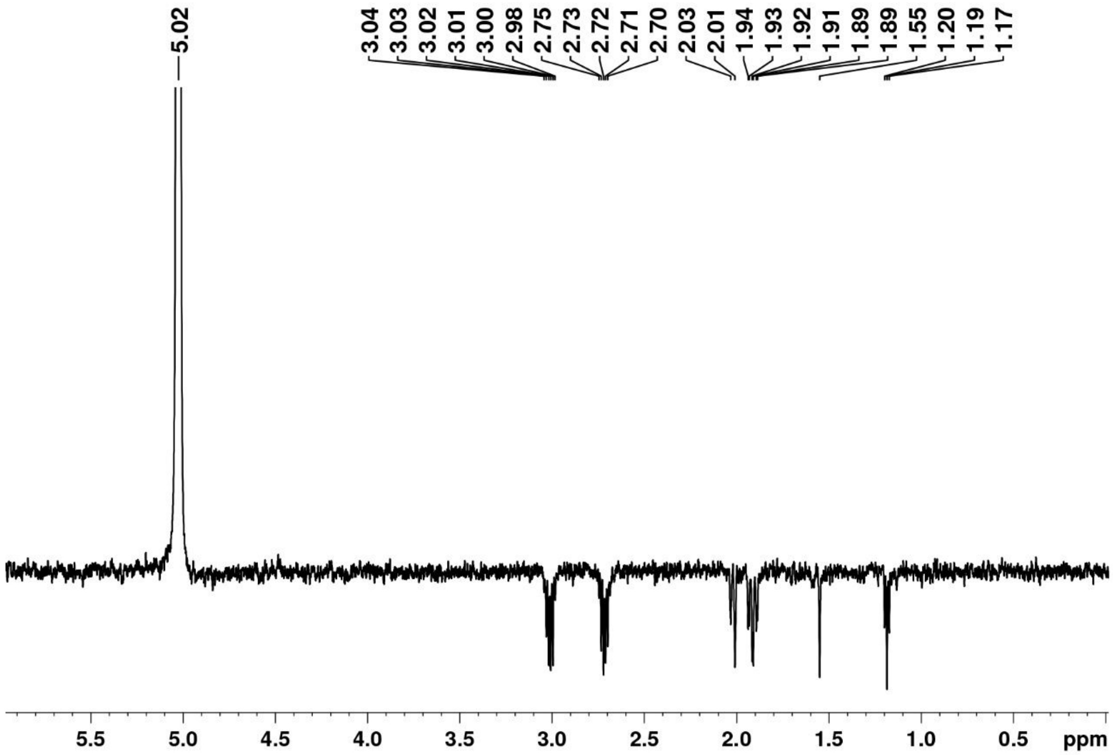

Supplementary Figure 141. gNoesy NMR spectrum of 7β-hydroxy-clesitantha-8,11,13-triene **(8)** [600 MHz, $CDCl_3$, ppm].

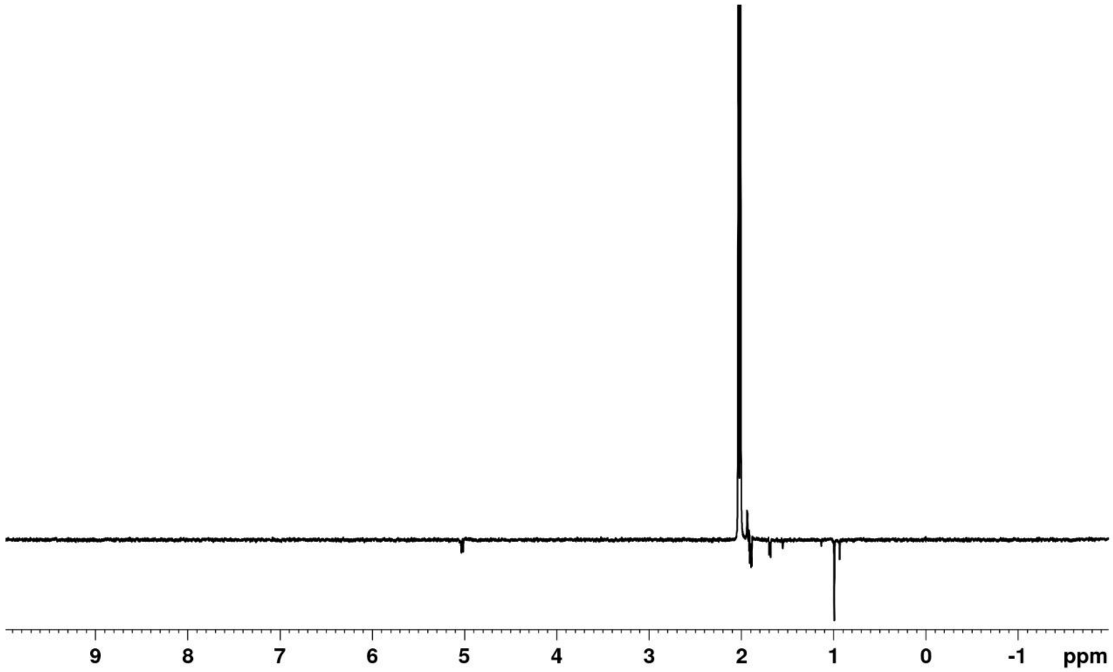

Supplementary Figure 142. gNoesy NMR spectrum of 7β-hydroxy-clesitantha-8,11,13-triene **(8)** [600 MHz, $CDCl_3$, ppm].

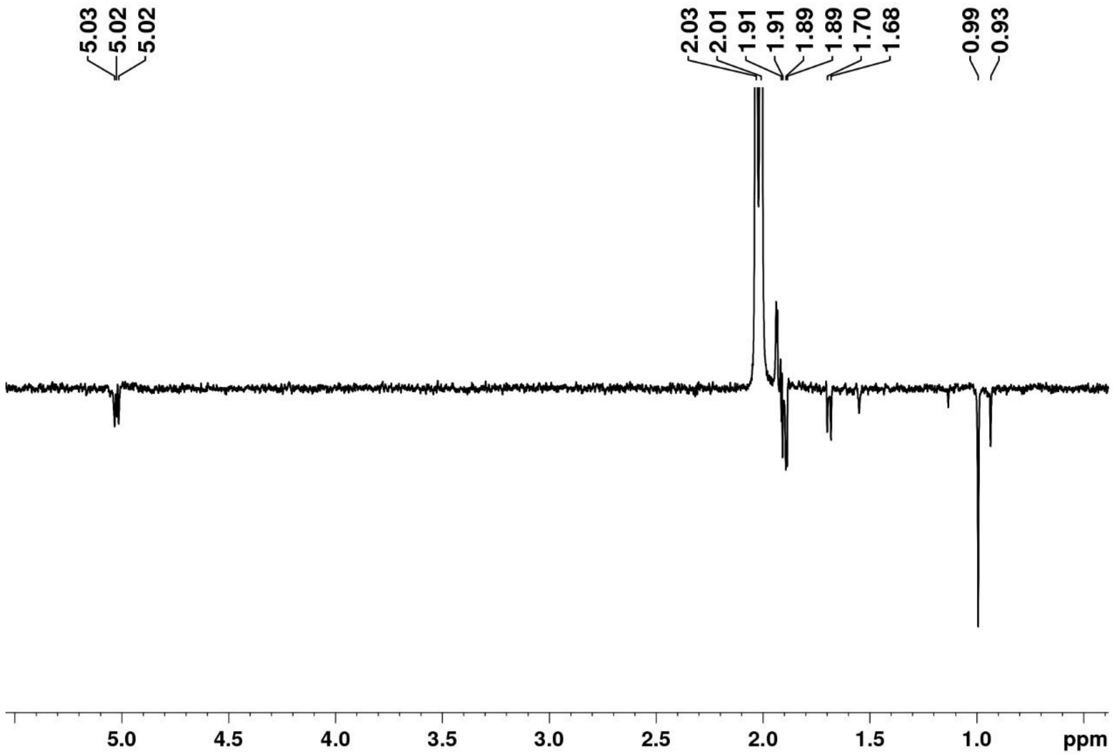

Supplementary Figure 143. gNoesy NMR spectrum of 7β-hydroxy-clesitantha-8,11,13-triene **(8)** [600 MHz, $CDCl_3$, ppm].

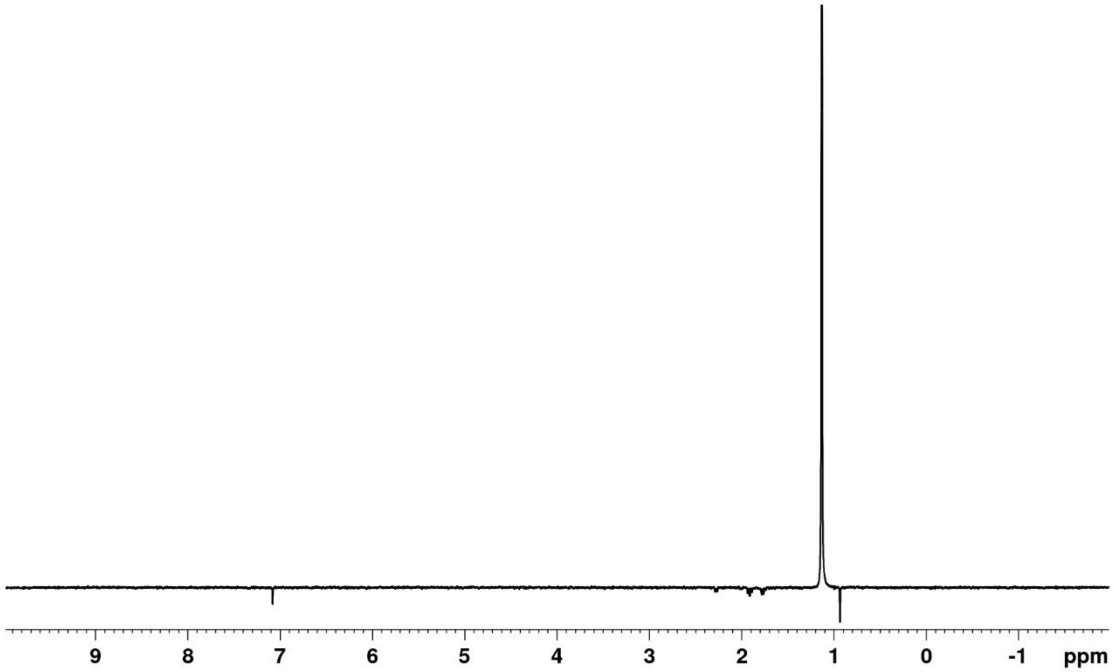

Supplementary Figure 144. gNoesy NMR spectrum of 7β-hydroxy-clesitantha-8,11,13-triene **(8)** [600 MHz, $CDCl_3$, ppm].

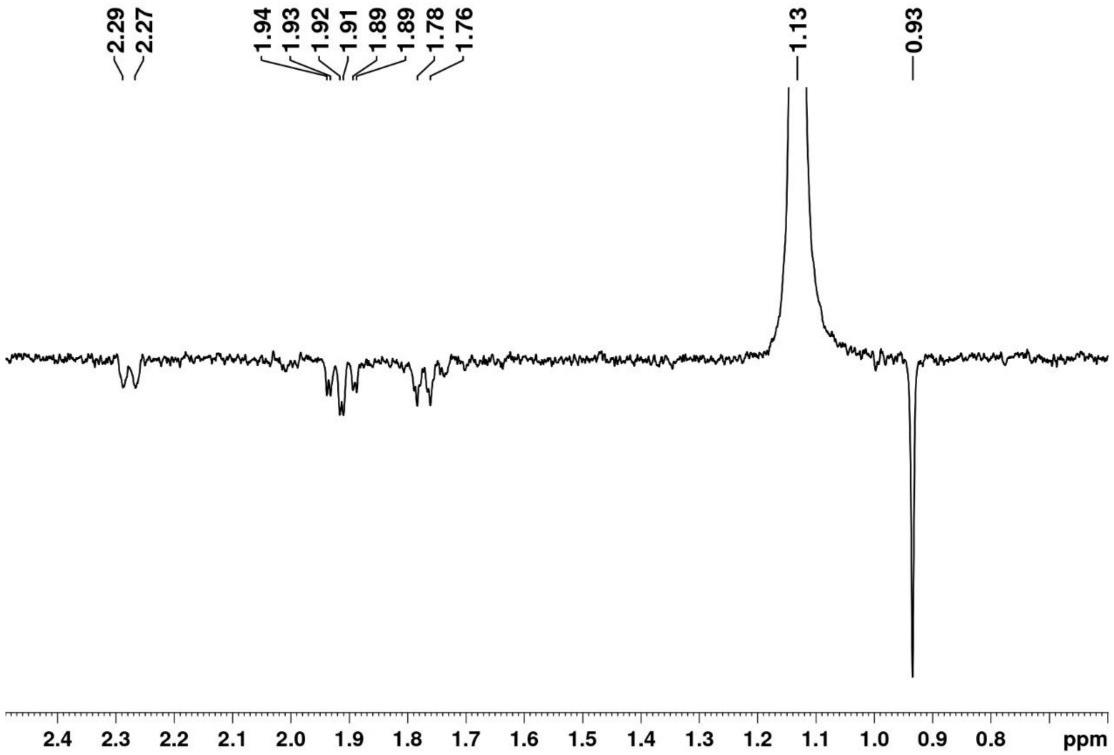

Supplementary Figure 145. gNoesy NMR spectrum of 7β-hydroxy-clesitantha-8,11,13-triene **(8)** [600 MHz, $CDCl_3$, ppm].
